# Sequence properties of certain GC rich avian genes, their origins and absence from genome assemblies: case studies

**DOI:** 10.1186/s12864-019-6131-1

**Published:** 2019-10-14

**Authors:** Linda Beauclair, Christelle Ramé, Peter Arensburger, Benoît Piégu, Florian Guillou, Joëlle Dupont, Yves Bigot

**Affiliations:** 1 PRC, UMR INRA0085, CNRS 7247, Centre INRA Val de Loire, 37380 Nouzilly, France; 2 Biological Sciences Department, California State Polytechnic University, Pomona, CA 91768 USA

**Keywords:** G-quadruplex/genome/Illumina/ PacBio/repeats

## Abstract

**Background:**

More and more eukaryotic genomes are sequenced and assembled, most of them presented as a complete model in which missing chromosomal regions are filled by Ns and where a few chromosomes may be lacking. Avian genomes often contain sequences with high GC content, which has been hypothesized to be at the origin of many missing sequences in these genomes. We investigated features of these missing sequences to discover why some may not have been integrated into genomic libraries and/or sequenced.

**Results:**

The sequences of five red jungle fowl cDNA models with high GC content were used as queries to search publicly available datasets of Illumina and Pacbio sequencing reads. These were used to reconstruct the leptin, TNFα, MRPL52, PCP2 and PET100 genes, all of which are absent from the red jungle fowl genome model. These gene sequences displayed elevated GC contents, had intron sizes that were sometimes larger than non-avian orthologues, and had non-coding regions that contained numerous tandem and inverted repeat sequences with motifs able to assemble into stable G-quadruplexes and intrastrand dyadic structures. Our results suggest that Illumina technology was unable to sequence the non-coding regions of these genes. On the other hand, PacBio technology was able to sequence these regions, but with dramatically lower efficiency than would typically be expected.

**Conclusions:**

High GC content was not the principal reason why numerous GC-rich regions of avian genomes are missing from genome assembly models. Instead, it is the presence of tandem repeats containing motifs capable of assembling into very stable secondary structures that is likely responsible.

## Background

The red jungle fowl (RJF) is the ancestor of all domestic chicken breeds and lines, and was the third vertebrate to have its genome sequenced and assembled [1]. In the last decade the increased output and reliability of second and third generation high throughput sequencing technologies have led to the release of five RJF genome model updates, the galGal5 model is currently the most commonly used.

The galGal5 model [2] is organized into 34 chromosomes that are split into 9 macro-autosomes and 23 micro-autosomes based on their size in caryology, and 2 sexual chromosomes, the W and Z micro and macro heterosomes respectively. Together, these chromosome models are approximately 1 Giga base pairs (Gbp) in size. An additional 0.23 Gbp of sequences is also present in the model either as unplaced scaffolds or as scaffolds assigned to a chromosome but not placed within the chromosome. Models for microchromosomes 29, 30, 34, 35, 36, 37 and 38 are not available, perhaps because many of their sequences are found in scaffolds without a precise location in the genome. The current galGal5 model size (total size, 1.23 Gbp) is smaller than the size estimated by cytometry and DNA reassociation kinetics (ranging from 1.28–1.3 Gbp see [3]). This suggests that at least 3.4 to 5.4% of the RJF genome remains to be sequenced and assembled, including some subtelomeric regions (see [4]). During the preparation of this manuscript the first files of the galGal6 model were released (March 27th 2018) as well its annotation at NCBI (May 18th 2018) and Ensembl (March 11th 2019). galGal6 has a similar total genome size as galGal5, but includes a longer chromosome 16 (we would note that this is probably the most ellusive avian chromosome due to its repeat content), a chromosome 30, and fewer unplaced scaffolds than galGal5. Because the galGal6 genome model was only very recently released, the galGal5 was used as the cornerstone of our study while galGal6 was used as a complementary information source.

In addition to the genome completeness questions discussed above, there has been significant controversy in the literature regarding at least 2454 genes in this animal [5]. These genes are present in other vertebrates but have been either lost during the evolution of the bird lineage [6–9], or are “invisible” to sequencing and assembling technologies because of their elevated GC content. One of the best examples concerns the sequence of leptin gene in RJF and other avian genomes. This has been the subject of several controversial publications during the last two decades (for reviews see [10, 11]). In 2016 the controversy was resolved with the publication of one partial model and one complete sequence model of the leptin open reading frames (ORFs) in two Galloanserae species. These models (accession numbers LN794246 and LN794245) were reconstructed from nine 454 reads from the RJF (partial model) and from 20 Illumina reads from the Pekin duck *Anas platyrhynchos domesticus* (complete model) [10]. These sequences confirmed that the coding exons of leptin genes of both species had elevated GC content (67.7 and 73.3%, respectively). This observation likely explains why these genes had long been difficult to amplify by polymerase chain reactions (PCR) or to sequence (the difficulty of using these techniques with GC rich sequences is discussed in [12–17]). Sequencing confirmed that there were 2 coding exons in both species; which had previously been observed in a dove, two falcons, a tit and a zebra finch species [9]. Recently, the locus containing the leptin gene was mapped on the galGal5 RJF genome model to the distal tip of the p arm on chromosome 1, a subtelomeric region known to be GC-rich [4]. A second publication in 2016 released the full-length sequence of the RJF leptin ORF (accession number KT970642). This sequence has been verified by sequencing of three tilled RT-PCR products obtained under non-routine amplification conditions [18]. In the last 3 years several sets of avian cDNA corresponding to “lost or missing GC-rich genes” have been published ([19], 14 cDNAs [5, 20];, 2132 cDNAs [21, 22];, 1 cDNA). These publications support the hypothesis that the current sequencing and assembly technologies are inefficient in regions with elevated GC content (> 60%).

In addition to the issues raised above, there are at least four categories of genes that were at one point considered to be absent from avian genomes. First are genes where public RNA-seq data from chickens allowed for reconstruction of cDNAs, but for which no complete or partial genomic gene sequences are available. The RJF leptin and tumor necrosis factor α (TNFα) genes [11, 22] belong to this category. In the second category are genes that have yet to be detected by searching public datasets of RJF RNA-seq or by genome resequencing, but for which cDNA or genomic copies are available or can be easily characterized within sequence data of other avian species [5]. An example is the omentin gene (so-called intellectin 1, ITLN1). Indeed, an ITLN1 cDNA is available in the transcriptome sequence of the tinamou (*Tinamus guttatus*, accession XP_010211902.1). Furthermore, this has allowed for characterization of an orthologous gene in the kiwi (*Apteryx australis mantelli*, Additional file 1). A third category of genes that were thought be absent in birds are genes for which no trace has been found in public sequencing datasets. Examples of such genes are those encoding the kiss peptides 1 and 3 and their related receptors [23], as well as the piwi 3 and piwi 4 proteins [24]. It is likely this category includes some true losses in the avian lineage, but some genes are likely difficult to sequence and assemble using current technologies. The last category is simply those genes that have already been sequenced, assembled, and mapped to scaffolds but are not properly annotated. Examples include genes coding for the carnitine O-palmitoyltransferase 1 (CPT1C) and insulin-like peptide (ISNL5) (Additional file 2). A final note, genes in the first three categories may be absent at least in part, due to technical difficulties such as preserving their sequences following genome fragmentation, difficulty amplifying them by PCR, or difficulty sequencing them in RNA-seq and/or genomic libraries, or a combination of these.

Because PacBio technology is deemed to be more efficient at sequencing GC-rich fragments [25] than Illumina technology, we have searched public PacBio datasets for sequences containing the coding exons of some “lost or missing GC-rich genes” [5, 19–22] in order to identify sequence properties that might explain their absence from genome models. We first evaluated the quality of sequences obtained with PacBio technology for GC rich gene sequences. We then verified whether these genes were still absent from the two most recent galGal genome models. Following this, we searched public datasets in order to construct a genomic model of the leptin gene, an important contribution to avian physiologists. This model was extremely GC-rich, had an intron composed of short tandem repeats, and contained numerous stretches of G-motifs in both coding and non-coding regions. We found that these motifs would be favorable for the assembly of intrastrand G-quadruplexes (G4 s) [26–30]. These are nucleotidic structures that are extremely stable and reluctant to RNA and DNA-template dependent DNA replication. Because of the leptin gene’s unusual properties we identified it in other avian species and studied its expression. Finally, in order compare the leptin gene properties to other genes that are difficult to sequence, we extended our observations to four other genes encoding the tumor necrosis factor alpha (TNFα), the mitochondrial ribosomal protein L52 (MRPL52), the Purkinje cell protein 2 (PCP2) and the protein homolog to the yeast mitochondrial PET100 protein (PET100), for which reliable cDNA sequences are currently only available in [19, 22]. Similar to the leptin gene, we chose these four genes precisely because they are among the most difficult to assemble into cDNA models. Indeed, their DNA sequences are not sufficiently conserved for interspecific comparisons between bird species (as was done the remaining 2132 cDNAs [21, 22]). Therefore, a description of gene copy numbers using Pacbio reads is currently the best way to confirm their presence in the RJF genome.

## Results

PacBio technology has been demonstrated to be more efficient for sequencing genomic regions that Illumina machines struggle with [22, 31, 32]. However, it remains unclear if this applies to sequence fragments with > 60% GC content; specifically whether read depth sequence errors are within acceptable limits using PacBio technology. Furthermore, at the same time as this manuscript was reviewed another study was published [33] which evaluated the efficiency of PacBio reads for sequencing non-B DNA regions in human and mouse genomes. This study showed that non-B DNA regions modified the polymerization speed and the error rate, i.e. they decreased the reliability of Pacbio reads obtained from these specific regions. Second, they showed that non-B DNA regions (i.e. regions able to determine non-B structures plus their single-stranded DNA environment due to transitions between B and non-B DNA) represent about 13% of the studied genomes [34, 35]. Among the seven kinds of non-B DNA determinants that were investigated, they showed [33] that mammalian regions containing stretches of direct repeats and were composed mostly of G-quadruplexes (G4; i.e. regions containing the motif G_3_ + _N1-12_G_3_ + N_1-12_G_3_ + N_1-12_G_3_ where N is any base including G) had pronounced effects on read depth and the error rate. Here, the impact of non-B DNA on RJF data was investigated.

### PacBio reads and RJF GC-rich sequences

In an effort to benchmark the reliability of the PacBio technology on RJF GC-rich sequences, we used two RJF GC-rich gene models: 1) a region coding the 18S–5.8S-28S ribosomal RNA (rDNA) which is transcribed from clustered genes repeated in tandem on chromosome 16 [36] and 2) the 5 exon gene encoding synaptic vesicle glycoprotein 2A (SV2A) which is located on microchromosome 25 [37]. Using these two sequences as queries we searched the two RJF PacBio projects (accession numbers SRR2028042-SRR2028057 and SRR2028138-SRR2028233, 190 Gb., ~180X genome coverage, PacBio RS 2.3.0.0.140640) and SRR5444488-SRR5444513 (63 Gb., ~60X genome coverage, PacBio RS II 2.3.0.3.154799) that were available as databases for the the RJF.

Our approach for this benchmarking effort was to compare the percent of PacBio reads we could match to the ribosomal and SV2A gene sequences using blastn and blasr, to the expected value based on the existing genome assembly and PacBio read genome coverage. For the ribosomal gene sequence (accession number KT445834, 11,863 bp, 71.09% GC, repeated 350 times per genome [38]) we found that only 37,925 kbp of the PacBio read sequences matched the gene sequence (identity match threshold 75% and above), while we expected 747,609 bp (i.e. about 5% of the expected value) for a PacBio data set with 180X coverage. The equivalent numbers for the SV2A gene (accession NC_006112.4 from positions 1,851,072 to 1,865,776, 14,705 bp, 62.25% GC, single copy gene) were 294,881 bp coverage while we expected 2,646,720 bp (i.e. about 11% of the expected value). This test was repeated on a second PacBio dataset with lower (60X) coverage with similar results (98,755 bp coverage while we expected 882,300 bp, that is approximately 11.2% of the expected value). In addition to this depletion in coverage, it should be noted that the read alignment within these two sequences were very patchy, some regions of the KT445834 sequence were not even covered.

This indicated that for these two GC-rich sequence, queries were found 10–20 fold below what would be expected in PacBio datasets. When similar searches were performed using the CPT1C and RNL3 gene sequences (36.24 and 54.24%GC respectively) no such coverage deficit was observed. With respect to sequence error rates, we observed that over the entire length of the CPT1C and RNL3 genes, it was close to that previously described when benchmarking this technology (error rate ranging from 11 to 15%, depending on the sequence; for review see [22]). This was in striking contrast to the rDNA and SV2A genes for which error rates were between 15 to 25% (because the sequences were GC rich, sequences with error rates above 25% could not be properly aligned [33]). Furthermore, we observed that only certain regions in long reads had an error rate below 25%. This explained why we obtained fragmented hits with these PacBio reads, regions in these reads with error rate above 25% being impossible to align.

### Presence of cDNA models missing in public datasets related to galGal5 and galGal6 except in Pacbio reads

**Fig. 1:**
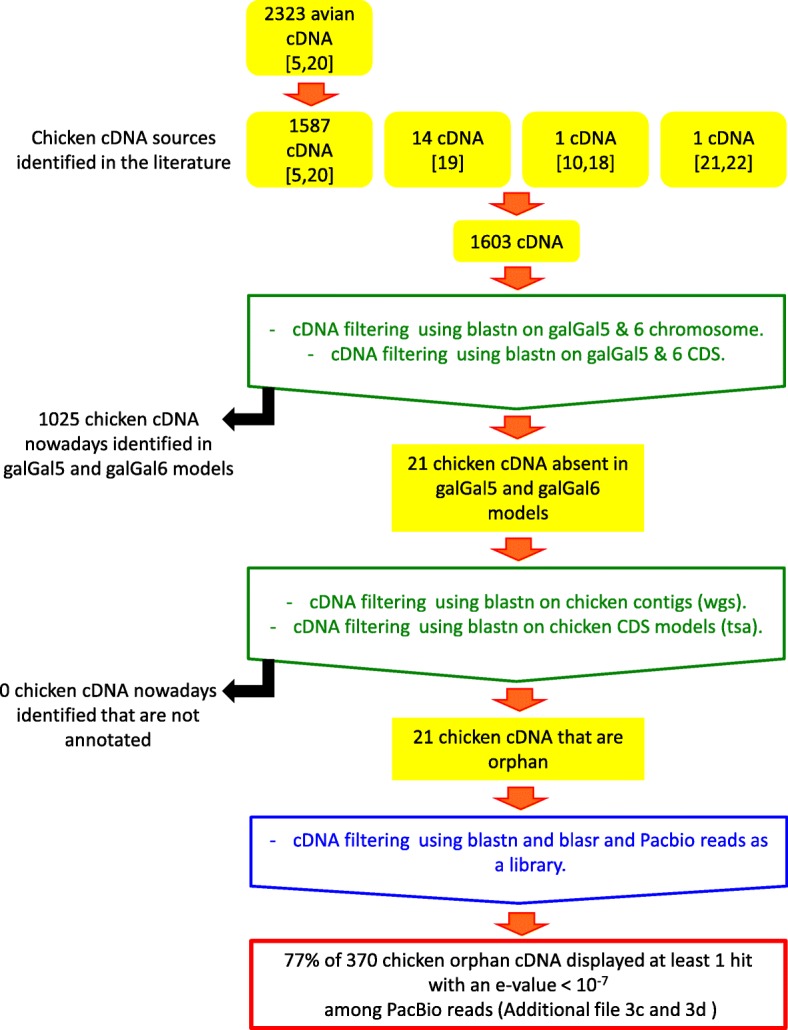
Analysis process used to filter orphan cDNA available in the literature and to identify the presence among Pacbio reads in databases

In order to evaluate the number of existing orphan cDNA models, we verified the status of those described in the literature during the last 5 years in the most recent releases of the RJF genome and cDNA models (Fig. 1). We first gathered several sources of cDNA sequences that were previously validated [5, 19–22] as being absent from avian models and having an average GC content above that of genes in the galGal4 model [5, 19–22]. The majority of these cDNAs originated from a batch of 2323 avian cDNA models that were characterized from public transcriptomic data based on their interspecific conservation. For 2132 of them (91.7%), the presence of an orthologous gene was verified in human (*Homo sapiens*) and the Chinese soft-shell turtle (*Pelodiscus sinensis*) whilst they were absent from the galGal4 model and from the collared flycatcher genome model (*Ficedula albicollis;* FicAlb_1.4 genome model [5, 20]; Additional file 3a). Of these models, 1587 were detected in chicken transcriptomic data [5, 20]. In addition, 16 other GC-rich chicken cDNA that were absent from the galGal5 and GalGal6 datasets were assembled from chicken transcriptomic data [5, 22].

Using Blast we looked for the presence of cDNAs for these 1603 genes in the most recent chicken datasets. Furthermore, we also examined: 1) whether each of these genes was annotated as a protein-coding sequence in galGal5 and galGal6, 2) whether each was present in the genomic sequence of galGal5 and galGal6 but not annotated as a CDS, and 3) whether each was present in the galGal5 and galGal6 CDS database from Ensembl (releases 94 and 96).

We found that only 1579 cDNA models were present in the four NCBI and Ensembl (galGal5 and galGal6) CDS sources and in the genomic sequences of galGal5 and galGal6. Twenty one of the 1603 chicken cDNA models were absent from the galGal5 and GalGal6 annotations, only two sets of cDNA models were missing from galGal5 (model 1515_GALgal) and galGal6 (1962_GALgal, 5115_GALgal) Ensembl CDS (Additional file 3b). Finally, we searched the whole genome sequence (WGS) and Transcription Shotgun Assembly (TSA) datasets at NCBI to verify whether some of these 24 chicken cDNA models might be present in other chicken sequencing projects. We found that none of these 24 chicken cDNA models were present in these datasets.

These 370 orphan models were then used as queries to search the two chicken PacBio projects mentioned above. We found that 17 of the 22 models (77%) had hits to both PacBio projects (Additional file 3c). With respect to the datasets used, these results highlighted the important progress achieved between the galGal4 and galGal6 models.

**Fig. 2:**
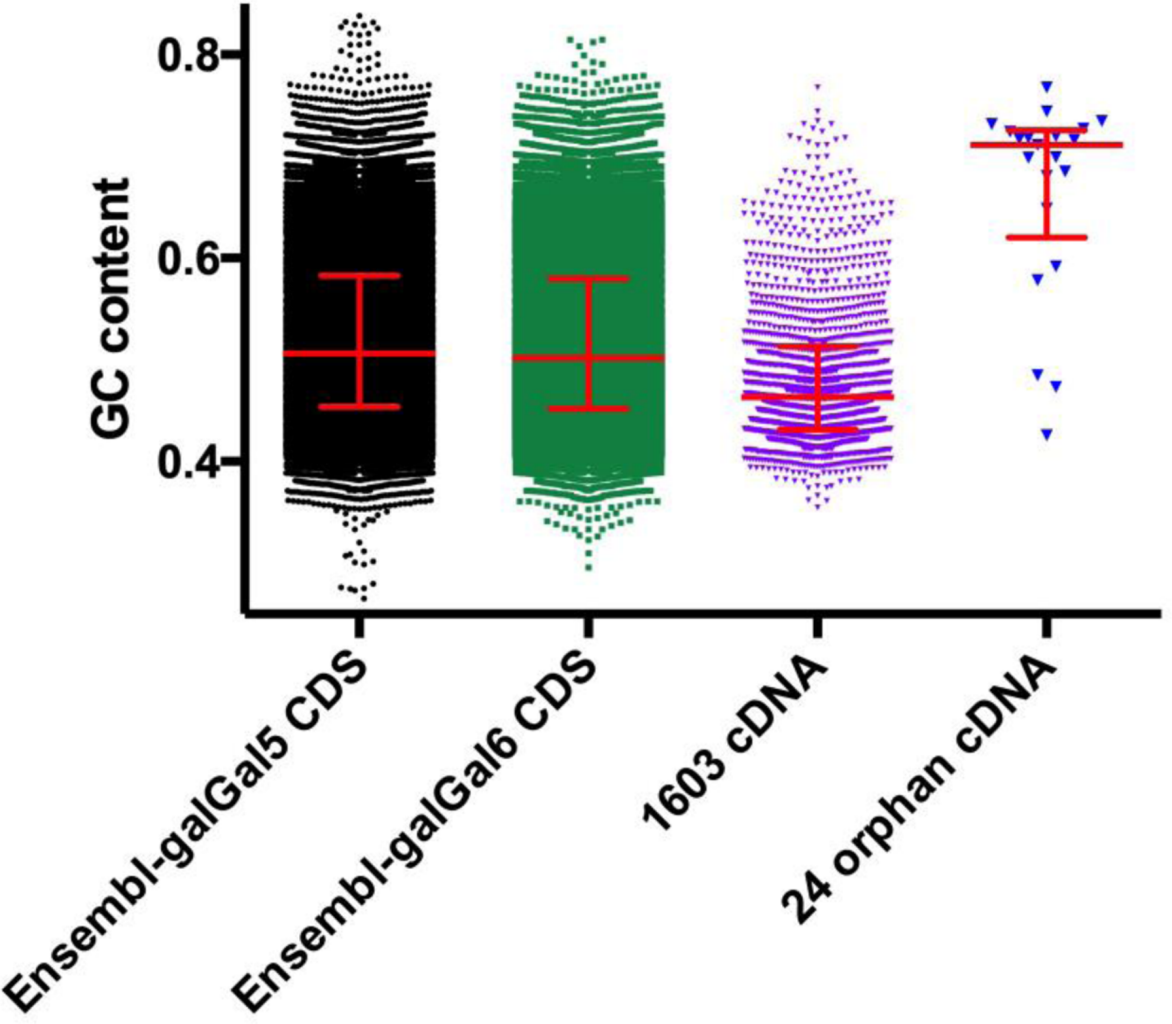
Distribution of GC contents of sequences contained in galGal5 and 6 ensembl CDS, the 1603 cDNA described in [5, 19–22], and the remaining 24 orphan cDNA in all *Gallus gallus* datasets. Red lines and bars located median, quartile 1 and 3 values

The analysis of the GC content of these 21 models was compared to those of CDS in the galGal5 and galGal6 Ensembl datasets and 1603 chicken cDNAs from [5, 19–22]. Results revealed that orphan cDNAs displayed a median GC content of about 70% while those of both Ensembl CDS datasets were about 52% (Fig. 2). However, it also revealed that there were two groups of cDNA models: 18 in the first cDNA group with sequences displaying GC content ranging from 60 to 80%; 3 in the second group of moderate GC content (42–49%; 1515_GALgal, 3153_GALgal, 8985_GALgal). Together, these results support that GC content was not the main factor explaining the absence of some genes in genome models assembled from deeply sequenced genomes and transcriptomes.

The above data led us to conclude that there is yet unexploited information within PacBio datasets to hunt for genes with GC rich sequences. To find these genes one needs a reliable set of cDNA sequence queries. An expected the most difficult part of this process is to align the PacBio sequence reads to each other over their entire length. For a typical chromosomal region, estimates are that a 15 pass coverage with PacBio reads or 15 aligned reads yields sequence accuracy of over 99% [25]. However, for GC-rich sequences we would expect that a higher number of aligned reads would be necessary to achieve the 99% reliability threshold since error rate of PacBio reads might be more elevated. Because of the under-representation of at least a part of GC rich sequences in PacBio datasets, a 15-pass coverage is probably insufficient and difficult to achieve. This will likely lead to gene models with lower accuracy rate. Such models would however still be useful in order to examine which sequence features are associated with sequencing and assembly difficulties.

### The RJF leptin gene

The sequence of the RJF leptin gene was used to develop an approach to reconstruct GC-rich gene models from PacBio reads, then to evaluate them using RT-PCR and RNA-seq studies, and finally to verify that the observations done with the RJF could be generalized to most avian species.

#### Definition of a gene model with Illumina and PacBio reads

**Fig. 3:**
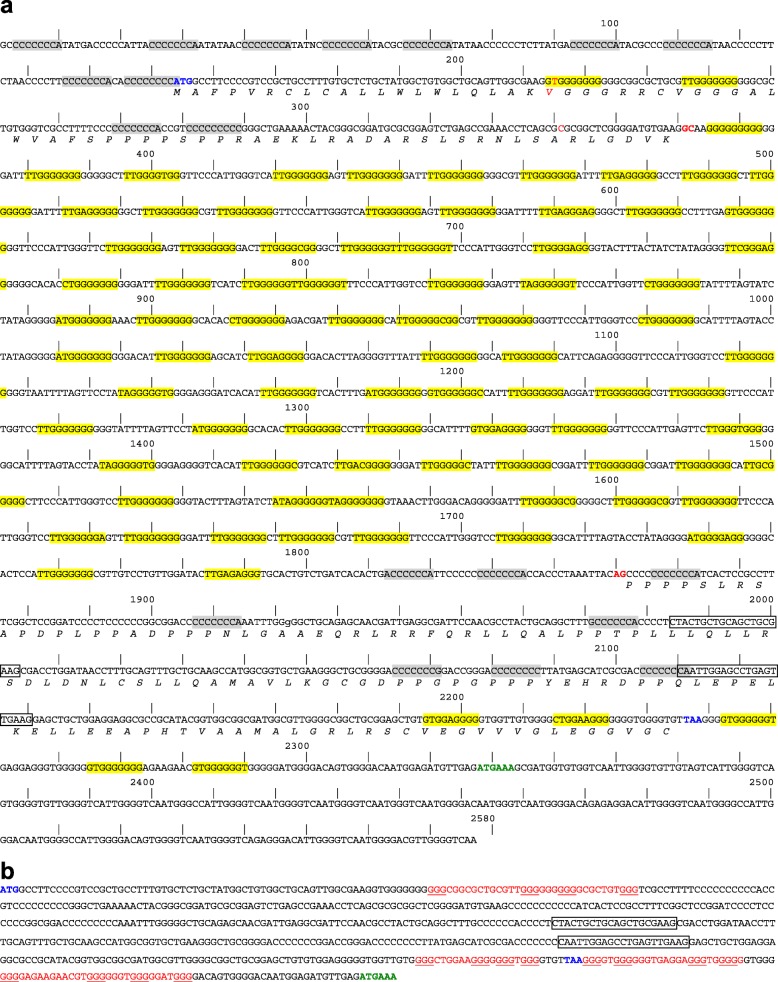
Nucleic acid sequence models of (**a**) region containing the coding exons split by a 1533 bp intron in the RJF leptin gene and (**b**) tcoding region within its mRNA. The start and stop codons, the dinucleotides at the RNA splicing site and the putative polyadenylation signal are shown in bold and indicated in blue, red and green respectively. Annealing sites for primers designed for PCR and RT-PCR are boxed. In (**a**), the protein sequence of the RJF leptin is shown in italics below the nucleic acid sequence of the coding regions. Complementary “CCCCCCCan” and “ttGGGGGGG” motifs are indicated in grey and yellow respectively. Single nucleotide polymorphisms with KT970642 sequence are indicated in red. In (**b**), motifs matching with the consensus of G4 structures (G_3_ + _N1-12_G_3_ + N_1-12_G_3_ + N_1-12_G_3_) are underlined and involved guanine stretches are shown in red

The KT970642 sequence model of the RJF leptin gene was used for searching two Illumina libraries composed of 600 bp genomic fragments of the RJF (each with a genome coverage ~10X). We extracted 4 reads from the region overlapping the first coding exons and 14 reads overlapping the second exon. These Illumina reads, along with those previously described [11] were aligned using MUSCLE [39] to improve the KT970642 model and to create a new gene model with extended sequences at both model extremities. This second model was used to search the two PacBio datasets described above using blastn and blasr. We extracted 11 PacBio reads from each dataset that fully or partly overlapped the sequence model and displayed sequence identities ranging from 70 to 87% with the model. These reads were oriented and aligned with MUSCLE. Sequence alignments were checked manually before adding aligned Illumina sequences (Additional file 4). This resulted in a 2577 bp genomic sequence model that completely overlapped both coding exons (Fig. 3a), and in a cDNA sequence model that included both coding exons and 95 nucleotides downstream of the stop codon that extended into a putative polyadenylation signal (Fig. 3b). The final genomic model was used to search the PacBio datasets a second time but no new reads with sequence identity of 70% or above were found. This suggests that the sequence accuracy of PacBio reads was limited on such a GC rich sequence (64.4% GC for the genomic model and 68.5% for the cDNA model). We estimated that our model was 15 to 20-fold under-represented in PacBio reads. Whatever the enzymatic procedure used, the intron and the 5′ and 3′ regions of this model prevented their amplification by PCR from genomic DNA (gDNA) for verification by Sanger sequencing.

Analysis of the RJF leptin gene model revealed that it has sequence characteristics not found for this gene in other vertebrate species. First, the intron between the two coding exons was longer than in other avian species (1497-bp in the RJF, while the largest previously described such intron was only 900 bp in *Melopsittacus undulatus*, accession KJ196275.1). Second, this gene had splice sites CG/AG that were atypical, but not unheard of [40]. Third, this gene contains two types of GC rich repeated motifs that are complementary in sequence and not located in the same genomic regions. The “CCCCCCCAN” consensus motif spans the exonic regions while the “TTGGGGGGG” consensus motif is concentrated on the exon separating both coding exons and in the 3′ region of the second coding exon. Interestingly, the sequence of these motifs were reverse complements and similar to the (TTAGGG)_n_ telomeric repeats [41]. This suggests they may be related to the subtelomeric location of this gene in chromosome 1 [4]. Finally, numerous stretches of guanines in the sequence of the genomic and cDNA models (Fig. 3b) matched with the consensus of the G4 structure described above (G_3_ + _N1-12_G_3_ + N_1-12_G_3_ + N_1-12_G_3_) where N is any base including G [28, 42]. This feature is important because these structures are known to prevent or diminish the capacity of DNA and RNA polymerases and reverse transcriptases to replicate nucleic acids [26, 43–45]. Similarly, the complementarity of “CCCCCCCAN” and “TTGGGGGGG” repeated motifs in the putative 5′ and 3′ untranslated regions and in exons suggested that the leptin mRNA were able to assemble complex intrastrand secondary structures that might be very stable because of their elevated GC content.

#### Impact of the choice of replication enzymes used in assays

**Fig. 4:**
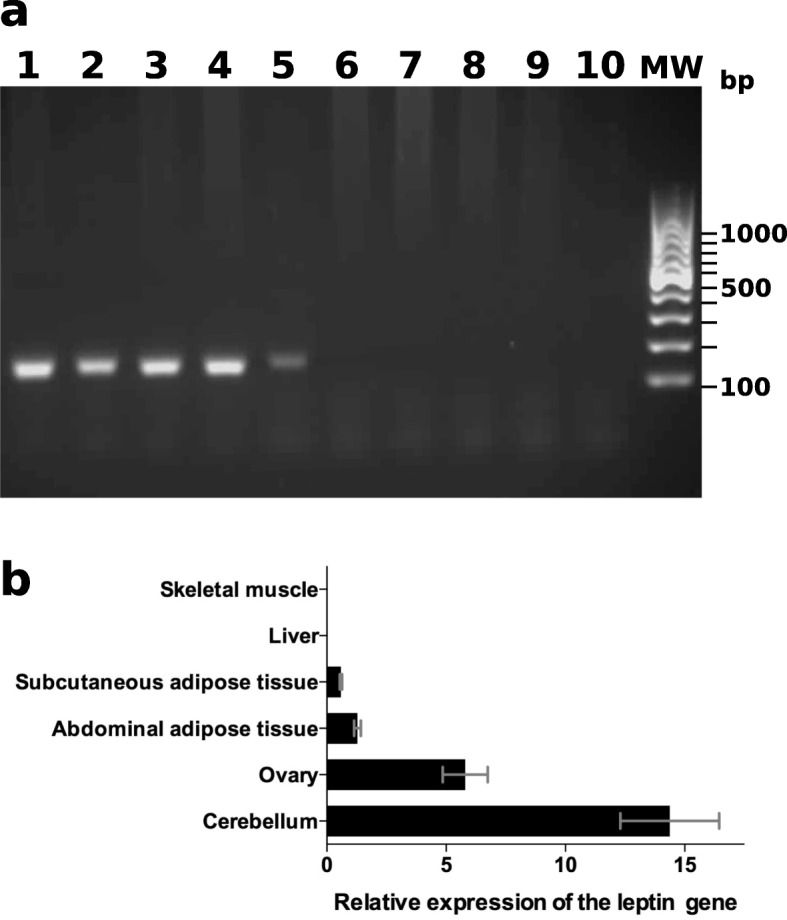
Products obtained with PCR and RT-qPCR assays targeted on the chicken leptin gene. In (**a**), amplification products obtained using a PCR assay on the chicken leptin gene. Genomic DNA samples were purified from a blood samples of single females belonging to the RJF species (1), the alsacian old French chicken line (2), the Araucana chicken line (3), a white leghorn chicken line (4), the *Gallus sonneratii* species (5), the quail species *Coturnix japonica* (6), the turkey species *Meleagris gallopavo* (7), the pekin duck species *Anas platyrhynchos domesticus* (8) and the duck of barbary species *Cairina moschata* (9). Lane 10 shows a control sample without gDNA. Lane MW shows a 100-bp ladder with molecular sizes in base pairs indicated on the right. In (**b**), RT-qPCR results indicating the relative expression of the leptin gene

In order to verify whether motifs capable of assembling into secondary structures in the leptin gene could alter its expression using RT-qPCR we developed two PCR assays. The first assay amplified an inner fragment of the second coding exons using RJF gDNA. It used several primer pairs including those previously published [11], and two thermostable DNA polymerases. The first polymerase was the routine GoTaq® (Promega), the second an enzyme designed to be efficient in GC-rich regions, the Taq KAPA HiFi (Kapa Biosystems). An amplification product with a suitable size and sequence was only obtained with the primer pair CTACTGCTGCAGCTGCGAAG and CTTCAACTCAGGCTCCAATTG and the GoTaq® enzyme. The specificity of this PCR assays was tested using gDNA samples from chicken lines and related or domesticated species (Fig. 4a). Results showed that the inner fragment of the leptin exon 2 could only be amplified from animals in the *Gallus* genus. In the second assay, three reverse transcriptases (RT) were tested for their ability to synthesize suitable cDNA for our PCR assay. The first RT was the classic Moloney Murine Leukemia Virus (M-MLV) RT (Promega), while the other two RT were enzymes engineered to deconstruct the intra-strand structures in messenger RNA (mRNA) molecules, the Opti M-MLV RT (Eurobio) and the SuperScript IV RT (Invitrogen). Whatever the priming strategy used (oligo-dT, hexanucleotides or both), only the engineered RTs achieved successful reverse transcription of PCR (RT-PCR) products. Because only a very faint band was obtained on agarose gel with the SuperScript IV RT, the Opti M-MLV RT was thereafter used to follow RNA expression of the leptin gene in various tissue samples. This included abdominal fat where the leptin gene was recently found to be absent from, based on RNA-seq data [21]. cDNA synthesized using the Opti M-MLV RT (Eurobio) showed that there was strong leptin expression in the hen cerebellum and in the ovary as previously shown [11], but also in abdominal adipose and subcutaneous adipose tissues (Fig. 4b). These results confirmed that the efficiency of cDNA synthesis widely depends on the replication ability the RT.

Following these findings, we searched PacBio RNA-seq projects (PRJEB13246, PRJEB13248 and PRJEB12891 [46]) in order to locate leptin sequences. These datasets were partly produced from brain mRNA, including those of the cerebellum that are appropriate for locating leptin transcripts. Our searches were in vain, probably because these datasets were obtained from cDNA synthesized with a classical M-MLV RT that is unable to synthesize through stable secondary intrastrand structures in RNA molecules.

The efficiency of RT-PCR amplifications of the leptin gene did not seem as high as for other genes such as the glyceraldehyde 3-phosphate dehydrogenase (GAPDH), likely because of the elevated GC content in the leptin gene. Therefore, our quantitative reverse transcription PCR (RT-qPCR) assay can be used to compare leptin mRNA amounts between RNA samples, but is not suitable for comparing leptin mRNA amounts with those of other genes with a similar or lower GC content.

#### Genomic features of leptin genes in other avian species

We investigated whether the unusual sequence features of the RJF leptin gene (GC content, presence of direct and inverted repeats, and motifs susceptible to assemble intrastrand G4 structures) were conserved among bird genomes. These features are of particular interest because they may limit the ability of researchers to sequence and annotate some bird genes.

We searched avian genomes in whole-genome shotgun contigs databases using tblastn at NCBI. In some species the leptin gene was found successfully assembled into contigs from Illumina reads. However, in other species, such as the mallard duck or the Japanese quail, no hits were found. Phylogenetically birds differentiated into two main lineages during evolution, the Palaeognathae and the Neognathae. Our investigation revealed that the genomic copies of avian leptin genes were available in databases only for species belonging to the Neognathae lineage, such as the zebra finch, the wavy parakeet, the collared flycatcher, the bald eagle and the golden eagle and the double-crested cormorant (Additional file 5a and f). The gene sequences from all of these species displayed an average GC content 8–10% above that of the RJF, had introns ranging from 400 to 700 bp in length with GT/AG splice sites (except in the double-crested cormorant that displayed a CG/AG splice site) and (G_3_ + _N1-12_G_3_ + N_1-12_G_3_ + N_1-12_G_3_) motifs and direct repeats.

In order to further our understanding of leptin genes in both bird lineages, we enlarged our searches to raw sequences. We first searched in our own Illumina datasets for two neognathae species, the African fisher eagle and the black-chinned hummingbird, and one palaeognathae species, the ostrich. In addition, we used public Illumina datasets for five palaeognathae species, the white-throated tinamou, the brown kiwi, the mallard duck, the muscovy duck and the Japanese quail. We successfully reconstructed the leptin gene of the African fisher eagle (Additional file 5 g) and obtained a partial sequence for the bird with the smallest known genome size, the black-chinned hummingbird (Additional file 5 h [47]). These genomic copies displayed similar sequence features to those of other neognathae species (GC content, presence of potential G4 motifs), but the GC content of the black-chinned hummingbird was the most elevated (82.49%). We also reconstructed the leptin gene of the ostrich (Additional file 5i), that of the muscovy duck (Additional file 5j) with the inner region of the intron lacking, and two partial sequences of the white-throated tinamou and the brown kiwi (Additional file 5 k and l). In these four palaeognathae species and in the RJF, we did not find any feature difference with those of neognathae, excepted that all the genes displayed a GC/AG splice site and a larger intron (800 to 1500 bp). However, we did not find Illumina or PacBio reads for the mallard duck or the Japanese quail. This suggested that the leptin gene sequence in these two species may be particularly difficult to isolate in Illumina and PacBio libraries and/or sequencing.

We concluded that palaeognathae leptin genes might be more difficult to assemble because they may be slightly larger due their intron size. This in turn means that they would likely contain more tandem repeats and motifs able to assemble into G4 structures which also might be more or less stable, depending on the biochemical environment [48].

### Sequence features of other orphan cDNAs from RJF

Because we were unable to find a solution to develop an automated alignment pipeline for GC-rich PacBio reads (even with a learning algorithm since the datasets for the learning are not available) we investigated orphan cDNAs manually for four cDNAs, chosen among Hron’s RJF cDNA (Hron et al. 2015) that were found not to be highly conserved in DNA sequence in other avian species [5, 20]. The four cDNA candidates had at least two exons and a gene size below 10–15 kbp in other non-avian species. Indeed, the size and the sequence features of introns of these GC-rich cDNA models is a factor limiting the reliability of such investigations.

#### TNFα gene

**Fig. 5:**
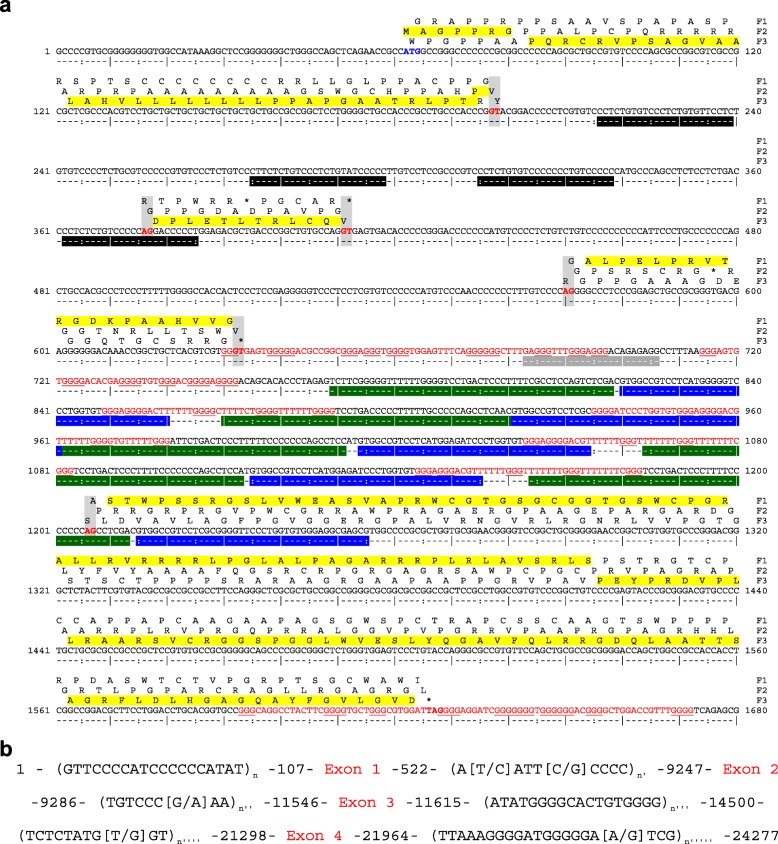
Sequence of the crow (*C. cornix cornix*) gene coding for TNFα (**b**) and organization of orthologous genes in the RJF (b). In (**a**), the crow sequence was extracted between positions 44,354 to 46,058 from the MVNZ01000346.1 contig [22], a sequence that was present in the original version of the *C. cornix cornix* genome model, but which was later split into several contigs in the second version. Above the DNA sequence is the translation into amino acids in all three frames of the plus strand. The crow TNFα sequence is shown in yellow. This sequence contained frame shifts in exons that resulted from errors in the assembly of the PacBio and Illumina reads. The start and stop codons are shown in bold and blue. Statistically significant blocks in direct and inverted repeats were identified with MEME at http://meme-suite.org/tools/meme and are indicated in black or dark grey boxes (depending on their orientation), green and blue boxes respectively. Motifs matching with the consensus of G4 structures (G_3_ + _N1-12_G_3_ + N_1-12_G_3_ + N_1-12_G_3_) are indicated in red. In (**b**) sequence features of the RJF gene are summarized, these were deduced from the alignment of PacBio reads (Additional file 5). The start and end positions of the PacBio read alignments for the four exons are indicated. Motifs in parentheses indicate tandem repeats, these are the only components of introns and the 5′ and 3′ regions. n, n’, n”, n”‘, n”“ and n”“‘describe different numbers of tandem repetition between motifs

TNFα is a cell signaling protein (cytokine) involved in systemic inflammation and is one of the cytokines that make up the acute phase reaction. The features of a TNFα gene recently described in the crow *Corvus cornix cornix* [22] were first investigated before to elucidate the organization of RJF TNFα gene. The gene in this species is short, 1680 bp in length, and has a GC content of 69.58%, lower than that of its coding exons (77.81%). This gene contains four direct repeats in its first intron (Fig. 5a, regions in black boxes) and one in the reverse orientation at the beginning of the third intron (Fig. 5a, region in dark grey box). This last intron also contains two other types of inserted tandem repeats (Fig. 5a, region in green and blue boxes). In this third intron and in the 3′ region, several G-rich motifs were found that are capable of assembling into G4 structures.

A model of the RJF TNFα gene was obtained by aligning 18 reads (Additional file 6) extracted from the two PacBio datasets described above using the MF000729.1 sequence [22] as a query in blastn and blasr searches. We calculated that the read coverage overlapping the query was at least 25 to 30 fold below that expected in files with a theoretical coverage of 180X and 60X respectively. The alignment was 24,277 bp long and included four coding exons, all regions covered by at least 5 reads. We did not find any Illumina reads overlapping exon-intron junctions in the chicken datasets described above. Because of low coverage and the large error rate (> 20%), only a consensus model of sequence organisation was setup for the RJF TNFα gene (Fig. 4b). Because approximately one third of positions in the alignment were used to manage mismatches and indels, we estimate that the RJF TNFα gene is approximately 14,400 bp long (i.e. about 10 fold longer than that of the crow). The TNFα gene is known to display 2 to 3-fold size variations among vertebrates (2769 bp in human, 5244 bp in the coelacanth, 2817 bp in *Xenopus tropicalis*, 6236 bp in the anole lizard and 2346 bp in the Chinese softshell turtle) but the RJF one is the longest described one. The RJF TNFα gene model also revealed that the 5′ and 3′ non-coding regions of this gene and its introns were filled with motifs repeated in tandem. Whatever the non-coding region, the sequence of these repeats allowed for the detection of several hundreds of G-rich motifs capable of assembling into G4 structures on the plus or the minus strand.

#### MRPL52 gene

**Fig. 6:**
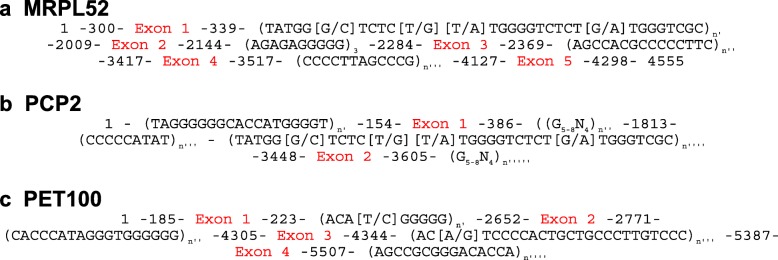
Sequence organization of MRPL52 (**a**), PCP2 (**b**) and PET100 (**c**) genes in the RJF. The start and end positions in the PacBio read alignment of four exons are indicated. Motifs in parentheses indicate tandem repeats, these are the only components of introns and the 5′ and 3′ regions. n, n’, n”, n”‘, n”“ and n”“‘describe different numbers of tandem repetition between motifs

MRPL52 (a.k.a. SLC7A7) is a component of the mitochondrial ribosome. A RJF model of this gene was obtained by aligning 12 reads (Additional file 7) extracted from the two PacBio datasets described above using its cDNA model as a query in blastn and blasr searches. The alignment was 3 kbp long and included five coding exons. Because of low coverage and low identity rates between PacBio reads (< 80%; due to sequencing errors), only a consensus model of sequence organisation was set-up for the RJF MRPL52 gene (Fig. 6a). The MRPL52 gene is known to display 2-fold size variations among vertebrates (5158 bp in human, 2847 bp in the mouse, and 5467 bp in the anole lizard). The RJF MRPL52 gene model also revealed that its introns were filled with motifs repeated in tandem that contained numerous G-rich motifs capable of assembling into G4 structures on the plus or the minus strand.

#### PCP2 gene

PCP2 is a member of the GoLoco domain-containing family, and is only found in cerebellar Purkinje cells and retinal ipolar cells in vertebrates. A model of the RJF PCP2 gene was obtained by aligning only 7 reads (Additional file 8) extracted from the two PacBio datasets as described above. The alignment of the PCP2 gene was 3451 bp long and included two coding exons. Because of low coverage and identity rate between reads (< 80%), only a consensus model of sequence organisation was established (Fig. 6b). PCP2 gene sizes do not display large variations among vertebrates (2137 bp in human, 2174 bp in mouse, and 2821 bp in the anole lizard). Therefore, its size in the RJF did not seem to be a distinguishing feature. However, it also displayed introns filled with motifs repeated in tandem that contained numerous G-rich motifs capable of assembling into G4 structures on the plus or the minus strand.

#### PET100 gene

PET100 is involved in mitochondrial Complex IV (cytochrome c oxidase) biogenesis. A model of the RJF PET100 gene was obtained by aligning 10 reads (Additional file 9) extracted from the two PacBio datasets as described above for TNFα. The alignment of the PET100 gene was 5321 bp long and included four coding exons. Because of low coverage and the large error rate (> 20%), only a consensus model of sequence organisation was set-up for the RJF PET100 gene (Fig. 6c). Although the P100 gene has few annotations in sauropsida species, its size does not seem to vary much (2219 in human, 4300 in mouse, and 2660 in zebrafish). Gene size was not a distinguishing feature. Similar to the other four genes, its introns displayed numerous motifs repeated in tandem that contained numerous G-rich motifs capable of assembling into G4 structures on the plus or the minus strand.

Together, these five case studies suggest that neither GC content nor gene size were likely the reason why these genes were difficult to sequence and assemble. Our hypothesis was that the presence of numerous tandem repeated motifs containing abundant G-rich motifs capable of assembling into G4 structures in introns (and in some cases within the CDS) had features that probably made them difficult to sequence and assemble using second generation sequencing technology.

## Discussion

The difficulty of sequencing and assembling missing regions of avian genome models has typically been interpreted as the result of two sequence characteristics, elevated GC contents and high rates of interspersed and tandem repeats [5, 49]. In an effort to address these issues Tilak et al. [50] suggested that Illumina libraries be made that were enriched in GC-rich sequences. Here, using 5 gene cDNA sequences, a complete set of 21 orphan cDNAs, and genomic copies of the SV2A and rDNA genes as queries we searched such enriched datasets (IDs: ERX2234588 to ERX2234598) from RJF gDNA fragments. However, we did not find any more reads with these enriched data sets than with standard libraries. This indicated that for these genes, GC content was not the main factor limiting their presence in Illumina gDNA libraries and/or sequencing. Furthermore, we would note that some avian cDNAs with higher GC content than either RJF leptin or TNFα genes have successfully been sequences and assembled using Illumina technology (Fig. 2).

Rather than just GC richness, we find that the presence of motifs capable of assembling into G4 structures has the most impact on the ability to sequence a region by Illumina technology (26–30). RJF leptin and TNFα gene size were larger than their orthologues in other vertebrate species, their non-coding regions rich were mostly composed of short tandem repeats, sequences ideal for preventing assembly and that were absent in non avian orthologs that were so far assembled. Furthermore, these non-coding regions were not represented among Illumina gDNA library reads. This suggests either that these reads were not integrated during library creation, or could not be sequenced using Illumina technology.

On the other hand, searching PacBio reads revealed this technology produced reads overlapping the RJF leptin, TNFα, MRPL52, PCP2 and PET100 genes. However, coverage of GC rich regions investigated here was lower than expected and reads displayed an error rate that was significantly higher than expected [33]. Therefore, PacBio technology allowed for sequencing of regions with a GC content > 60%, regions that were often fully absent from Illumina datasets. However, it did this less efficiently than for regions with a GC content < 60%. We hypothesize that this was due to the presence of G4 structures and dyadic intrastrand structures in GC-rich regions.

We provided genomic sequence models for five RJF genes, as well for the leptin genes of several avian species. Such genomic models, reconstructed from under-represented reads, should to be validated by PCR and sequencing. Unfortunately, and in spite of huge efforts, we could not get validation for the intronic sequences because suitable primers could not be designed because of their high GC content. We only succeeded in amplifying an inner fragment of the second coding exon from RJF gDNA (as described above) and from the pekin duck (using as forward and reverse primers, CAGCGGCTGCAGCTTTTC and CAACCGTCCCATGGCCAAA and PCR conditions similar to those used here for the RJF). The need to validate cDNA models by PCR is essential because trace reads originating from SRA files may be contaminated by outside sources. An example of such contamination is the 27 RNA-seq datasets of the Chickpress project (PREJB4677) that contained 20–30% human cDNAs. Here, we only used cDNA sequences as queries that had previously been validated [18, 22]. These five genes should, at a later point, have the sequence of their non-coding regions verified, once appropriate amplification and sequencing procedures become available. Nevertheless, the gene models presented here should be of great interest to the development of solutions aiming at deconstructing secondary intrastrand DNA structures to improve sequencing efficiency of Illumina and PacBio technologies. To our knowledge, these RJF gene models are the first public dataset of such sequences that are very reluctant to sequencing. These could be used for evaluating the efficiency of high throughput sequencing technologies*.*

## Conclusions

High GC content was not the principal reason why certain regions of avian genomes are missing from genome assembly models. Instead, it is the presence of tandem repeats containing motifs capable of assembling into very stable secondary structures that is likely responsible. To our knowledge, our work is the first study dealing with this issue in the context of avian genomics. Our results are also in agreement with the recent literature in this field about the reliability of the Pacbio Technology with regard to the sequencing of non-B genomic regions [33]. Because G-quadruplexes most likely explain the absence of some genes and regions in genome and transcript models (e.g. in Additional file 10), solutions to circumvent these problems and to obtain their sequence will likely be dependent sequencing technique innovations. Our study was also the first one to bring datasets of genomic sequences that could be useful to benchmark sequencing technique innovations dedicated to circumvent problems raised by very stable secondary structures.

## Methods

### Biological samples

All biological samples used for DNA extracts were females. Blood samples from chicken lines, araucana, alsacian and white leghorn, japanese quail, turkey, pekin duck and duck of barbary were obtained from breeds maintained at the INRA UE1295 PEAT experimental facilities (Pôle96 d’Expérimentation Animale de Tours, Agreement N° C37–175-1). Those from the RJF *Gallus gallus* and the grey jungle fowl *Gallus sonneratii* were supplied by Christophe Bec, Parc des oiseaux (Villars les Dombes 01330 France) et Christophe Auzou (Grand Champs 89,350 France). Those from the ostrich and African fisher eagle were supplied respectively by la Réserve de Beaumarchais (Autrèche 37,110 France) and Zooparc de Beauval (Saint Aignan 41,198 France). Biopsies of pectoral skeletal muscles from black-chinned hummingbird were supplied by the Department of Biology & Museum of Southwestern Biology of University of New Mexico (USA). For RNA extracts, tissues biopsies were supplied by Hendrix Genetics (Saint Laurent de la Plaine, France) and were from broiler breeder female chicks (Cobb 500), 35 weeks of age.

### Nucleic acid purifications

gDNA samples were prepared from 100 μL of fresh red blood cells using the Nucleospin Tissue kit (Macherey-Nagel). Total RNA was extracted from abdominal adipose tissue, subcutaneous adipose tissue, liver and Pecto skeletal muscle from 35 weeks-old hens by homogenization in TRIzol reagent using an Ultraturax, according to the manufacturer’s recommendations (Invitrogen by Life Technologies, Villebon sur Yvette, France). The quality and concentration of nucleic acid samples were evaluated using a NanoDrop™ 2000 spectrophotometer.

### Illumina sequencing of RJF genome

Library construction and sequencing were performed at the Plateforme de Séquençage Haut Débit I2BC (Gif-sur-Yvette 91,198 France). Illumina libraries of 600 bp genomic fragments were prepared without PCR amplification, as recommended, to optimize for the presence GC and AT-rich sequences (Aird et al. 2011, Oyola et al. 2012). Paired-end sequencing with reads of 75 and 250 nucleotides in length were performed with at least 10X coverage. All raw and processed data are available through the European Nucleotide Archive under accession numbers PRJEB22479 and PRJEB25675 for the RJF, PRJEB24169 for the ostrich, PRJEB27669 for the african fisher eagle and PRJEB27670, for the back-chinned hummingbird.

### Searching databases

Files containing the 2323 cDNA models were downloaded from 10.6084/m9.figshare.5202853 [5, 20]. 2132 of these were validated [5, 20]. Their functional annotation must be carefully reviewed (Additional file 3a.1), among these a few errors were found. For example, cDNA models in file aves_ENSPSIG00000014155_Hmm5_gapClean40.fasta was annotated as coding piwiL4 while it should have been piwiL1; data corresponding to orthologues of the ENSPSIG00000011968 *Pelodicus* sequence and describing cDNA coding the SLC2A4/GLUT4 protein were not released by the authors. The galGal5 and galGal6 genome sequences and coding sequences were downloaded from ftp://ftp.ncbi.nlm.nih.gov/genomes/Gallus_gallus. Release 94 and 96 of the Ensembl chiken CDS were downloaded from ftp://ftp.ensembl.org/pub/release-94/fasta/gallus_gallus/ and ftp://ftp.ensembl.org/pub/release-96/fasta/gallus_gallus/. Searches with cDNA models as queries for galGal5 and galGal6 CDSs available at NCBI Ensembl release 94 & 96. These were done using blastn within the script blastfasta.pl as described [51]. Searches for polyexonic genes using cDNA models as queries in the galGal5 and galGal6 genome models were done using blastn and HSPs were fused using agregfilter.pl as described [51]. Searches for polyexonic genes using cDNA models as queries in Gallus WGS and TSA datasets were done using the “Remote BLAST” plugin at NCBI as recommended (https://www.ncbi.nlm.nih.gov/books/NBK279668/). Hits were defined as positive when more than 90% of the query was aligned with the subject sequence with an identity above 95%. Such a rate of identity was used because numerous cDNA models contained long stretches of Ns.

To reconstruct leptin cDNAs in five palaeognathae species, we used public Illumina datasets for the white-throated tinamou (dataset ID: SRR952232 to SRR952238), the brown kiwi (dataset ID: ERR519283 to ERR519288 and ERR522063 to ERR5220668), the mallard duck (dataset ID: SRR7194749 to SRR7194798), the muscovy duck (dataset ID: SRR6300650 to SRR6300675 and SRR6305144) and the Japanese quail (24 Illumina datasets of PRJNA292031 plus about 20 Gbp of PacBio reads kindly supplied by Dr. J. Gros (Pasteur Institute, Paris, France).

### Construction of gene models from Pacbio reads

Files containing Illumina and PacBio reads from RJF available from public databases were downloaded using the sra toolkit (downloaded at https://www.ncbi.nlm.nih.gov/sra/docs/toolkitsoft/) to produce fasta formatted files. Presence of contamination in Illumina reads was tested for each file by aligning it to the genomes of the most commonly studied genetic models (*Escherichia coli*, *Saccharomyces cerevisiae*, *Drosophila melanogaster*, *Danio rerio*, *Mus musculus* and *Homo sapiens*) using HISAT2 or bowtie2, depending on the mRNA or the genomic origin of the nucleic acids. Below 2.5% of aligned reads per file or alignment, we considered that there was no significant contamination by these species. Illumina reads were thereafter searched using blastn. PacBio reads were searched using blastn and blasr [52] and produced similar results once parameters were optimized (blastn command line: blastn -db [name of the database for blast] -query [query.fa] -out [name of the file containing results] -evalue 100 -task blastn -word_size 5 -dust no -num_threads 1; blasr command line: blasr [PacBio reads file.fa] [query.fa] --header --minReadLength 400 --minAlnLength 400 --bestn 100 --out Resoutput -m 0 --nproc 1). Reads were extracted and oriented before alignment with MUSCLE for Illumina reads. Pacbio reads were aligned one by one using the --add option of mafft and as an alignment seed one cDNA model previously aligned with Illumina reads (as for the leptin gene) or one cDNA model manually aligned with a Pacbio reads, generally the best hit in blasr and blastn results was used. The alignment of each Pacbio read aligned with mafft was verified and adjusted manually in order to properly align poorly sequenced regions as well as to identify polypurine or polypyrimidine tracts insertions that are sporadically found in sequenced GC-rich reads. The five alignments produced here can be visualized using Aliview (https://github.com/AliView/AliView) or Seaview (http://www.seaviewfishing.com/DownloadSoftware.html) freeware packages. Additional files 4 and 6, 7, 8, 9 are in fasta format and contain the alignment of all Illumina and/or PacBio reads used to calculate the genomic models of the RJF leptin, TNFα, MRPL52, PCP2 and PET100 genes. In Additional file 4, the 10 sequences at the bottom of the alignment correspond to the Seroussi’s et al. (2016) partial cDNA model and the 9 reads used to calculate it. Sequence names of each read are indicated. Label “strand (+)” or “ strand (-)” at the end of the name indicated that the read was aligned in the forward or reverse-complement orientation. Sequence sections that were impossible to align were represented as N tracts. Features of orthologous genes in vertebrates were investigated using genomicus 94.01 at http://www.genomicus.biologie.ens.fr/genomicus-94.01/cgi-bin/search.pl.

### Detection of non-B DNA G4 motifs

To detect candidate motifs putatively able to assemble G4 and other non-B DNA motifs, we used facilities available at https://nonb-abcc.ncifcrf.gov/apps/site/default [35]. The detection mode of the G4 candidates is done by text mining for the detection of a strict motif G_3_ + _N1-12_G_3_ + N_1-12_G_3_ + N_1-12_G_3_. Because this motif did not stand any sequence degeneracy when a motif is detected, its detection probability was 100%.

### PCR on gDNA

Our optimal procedure for PCR amplification was performed on 60 ng of avian gDNA in 10 mM Tris-HCl, pH 9, 4 mM MgCl2, 50 mM KCl, 0.1% TritonX100, 150 μM of each dNTP, and 0.1 mM of each oligonucleotide in a 50 μl reaction volume with 1 unit GoTaq DNA Polymerase (Promega). Each PCR was carried out in a programmable temperature controller (Eppendorf) for 30 cycles. After initial denaturation (5 min at 98 °C), the cycle was as follows: denaturing at 98 °C for 20″, annealing at 60 °C for 15″, and extension at 72 °C for 1′. At the end of the 30th cycle, the heat denaturing step was omitted, and extension was allowed to proceed at 72 °C for 5′. Each amplified sample could then be purified using a QIAquick PCR purification kit (Qiagen) and sent to Eurofins Genomics for a Sanger sequencing in order to verify its leptin origin.

### RT-PCR assay

The classic way we used to generate cDNA was by reverse transcription (RT) of total RNA (1 μg) in a mixture comprising 0.5 mM of each deoxyribonucleotide triphosphate (dATP, dGTP, dCTP and DTTP), 2 M of RT buffer, 15 μg/μL of oligodT, 0.125 U of ribonuclease inhibitor, and 0.05 U M-MLV RT for one hour at 37 °C. Our optimal procedure to synthesize cDNA was performed from 0.1 μg total RNA using a mix of oligodT and hexanucleotides as primers and the Opti M-MLV RT under conditions recommended by the supplier (Eurobio). Real-time PCR was performed using the MyiQ Cycle device (Bio-Rad, Marnes-la-Coquette, France), in a mixture containing SYBR Green Supermix 1X reagent (Bio-Rad, Marnes la Coquette, France), 250 nM specific primers (Invitrogen by Life Technologies, Villebon sur Yvette, France) and 5 μL of cDNA (diluted five-fold) for a total volume of 20 μL. Samples were duplicated on the same plate and the following PCR procedure used: after an incubation of 2 min at 50 °C and a denaturation step of 10 min at 95 °C, samples were subjected to 40 cycles (30 s at 95 °C, 30 s at 60 °C and 30 s at 72 °C). The levels of expression of messenger RNA were standardized to the GAPDH reference gene. For the leptin gene, the relative abundance of transcription was determined by the calculation of e^-ct^. Relative expression of the gene of interest was then related to the relative expression of the geometric mean of the GAPDH reference gene.

## Supplementary information

**Additional file 1.** Sequences of one avian mRNA (a) and exons of one avian gene (b) coding for an intellectin-like protein (ITLN1), and their amino acid sequence comparison (c).

**Additional file 2.** The sequence of RJF genes coding for the carnitine O-palmitoyltransferase 1 (CPT1C) and the insulin-like peptide 5 (ISNL5).

**Additional file 3.** cDNA models described in [5, 20].

**Additional file 4.** Alignment of Illumina and Pacbio reads used to define the genomic sequence of the RJF leptin gene.

**Additional file 5.** Genomic sequences of the avian leptin gene

**Additional file 6.** Alignment of Pacbio reads used to define the genomic sequence of the RJF TNFαgene.

**Additional file 7.** Alignment of Pacbio reads used to define the genomic sequence of the RJF MRPL52gene.

**Additional file 8.** Alignment of Pacbio reads used to define the genomic sequence of the RJF TNF gene.

**Additional file 9.** Alignment of Pacbio reads used to define the genomic sequence of the RJF PET100gene.

**Additional file 10.** Location of G-quadruplex structures in the 14 cDNA models described in [19].

## Data Availability

The Illumina raw reads generated and analysed during the current study have been submitted to the European Nucleotide Archive (https://www.ebi.ac.uk/ena/submit/sra/#home) and are available under accession numbers PRJEB22479, PRJEB24169, PRJEB25675, PRJEB27669 and PRJEB27670. Public datasets are available in the NCBI repository (https://www.ncbi.nlm.nih.gov/sra/) and their accession numbers were cited in the main text.

## Abbreviations

bp: base pair
cDNA: complementary DNA
CDS: Coding sequence
CPT1C: Carnitine O-palmitoyltransferase 1
DNA: Deoxyribonucleic acid
G4: G-quadruplex
GAPDH: Glyceraldehyde 3-phosphate dehydrogenase
gDNA: Genomic DNA
ITLN1: Intellectin 1
M-MLV: Moloney murine leukemia virus
mRNA: messenger RNA
MRPL52: Mitochondrial ribosomal protein L52
ORF: Open reading frame
PCP2: Purkinje cell protein 2,
PCR: Polymerase chain reaction
PET100: Protein homolog to yeast mitochondrial PET100
rDNA: Region coding the 18S–5.8S-28S ribosomal RNA
RLN3: Relaxin 3
RNA: Ribonucleic acid
RNA-seq: RNA sequencing
rRNA: 18S–5.8S-28S ribosomal RNA
RT: Reverse transcription/transcriptase
RT-PCR: Reverse transcription PCR
RT-qPCR: Quantitative reverse transcription PCR
SV2A: Synaptic vesicle glycoprotein 2A
TNFa: Tumor necrosis factor α
