# Supplementary material for: Sequence properties of certain GC rich avian genes, their origins and absence from genome assemblies: case studies
Source: BMC Genomics. 2019 Oct 14;20:734. doi: 10.1186/s12864-019-6131-1 (PMC6792250; doi:10.1186/s12864-019-6131-1)

**Additional data 1**: **Sequences of one avian mRNA (a) and exons of one avian gene (b) coding for an intellectin-like protein (ITLN1), and their amino acid sequence comparison (c).**

**a.** **Amino acid sequence of an ITLN1 protein encoded by a mRNA in *Tinamus guttatus* (DNA sequence ID: XM_010213600; protein sequence ID: XP_010211902.1).** In the peptide sequence, the five exons known in the reptilian sequence of *Anolis carolensis* (sequence ID: XM_010213600) appear in blue or in black. The N-terminal residues are in red and are absent in other sauropsidian and vertebrate proteins. The methionine is in bold and is likely the translation start site in avian species

MGRTGEQGEKSCCH**M**STAGVGWTLVASVHENNAHGKCTVGDRWSSQQGSSPLWPEGDGAWADHSTFGSAVGATADDYKNPGYYDLGARDVAVWHVPNRTPMAEWQSQALLRYRTHNGFLAEEGGNLLRLFQKYPVKYGAGQCLNDNGPAVPVIYDFGSPEVASRYYSPNGQKEFVPGFLQFRVFNNERAAMALCPGMKVTGCNTEHHCIGGGGYFPESSPQQCGDFPGYVWNGYGTHTAWSTSKEILEAAVLLFYR

**b. Sequence of the five exons coding an ITLN1 protein in *Apteryx australis mantelli* (sequence ID: XM_010213600).** The *A. australis mantelli* contigs and sequences available in public databases were searched with tblastn using XP_010211902.1 as a query. Five contigs (sequence ID: LK321580.1, LK321580.1, LK339259.1, LK213064.1, LK259219.1) were found, each of them overlapping one exon. Further searching to find Illumina reads, contigs or scaffolds overlapping introns were unsuccessful. We observed that the available sequences were 65% GC-rich. The non availability of Illumina reads in datasets (ID: ERR522063 to ERR522068 and ERR519283 to ERR519288) suggested that the GC content in non-coding introns might be more elevated and would hamper their ability to be integrated into genomic libraries and/or sequencing. Above each nucleotide the sequence translation in all three frames is shown. The coding frame in each exons is highlighted in yellow. Splice sites at both intron ends are highlighted in grey and shown in red. No ITLN1 gene was found in galGal6.

A H R R R R D L P D L L R H E H G G G R L D A G G Q R A R E Q R P R Q V H R R R F1

R T A D G E T Y Q T F C D M S T A G G G W T L V A S V H E N N A H G K C T A G D F2

A P P T A R P T R P S A T * A R R G A A G R W W P A C T R T T P T A S A P P A T F3

1 GCGCACCGCCGACGGCGAGACCTACCAGACCTTCTGCGACATGAGCACGGCGGGGGGCGGCTGGACGCTGGTGGCCAGCGTGCACGAGAACAACGCCCACGGCAAGTGCACCGCCGGCGA 120

----:----|----:----|----:----|----:----|----:----|----:----|----:----|----:----|----:----|----:----|----:----|----:----|

P L V Q P A G Q Q P S L A R G R R Q L G Q Q L H L R L R R G R H Q R R L X X X X F1

R W S S Q Q G S S P L W P E G D G S W A N N C T F G S A V G A T S D D Y X X X X F2

A G P A S R A A A L S G P R A T A A G P T T A P S A P P W A P P A T T T X X X X F3

121 CCGCTGGTCCAGCCAGCAGGGCAGCAGCCCTCTCTGGCCCGAGGGCGACGGCAGCTGGGCCAACAACTGCACCTTCGGCTCCGCCGTGGGCGCCACCAGCGACGACTACAnnnnnnnnnn 240

----:----|----:----|----:----|----:----|----:----|----:----|----:----|----:----|----:----|----:----|----:----|----:----|

X X C R P P A E P G V L R P Q R Q G P V R V A R A Q P G A P G R V A P A G P A A F1

X A A A L P Q N P G Y Y D L S A R D L S V W H V P N R A P L A E W P Q R A L L R F2

X L P P S R R T R G T T T S A P G T C P C G T C P T G R P W P S G P S G P C C A F3

241 nnnnGCTGCCGCCCTCCCGCAGAACCCGGGGTACTACGACCTCAGCGCCAGGGACCTGTCCGTGTGGCACGTGCCCAACCGGGCGCCCCTGGCCGAGTGGCCCCAGCGGGCCCTGCTGCG 360

----:----|----:----|----:----|----:----|----:----|----:----|----:----|----:----|----:----|----:----|----:----|----:----|

L P H P E R L P R H R G R Q P A P P L P G T G A R P S P A P V P P H A K P L W C F1

Y R T Q S G F L A T E G G N L L R L Y Q V P A P G P A P H R C L P M P S L C G A F2

T A P R A A S S P P R A A T C S A S T R Y R R P A Q P R T G A S P C Q A S V V P F3

361 CTACCGCACCCAGAGCGGCTTCCTCGCCACCGAGGGCGGCAACCTGCTCCGCCTCTACCAGGTACCGGCGCCCGGCCCAGCCCCGCACCGGTGCCTCCCCATGCCAAGCCTCTGTGGTGC 480

----:----|----:----|----:----|----:----|----:----|----:----|----:----|----:----|----:----|----:----|----:----|----:----

P Q A A P A P L P T P G I X X X X X Y G A G Q C P S D N G P A V P V V Y D V G S F1

R R P L R R P C P H P A S X X X X X T A P G S A R P T T A P P C P S S T T S A R F2

A G R S G A P A H T R H X X X X X V R R R A V P V R Q R P R R A R R L R R R L A F3

481 CCGCAGGCCGCTCCGGCGCCCCTGCCCACACCCGGCATCnnnnnnnnnnnnnnGTACGGCGCCGGGCAGTGCCCGTCCGACAACGGCCCCGCCGTGCCCGTCGTCTACGACGTCGGCTCG 600

----:----|----:----|----:----|----:----|----:----|----:----|----:----|----:----|----:----|----:----|----:----|----:----|

P E V A S R Y Y S P Y G Q S E C A P V G R X X X X X P G E F I P G F L Q F R V F F1

P R S P P D T T R P T G K V S A R R W G X X X X X R Q G S S S P A S S S S A S S F2

R G R L Q I L L A L R A K * V R A G G A X X X X X A R G V H P R L P P V P R L Q F3

601 CCCGAGGTCGCCTCCAGATACTACTCGCCCTACGGGCAAAGTGAGTGCGCGCCGGTGGGGCGnnnnnnnnnnnnnnCGCCAGGGGAGTTCATCCCCGGCTTCCTCCAGTTCCGCGTCTTC 720

----:----|----:----|----:----|----:----|----:----|----:----|----:----|----:----|----:----|----:----|----:----|----:----|

N N E K A A M A L C P G M K V T G C N T E H V S T R W A G A R R R G A V S X X X F1

T T R R R R W L C A P A * K S P A A T P S T * A R G G P V R G G A A Q S V X X X F2

Q R E G G D G S V P R H E S H R L Q H R A R E H A V G R C A A A R R S Q X X X X F3

721 AACAACGAGAAGGCGGCGATGGCTCTGTGCCCCGGCATGAAAGTCACCGGCTGCAACACCGAGCACGTGAGCACGCGGTGGGCCGGTGCGCGGCGGCGCGGCGCAGTCAGTGnnnnnnnn 840

----:----|----:----|----:----|----:----|----:----|----:----|----:----|----:----|----:----|----:----|----:----|----:----|

X X G S G R A G G A L G A R E P S A H A A A C W Q E L P R W G Q A P S H T T G V F1

X X A R D G L A G H W E H G S H Q P T L L L A G R S C R D G A K L P A T Q L G S F2

X G L G T G W R G I G S T G A I S P R C C L L A G A A A M G P S S Q P H N W G R F3

841 nnnnnGGGCTCGGGACGGGCTGGCGGGGCATTGGGAGCACGGGAGCCATCAGCCCACGCTGCTGCTTGCTGGCAGGAGCTGCCGCGATGGGGCCAAGCTCCCAGCCACACAACTGGGGTC 960

----:----|----:----|----:----|----:----|----:----|----:----|----:----|----:----|----:----|----:----|----:----|----:----|

G H S C S S G G G C R R L V P A H G G G T Q E G G G Q L P S S P R S P P G G L L F1

G T A A H P V A D A A V W S Q R M E V A R R K E G D S S P H P P A A L L E G Y C F2

A Q L L I R W R M P P S G P S A W R W H A G R R G T A P L I P P Q P S W R V I A F3

961 GGGCACAGCTGCTCATCCGGTGGCGGATGCCGCCGTCTGGTCCCAGCGCATGGAGGTGGCACGCAGGAAGGAGGGGGACAGCTCCCCTCATCCCCCCGCAGCCCTCCTGGAGGGTTATTG 1080

----:----|----:----|----:----|----:----|----:----|----:----|----:----|----:----|----:----|----:----|----:----|----:----|

L N D P K L E G C W C C W E M S P S C P L G W G E P R S H A A M A P H R S K K G F1

* M T P N W R D A G V A G K C P R A V P L D G E S H G R M L P W L H T A A R R E F2

E * P Q T G G M L V L L G N V P E L S P W M G R A T V A C C H G S T P Q Q E G N F3

1081 CTGAATGACCCCAAACTGGAGGGATGCTGGTGTTGCTGGGAAATGTCCCCGAGCTGTCCCCTTGGATGGGGAGAGCCACGGTCGCATGCTGCCATGGCTCCACACCGCAGCAAGAAGGGA 1200

----:----|----:----|----:----|----:----|----:----|----:----|----:----|----:----|----:----|----:----|----:----|----:----|

T G A P G V I F Q A D P C A A G G M G Q V G Q R P C X X X X X X X X X X X X X X F1

R G H L G * F S R L T H V Q Q V G W G R W D R G P V X X X X X X X X X X X X X X F2

G G T W G D F P G * P M C S R W D G A G G T E A L X X X X X X X X X X X X X X X F3

1201 ACGGGGGCACCTGGGGTGATTTTCCAGGCTGACCCATGTGCAGCAGGTGGGATGGGGCAGGTGGGACAGAGGCCCTGTNNNNNNNNNNNNNNNNNNNNNNNNNNNNNNNNNNNNNNNNNN 1320

----:----|----:----|----:----|----:----|----:----|----:----|----:----|----:----|----:----|----:----|----:----|----:----|

X X X X X X X X X X X X X X X X X X X X X X X X X X X X X X X X X X X X X X X X F1

X X X X X X X X X X X X X X X X X X X X X X X X X X X X X X X X X X X X X X X X F2

X X X X X X X X X X X X X X X X X X X X X X X X X X X X X X X X X X X X X X X X F3

1321 NNNNNNNNNNNNNNNNNNNNNNNNNNNNNNNNNNNNNNNNNNNNNNNNNNNNNNNNNNNNNNNNNNNNNNNNNNNNNNNNNNNNNNNNNNNNNNNNNNNNNNNNNNNNNNNNNNNNNNNN 1440

----:----|----:----|----:----|----:----|----:----|----:----|----:----|----:----|----:----|----:----|----:----|----:----|

X X X X X X X X X X X X X X X X X X X X X X X X X X X X X X X X X X X X X X X X F1

X X X X X X X X X X X X X X X X X X X X X X X X X X X X X X X X X X X X X X X X F2

X X X X X X X X X X X X X X X X X X X X X X X X X X X X X X X X X X X X X X X X F3

1441 NNNNNNNNNNNNNNNNNNNNNNNNNNNNNNNNNNNNNNNNNNNNNNNNNNNNNNNNNNNNNNNNNNNNNNNNNNNNNNNNNNNNNNNNNNNNNNNNNNNNNNNNNNNNNNNNNNNNNNNN 1560

----:----|----:----|----:----|----:----|----:----|----:----|----:----|----:----|----:----|----:----|----:----|----:----|

X X X X X X X X X X X X X X X X X X X X X X X X X X X X X X X X X X X X X X X X F1

X X X X X X X X X X X X X X X X X X X X X X X X X X X X X X X X X X X X X X X X F2

X X X X X X X X X X X X X X X X X X X X X X X X X X X X X X X X X X X X X X X X F3

1561 NNNNNNNNNNNNNNNNNNNNNNNNNNNNNNNNNNNNNNNNNNNNNNNNNNNNNNNNNNNNNNNNNNNNNNNNNNNNNNNNNNNNNNNNNNNNNNNNNNNNNNNNNNNNNNNNNNNNNNNN 1680

----:----|----:----|----:----|----:----|----:----|----:----|----:----|----:----|----:----|----:----|----:----|----:----|

X X X X X X X X X X X X X X X X X X X X X X X X X X X X X X X X X X X X X X X X F1

X X X X X X X X X X X X X X X X X X X X X X X X X X X X X X X X X X X X X X X X F2

X X X X X X X X X X X X X X X X X X X X X X X X X X X X X X X X X X X X X X X X F3

1681 NNNNNNNNNNNNNNNNNNNNNNNNNNNNNNNNNNNNNNNNNNNNNNNNNNNNNNNNNNNNNNNNNNNNNNNNNNNNNNNNNNNNNNNNNNNNNNNNNNNNNNNNNNNNNNNNNNNNNNNN 1800

----:----|----:----|----:----|----:----|----:----|----:----|----:----|----:----|----:----|----:----|----:----|----:----|

X X X X X X X X X X X X X X X X X X X X X X X X X X X X X X X X X X X X X X X X F1

X X X X X X X X X X X X X X X X X X X X X X X X X X X X X X X X X X X X X X X X F2

X X X X X X X X X X X X X X X X X X X X X X X X X X X X X X X X X X X X X X X X F3

1801 NNNNNNNNNNNNNNNNNNNNNNNNNNNNNNNNNNNNNNNNNNNNNNNNNNNNNNNNNNNNNNNNNNNNNNNNNNNNNNNNNNNNNNNNNNNNNNNNNNNNNNNNNNNNNNNNNNNNNNNN 1920

----:----|----:----|----:----|----:----|----:----|----:----|----:----|----:----|----:----|----:----|----:----|----:----|

X X X X X X X X X X X X X X X X X X X X X X X X X X X X X X X X X X X X X X X X F1

X X X X X X X X X X X X X X X X X X X X X X X X X X X X X X X X X X X X X X X X F2

X X X X X X X X X X X X X X X X X X X X X X X X X X X X X X X X X X X X X X X X F3

1921 NNNNNNNNNNNNNNNNNNNNNNNNNNNNNNNNNNNNNNNNNNNNNNNNNNNNNNNNNNNNNNNNNNNNNNNNNNNNNNNNNNNNNNNNNNNNNNNNNNNNNNNNNNNNNNNNNNNNNNNN 2040

----:----|----:----|----:----|----:----|----:----|----:----|----:----|----:----|----:----|----:----|----:----|----:----|

X X X X X X X X X X X X X X X X X X X X X X X X X X X X X X X X X X X X X X X X F1

X X X X X X X X X X X X X X X X X X X X X X X X X X X X X X X X X X X X X X X X F2

X X X X X X X X X X X X X X X X X X X X X X X X X X X X X X X X X X X X X X X X F3

2041 NNNNNNNNNNNNNNNNNNNNNNNNNNNNNNNNNNNNNNNNNNNNNNNNNNNNNNNNNNNNNNNNNNNNNNNNNNNNNNNNNNNNNNNNNNNNNNNNNNNNNNNNNNNNNNNNNNNNNNNN 2160

----:----|----:----|----:----|----:----|----:----|----:----|----:----|----:----|----:----|----:----|----:----|----:----|

X X X X X X X X X X X X X X X X X X X X X X X X X X X X X X X X X X X X X X X X F1

X X X X X X X X X X X X X X X X X X X X X X X X X X X X X X X X X X X X X X X X F2

X X X X X X X X X X X X X X X X X X X X X X X X X X X X X X X X X X X X X X X X F3

2161 NNNNNNNNNNNNNNNNNNNNNNNNNNNNNNNNNNNNNNNNNNNNNNNNNNNNNNNNNNNNNNNNNNNNNNNNNNNNNNNNNNNNNNNNNNNNNNNNNNNNNNNNNNNNNNNNNNNNNNNN 2280

----:----|----:----|----:----|----:----|----:----|----:----|----:----|----:----|----:----|----:----|----:----|----:----|

X X X X X X X X X X X X X X X X X X X X X X X X X X X X X X X X X X X X X X X X F1

X X X X X X X X X X X X X X X X X X X X X X X X X X X X X X X X X X X X X X X X F2

X X X X X X X X X X X X X X X X X X X X X X X X X X X X X X X X X X X X X X X X F3

2281 NNNNNNNNNNNNNNNNNNNNNNNNNNNNNNNNNNNNNNNNNNNNNNNNNNNNNNNNNNNNNNNNNNNNNNNNNNNNNNNNNNNNNNNNNNNNNNNNNNNNNNNNNNNNNNNNNNNNNNNN 2400

----:----|----:----|----:----|----:----|----:----|----:----|----:----|----:----|----:----|----:----|----:----|----:----|

X X X X X X X X X X X X X X X X X X X X X X X X X X X X X X X X X X X X X X X X F1

X X X X X X X X X X X X X X X X X X X X X X X X X X X X X X X X X X X X X X X X F2

X X X X X X X X X X X X X X X X X X X X X X X X X X X X X X X X X X X X X X X X F3

2401 NNNNNNNNNNNNNNNNNNNNNNNNNNNNNNNNNNNNNNNNNNNNNNNNNNNNNNNNNNNNNNNNNNNNNNNNNNNNNNNNNNNNNNNNNNNNNNNNNNNNNNNNNNNNNNNNNNNNNNNN 2520

----:----|----:----|----:----|----:----|----:----|----:----|----:----|----:----|----:----|----:----|----:----|----:----|

X X X X X X X X X X X X X X X X X X X X X X X X X X X X X X X X X X X X X X X X F1

X X X X X X X X X X X X X X X X X X X X X X X X X X X X X X X X X X X X X X X X F2

X X X X X X X X X X X X X X X X X X X X X X X X X X X X X X X X X X X X X X X X F3

2521 NNNNNNNNNNNNNNNNNNNNNNNNNNNNNNNNNNNNNNNNNNNNNNNNNNNNNNNNNNNNNNNNNNNNNNNNNNNNNNNNNNNNNNNNNNNNNNNNNNNNNNNNNNNNNNNNNNNNNNNN 2640

----:----|----:----|----:----|----:----|----:----|----:----|----:----|----:----|----:----|----:----|----:----|----:----|

X X X X X X X X X X X X X X X X X X X X X X X X X X X X X X X X X X X X X X X X F1

X X X X X X X X X X X X X X X X X X X X X X X X X X X X X X X X X X X X X X X X F2

X X X X X X X X X X X X X X X X X X X X X X X X X X X X X X X X X X X X X X X X F3

2641 NNNNNNNNNNNNNNNNNNNNNNNNNNNNNNNNNNNNNNNNNNNNNNNNNNNNNNNNNNNNNNNNNNNNNNNNNNNNNNNNNNNNNNNNNNNNNNNNNNNNNNNNNNNNNNNNNNNNNNNN 2760

----:----|----:----|----:----|----:----|----:----|----:----|----:----|----:----|----:----|----:----|----:----|----:----|

X X X X X X X X X X X X X X X X X X X X X X X X X X X X X X X X X X X X X X X X F1

X X X X X X X X X X X X X X X X X X X X X X X X X X X X X X X X X X X X X X X X F2

X X X X X X X X X X X X X X X X X X X X X X X X X X X X X X X X X X X X X X X X F3

2761 NNNNNNNNNNNNNNNNNNNNNNNNNNNNNNNNNNNNNNNNNNNNNNNNNNNNNNNNNNNNNNNNNNNNNNNNNNNNNNNNNNNNNNNNNNNNNNNNNNNNNNNNNNNNNNNNNNNNNNNN 2880

----:----|----:----|----:----|----:----|----:----|----:----|----:----|----:----|----:----|----:----|----:----|----:----|

X X X X X X X X X X X X X X X X X X X X X X X X X X X X X X X X X X X X X X X X F1

X X X X X X X X X X X X X X X X X X X X X X X X X X X X X X X X X X X X X X X X F2

X X X X X X X X X X X X X X X X X X X X X X X X X X X X X X X X X X X X X X X X F3

2881 NNNNNNNNNNNNNNNNNNNNNNNNNNNNNNNNNNNNNNNNNNNNNNNNNNNNNNNNNNNNNNNNNNNNNNNNNNNNNNNNNNNNNNNNNNNNNNNNNNNNNNNNNNNNNNNNNNNNNNNN 3000

----:----|----:----|----:----|----:----|----:----|----:----|----:----|----:----|----:----|----:----|----:----|----:----|

X X X X X X X X X X X X X X X X X X X X X X X X X X X X X X X X X X X X X X X X F1

X X X X X X X X X X X X X X X X X X X X X X X X X X X X X X X X X X X X X X X X F2

X X X X X X X X X X X X X X X X X X X X X X X X X X X X X X X X X X X X X X X X F3

3001 NNNNNNNNNNNNNNNNNNNNNNNNNNNNNNNNNNNNNNNNNNNNNNNNNNNNNNNNNNNNNNNNNNNNNNNNNNNNNNNNNNNNNNNNNNNNNNNNNNNNNNNNNNNNNNNNNNNNNNNN 3120

----:----|----:----|----:----|----:----|----:----|----:----|----:----|----:----|----:----|----:----|----:----|----:----|

X X X X X X X X X X X X X X X X X X X X X X X X X X X X X X X X X X X X X X X X F1

X X X X X X X X X X X X X X X X X X X X X X X X X X X X X X X X X X X X X X X X F2

X X X X X X X X X X X X X X X X X X X X X X X X X X X X X X X X X X X X X X X X F3

3121 NNNNNNNNNNNNNNNNNNNNNNNNNNNNNNNNNNNNNNNNNNNNNNNNNNNNNNNNNNNNNNNNNNNNNNNNNNNNNNNNNNNNNNNNNNNNNNNNNNNNNNNNNNNNNNNNNNNNNNNN 3240

----:----|----:----|----:----|----:----|----:----|----:----|----:----|----:----|----:----|----:----|----:----|----:----|

X X X X X X X X X X X X X X X X X X X X X X X X X X X X X X X X X X X X X X X X F1

X X X X X X X X X X X X X X X X X X X X X X X X X X X X X X X X X X X X X X X X F2

X X X X X X X X X X X X X X X X X X X X X X X X X X X X X X X X X X X X X X X X F3

3241 NNNNNNNNNNNNNNNNNNNNNNNNNNNNNNNNNNNNNNNNNNNNNNNNNNNNNNNNNNNNNNNNNNNNNNNNNNNNNNNNNNNNNNNNNNNNNNNNNNNNNNNNNNNNNNNNNNNNNNNN 3360

----:----|----:----|----:----|----:----|----:----|----:----|----:----|----:----|----:----|----:----|----:----|----:----|

X X X X X X X X X X X X X X X X X X X X X X X X X X X X X X X X X X X X X X X X F1

X X X X X X X X X X X X X X X X X X X X X X X X X X X X X X X X X X X X X X X X F2

X X X X X X X X X X X X X X X X X X X X X X X X X X X X X X X X X X X X X X X X F3

3361 NNNNNNNNNNNNNNNNNNNNNNNNNNNNNNNNNNNNNNNNNNNNNNNNNNNNNNNNNNNNNNNNNNNNNNNNNNNNNNNNNNNNNNNNNNNNNNNNNNNNNNNNNNNNNNNNNNNNNNNN 3480

----:----|----:----|----:----|----:----|----:----|----:----|----:----|----:----|----:----|----:----|----:----|----:----|

X X X X X X X X X X X X X X X X X X X X X X X X X X X X X X X X X X X X X X X X F1

X X X X X X X X X X X X X X X X X X X X X X X X X X X X X X X X X X X X X X X X F2

X X X X X X X X X X X X X X X X X X X X X X X X X X X X X X X X X X X X X X X X F3

3481 NNNNNNNNNNNNNNNNNNNNNNNNNNNNNNNNNNNNNNNNNNNNNNNNNNNNNNNNNNNNNNNNNNNNNNNNNNNNNNNNNNNNNNNNNNNNNNNNNNNNNNNNNNNNNNNNNNNNNNNN 3600

----:----|----:----|----:----|----:----|----:----|----:----|----:----|----:----|----:----|----:----|----:----|----:----|

X G P G L R R N G A G T L Q P A R A V G D A T A T S P Q H C I G G G F G V Q R V F1

X G R G C G A T V P A P C S R R E L W V T P L P R P P S T A S G A V L G C N G C F2

X A G A A A Q R C R H P A A G A S C G * R H C H V P P A L H R G R F W G A T G A F3

3601 NNNGGGCCGGGGCTGCGGCGCAACGGTGCCGGCACCCTGCAGCCGGCGCGAGCTGTGGGTGACGCCACTGCCACGTCCCCCCAGCACTGCATCGGGGGCGGTTTTGGGGTGCAACGGGTG 3720

----:----|----:----|----:----|----:----|----:----|----:----|----:----|----:----|----:----|----:----|----:----|----:----|

P A S C S W R E P Q V M P P P R P P T V H R G Q F W G A T G A S T L Q P A R A M F1

Q H P A A G A S R R * C H R P V P Q L C I G G S F G V Q Q V P A P C S R H E P W F2

S I L Q L A R A A G D A T A P S P N C A S G A V L G C N R C Q H P A A G T S H G F3

3721 CCAGCATCCTGCAGCTGGCGCGAGCCGCAGGTGATGCCACCGCCCCGTCCCCCAACTGTGCATCGGGGGCAGTTTTGGGGTGCAACAGGTGCCAGCACCCTGCAGCCGGCACGAGCCATG 3840

----:----|----:----|----:----|----:----|----:----|----:----|----:----|----:----|----:----|----:----|----:----|----:----|

G D A A A A S P Q H C I G G G S G V Q W V P A P C R Q H K L W V M V P C F P P T F1

V M P L P R P P S T A S G V V L G C N G C R H P A G S T S C G * W C R V S L P P F2

* C R C R V P P A L H R G W F W G A M G A G T L Q A A Q A V G D G A V F P S H P F3

3841 GGTGATGCCGCTGCCGCGTCCCCCCAGCACTGCATCGGGGGTGGTTCTGGGGTGCAATGGGTGCCGGCACCCTGCAGGCAGCACAAGCTGTGGGTGATGGTGCCGTGTTTCCCTCCCACC 3960

----:----|----:----|----:----|----:----|----:----|----:----|----:----|----:----|----:----|----:----|----:----|----:----|

Q H C I G G G G F F P E A S P Q Q C G D F P A Y A W N G Y G T H T G W S T S R E F1

S T A S G A A G S S P R R A R S S A E T S P P T P G T A T G P T R A G A R R G R F2

A L H R G R R V L P R G E P A A V R R L P R L R L E R L R D P H G L E H V A G D F3

3961 CAGCACTGCATCGGGGGCGGCGGGTTCTTCCCCGAGGCGAGCCCGCAGCAGTGCGGAGACTTCCCCGCCTACGCCTGGAACGGCTACGGGACCCACACGGGCTGGAGCACGTCGCGGGAG 4080

----:----|----:----|----:----|----:----|----:----|----:----|----:----|----:----|----:----|----:----|----:----|----:----|

I L E A A V L L F Y R * G R G R R A P P R A L P I N P S A K S R C A W I I P L G F1

S W R P P C C S S T A E A A G A E L R R G R C Q * I L L Q S L G V P G S S R W A F2

P G G R R A A L L P L R P R A P S S A A G A A N K S F C K V S V C L D H P V G L F3

4081 ATCCTGGAGGCCGCCGTGCTGCTCTTCTACCGC**TGA**GGCCGCGGGCGCCGAGCTCCGCCGCGGGCGCTGCCAATAAATCCTTCTGCAAAGTCTCGGTGTGCCTGGATCATCCCGTTGGGC 4200

----:----|----:----|----:----|----:----|----:----|----:----|----:----|----:----|----:----|----:----|----:----|----:----|

C A G A R D G L A G F1

A R G L G T G W R G F2

R G G S G R A G G X F3

4201 TGCGCGGGGGCTCGGGACGGGCTGGCGGGG 4230

----:----|----:----|----:----|

**c. Amino acid sequence comparison between ITLN1 proteins from *T. guttatus* (upper sequence) and *A. australis mantelli* (lower sequence) using BLASTP at NCBI.**


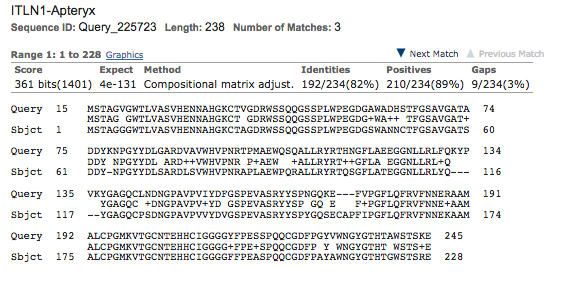

Supplement: Supplementary file 1 — Additional file 1. Sequences of one avian mRNA (a) and exons of one avian gene (b) coding for an intellectin-like protein (ITLN1), and their amino acid sequence comparison (c). [file 12864_2019_6131_MOESM1_ESM.docx]
