# Supplementary material for: Sequence properties of certain GC rich avian genes, their origins and absence from genome assemblies: case studies
Source: BMC Genomics. 2019 Oct 14;20:734. doi: 10.1186/s12864-019-6131-1 (PMC6792250; doi:10.1186/s12864-019-6131-1)
Supplement: Supplementary file 2 — Additional file 2. The sequence of RJF genes coding for the carnitine O-palmitoyltransferase 1 (CPT1C) and the insulin-like peptide 5 (ISNL5). [file 12864_2019_6131_MOESM2_ESM.docx]

**Additional data 2**: The sequence of red jungle fowl (RJF) genes coding for the carnitine O-palmitoyltransferase 1 (CPT1C) and the insulin-like peptide 5 (ISNL5). Sequence translation all in three frames are shown. Coding frames overlapping exons are highlighted in yellow. Splice sites at both intron ends are highlighted in grey and coloured in red. Genes presents in the 5' and 3' regions were investigated to verify whether there was syntenic gene conservation around each of them as described for other non-avian vertebrate lineages (data available at http://www.genomicus.biologie.ens.fr/genomicus-92.01/cgi-bin/search.pl). Their names are shown in front of each sequence in its legend. For both genes, we observed that their genic environment was different compared to other vertebrate lineages. This suggests that both genes were independently translocated into another chromosomal locus by recombination, very likely early during the evolution of the avian lineage. In galGal6 CPT1C and ISNL5 were located and annotated on the minus strand of chromosome 5 between positions 16814219 and 16837319 and on the minus strand of chromosome 8 between positions 28916675 and 28920087 respectively.

Several avian sequences of the CPT1C protein are available in public databases, including that of the RJF (sequence ID: NP_001012916.1). The 18 exons of the gene encoding this protein are available in two RJF models, within the scaffold AADN05000817.1 and the Gallus gallus breed Yeonsan Ogye chromosome 1 (positions 75,340,216-75,365,132). Below is the sequence of scaffold AADN05000817.1 between positions 191400-214500 (Gallus gallus breed chicken, inbred line UCD001 isolate RJF #256 Contig65.5, whole genome shotgun sequence). Coding frame in exons are highlighted in yellow. In non-avian vertebrates, the CPT1C gene is surrounded upstream by a gene encoding the protein arginine methyltransferase 1 (PMRT1) and downstream by a gene encoding the testis specific serine kinase substrate (TSKS). These two genes were not found in the AADN05000817.1 scaffold. The PMRT1 gene was located in another region, in the scaffold AADN05000817.1 (Contig81.2) for the chicken and in chromosome 1 for *Gallus gallus* breed Yeonsan Ogye (positions 75,340,216-75,365,132). In the 50,000 nucleotides located upstream and downstream of the CPT1C gene we found a gene encoding a reverse transcriptase (Sequence ID: AAC60281.1) between positions 159153 to 189867 and within those downstream a RJF pol-like protein ENS-3 (Sequence ID: NP_989963.1) between positions 231030 to 233210 of the scaffold AADN05000817.1.

L L C Y A L Y Q T R F * Y L C T E S N L F F C V F F L T E L S P K M A E A H Q A V A F Q F T V T P D F1

Y Y V M L Y T R L D S S T F V Q S L I Y F F V S F F * Q N S L Q K W Q K L I K Q * H F N L Q * L Q M F2

I M L C F I P D * I L V P L Y R V * F I F L C L F F N R T L S K N G R S S S S S S I S I Y S N S R W F3

1 TTATTATGTTATGCTTTATACCAGACTAGATTCTAGTACCTTTGTACAGAGTCTAATTTATTTTTTTGTGTCTTTTTTTTAACAGAACTCTCTCCAAAAATGGCAGAAGCTCATCAAGCAGTAGCATTTCAATTTACAGTAACTCCAGAT 150

----:----|----:----|----:----|----:----|----:----|----:----|----:----|----:----|----:----|----:----|----:----|----:----|----:----|----:----|----:----|

G I D L R M S H E A L K Q I Y L S G V H S W K K K F I R F K V L K T S Q A Y Q T * G * F R F S * I H F1

G L T C E * V M R L S N K F T Y L V S I H G R K S S S D S R Y L K P V K P T K L E G N L G S H R Y I F2

D * L A N E S * G S Q T N L P I W C P F M E E K V H Q I Q G T * N Q S S L P N L R V I * V L I D T * F3

151 GGGATTGACTTGCGAATGAGTCATGAGGCTCTCAAACAAATTTACCTATCTGGTGTCCATTCATGGAAGAAAAAGTTCATCAGATTCAAG**GT**ACTTAAAACCAGTCAAGCCTACCAAACTTGAGGGTAATTTAGGTTCTCATAGATACAT 300

----:----|----:----|----:----|----:----|----:----|----:----|----:----|----:----|----:----|**--**--:----|----:----|----:----|----:----|----:----|----:----|

K S F V * A K L N F * A S L A Y K V P Y S E * L V R I L V N E G * * G S I S T G * C C A K I T M S R F1

K V L F K Q N L I F R P L W H I K F L T A N D L * E F W L T R D S K E A S A L A D A V L K L Q C Q G F2

K F C L S K T * F L G L F G I * S S L Q R M T C E N F G * R G I V R K H Q H W L M L C * N Y N V K A F3

301 AAAAGTTTTGTTTAAGCAAAACTTAATTTTTAGGCCTCTTTGGCATATAAAGTTCCTTACAGCGAATGACTTGTGAGAATTTTGGTTAACGAGGGATAGTAAGGAAGCATCAGCACTGGCTGATGCTGTGCTAAAATTACAATGTCAAGG 450

----:----|----:----|----:----|----:----|----:----|----:----|----:----|----:----|----:----|----:----|----:----|----:----|----:----|----:----|----:----|

L K G Y F S L I C S M E T R Q S R Y * K L I I P L F Y S I L N S S Y N Y I I S I C I D Q K Y N R G F F1

* K D I S V * S V L W K Q D R A G I R N * * Y L Y F I L F S I A R I I I * Y L F A * I K N I I V A F F2

E R I F Q F D L F Y G N K T E Q V L E I N N T F I L F Y S Q * L V * L Y N I Y L H R S K I * S W L L F3

451 CTGAAAGGATATTTCAGTTTGATCTGTTCTATGGAAACAAGACAGAGCAGGTATTAGAAATTAATAATACCTTTATTTTATTCTATTCTCAATAGCTCGTATAATTATATAATATCTATTTGCATAGATCAAAAATATAATCGTGGCTTT 600

----:----|----:----|----:----|----:----|----:----|----:----|----:----|----:----|----:----|----:----|----:----|----:----|----:----|----:----|----:----|

* K S C Y L Q R Y K T T F L I Y V L Y H K * T F K L G K N V C C F * K L P E H Q S W I N Y A S S N V F1

K N H V T C R G T K L R S * F M F F T T N K L S N * E K M F V V F K S Y L N I N L G * I M P A V M F F2

K I M L L A E V Q N Y V L D L C S L P Q I N F Q I R K K C L L F L K V T * T S I L D K L C Q Q * C F F3

601 TAAAAATCATGTTACTTGCAGAGGTACAAAACTACGTTCTTGATTTATGTTCTTTACCACAAATAAACTTTCAAATTAGGAAAAAATGTTTGTTGTTTTTAAAAGTTACCTGAACATCAATCTTGGATAAATTATGCCAGCAGTAATGTT 750

----:----|----:----|----:----|----:----|----:----|----:----|----:----|----:----|----:----|----:----|----:----|----:----|----:----|----:----|----:----|

S D V C Y G * K * N R Y T I Q I F V N F L F C S * N H K W Y L F Y * * Y M R * A T S * F Q Y L F Q K F1

L M C V M D R S E T G I R Y R F L * T S Y F A H R I I N G I C S I S S T * G K L Q V D F S I S F K N F2

* C V L W I E V K Q V Y D T D F C E L L I L L I E S * M V S V L L V V H E V S Y K L I S V S L S K T F3

751 TCTGATGTGTGTTATGGATAGAAGTGAAACAGGTATACGATACAGATTTTTGTGAACTTCTTATTTTGCTCATAGAATCATAAATGGTATCTGTTCTATTAGTAGTACATGAGGTAAGCTACAAGTTGATTTCAGTATCTCTTTCAAAAA 900

----:----|----:----|----:----|----:----|----:----|----:----|----:----|----:----|----:----|----:----|----:----|----:----|----:----|----:----|----:----|

H T F K K * S * * K H L K T L R R A V * V T V F L C L V Q * R I F C P V F V * G G L I M G L W N H N F1

I L L K S S L N K N I L K L * E G Q F K * Q F F C V L F S R E S F A L Y L F R E A * * W D Y G I I I F2

Y F * K V V L I K T S * N F E K G S L S N S F F V S C S V E N L L P C I C L G R P N N G I M E S * L F3

901 CATACTTTTAAAAAGTAGTCTTAATAAAAACATCTTAAAACTTTGAGAAGGGCAGTTTAAGTAACAGTTTTTTTGTGTCTTGTTCAGTAGAGAATCTTTTGCCCTGTATTTGTTTAGGGAGGCCTAATAATGGGATTATGGAATCATAAT 1050

----:----|----:----|----:----|----:----|----:----|----:----|----:----|----:----|----:----|----:----|----:----|----:----|----:----|----:----|----:----|

W K C H L F Q R T L L I S V L L L E L V A Q T V * S K K * L L V L * E F P V A I K L S * N P * E K M F1

G N V I Y S K E L C * F Q C F Y * N W W H K L Y S L R N N Y W Y C E N F R * R S N L A K I H E R K C F2

E M S F I P K N F A D F S A F T R T G G T N C I V * E I I I G I V R I S G S D Q T * L K S M R E N A F3

1051 TGGAAATGTCATTTATTCCAAAGAACTTTGCTGATTTCAGTGCTTTTACTAGAACTGGTGGCACAAACTGTATAGTCTAAGAAATAATTATTGGTATTGTGAGAATTTCCGGTAGCGATCAAACTTAGCTAAAATCCATGAGAGAAAATG 1200

----:----|----:----|----:----|----:----|----:----|----:----|----:----|----:----|----:----|----:----|----:----|----:----|----:----|----:----|----:----|

L D N L V * N * M T * M Y * F L F L S M V I * S Q P L W Y E K I M * R L F F P E W N Y H W C L S C * F1

W I I L F K I K * L K C I D F C F F P W * Y E V N R F G M K K L C E G F S F Q N G I I T G V Y P A S F2

G * S C L K L N D L N V L I F V S F H G N M K S T A L V * K N Y V K A F L S R M E L S L V F I L L A F3

1201 CTGGATAATCTTGTTTAAAATTAAATGACTTAAATGTATTGATTTTTGTTTCTTTCCATGGTAATATGAAGTCAACCGCTTTGGTATGAAAAAATTATGTGAAGGCTTTTCTTTCC**AG**AATGGAATTATCACTGGTGTTTATCCTGCTAG 1350

----:----|----:----|----:----|----:----|----:----|----:----|----:----|----:----|----:----|----:----|----:----|----:-**--**-|----:----|----:----|----:----|

P L * L A Y C S C G C D V N N V C Q N * S F F R N N S * N Q P N P * H N V S S L K T Y C S L N Q I S F1

P S S W L I V V V G V M S T M Y A K I D P S L G I I A K I N R T L D T T * V V * K L T V L * T K S V F2

P L A G L L * L W V * C Q Q C M P K L I L L * E * * L K S T E P L T Q R K * S E N L L F S K P N Q F F3

1351 CCCCTCTAGCTGGCTTATTGTAGTTGTGGGTGTGATGTCAACAATGTATGCCAAAATTGATCCTTCTTTAGGAATAATAGCTAAAATCAACCGAACCCTTGACACAAC**GT**AAGTAGTCTGAAAACTTACTGTTCTCTAAACCAAATCAGT 1500

----:----|----:----|----:----|----:----|----:----|----:----|----:----|----:----|----:----|----:----|----:---**-|**----:----|----:----|----:----|----:----|

F M C N C F S S K S Y * S V P V F E Q Y V C V R V S V S L N A * S Y * T C R L F Y Q Y C C I A V I R F1

L C V T A F H L N P I K A Y Q S L N S M F V L E * A * V L M H K A I K R V G C F T S I A A L L L Y V F2

Y V * L L F I * I L L K R T S L * T V C L C * S K R K S * C I K L L N V * V V L P V L L H C C Y T C F3

1501 TTTATGTGTAACTGCTTTTCATCTAAATCCTATTAAAGCGTACCAGTCTTTGAACAGTATGTTTGTGTTAGAGTAAGCGTAAGTCTTAATGCATAAAGCTATTAAACGTGTAGGTTGTTTTACCAGTATTGCTGCATTGCTGTTATACGT 1650

----:----|----:----|----:----|----:----|----:----|----:----|----:----|----:----|----:----|----:----|----:----|----:----|----:----|----:----|----:----|

V W E V H L A T S V A F V L A I G E D V W K G V Q * E G A E R H K A C V M P A F G T T E C R F A I L F1

F G K C T W Q L L L P L F W P * E R M F G K E F N K K G Q R G I K L A * C L P L V P Q N V G L Q Y * F2

L G S A L G N F C C L C S G H R R G C L E R S S I R R G R E A * S L R N A C L W Y H R M * V C N I K F3

1651 GTTTGGGAAGTGCACTTGGCAACTTCTGTTGCCTTTGTTCTGGCCATAGGAGAGGATGTTTGGAAAGGAGTTCAATAAGAAGGGGCAGAGAGGCATAAAGCTTGCGTAATGCCTGCCTTTGGTACCACAGAATGTAGGTTTGCAATATTA 1800

----:----|----:----|----:----|----:----|----:----|----:----|----:----|----:----|----:----|----:----|----:----|----:----|----:----|----:----|----:----|

S S P * S L N * C * L T N A A F C F K * W L Y V K P N T E H C E W N T F W H R A L G C P Y R H N A V F1

V L L K A * T D V N * L M L P F V L N S G Y M S N Q T Q N I V S G I L F G T G L W V A L I V T M R Y F2

F S L K L K L M L T D * C C L L F * I V A I C Q T K H R T L * V E Y F L A Q G F G L P L S S Q C G T F3

1801 AGTTCTCCTTAAAGCTTAAACTGATGTTAACTGACTAATGCTGCCTTTTGTTTTAAAT**AG**TGGCTATATGTCAAACCAAACACAGAACATTGTGAGTGGAATACTTTTTGGCACAGGGCTTTGGGTTGCCCTTATCGTCACAATGCGGTA 1950

----:----|----:----|----:----|----:----|----:----|----:---**-|**----:----|----:----|----:----|----:----|----:----|----:----|----:----|----:----|----:----|

L P E N A A F L S W L D V C * T R Q T F C R H Q A V D G K D I L S F C S V S V C M W S V F K A L K * F1

S L K M L L S Y H G W M F A E H G K L S A G T K L W M V R I Y C L F V L S L C V C G L F S K P * S R F2

P * K C C F P I M A G C L L N T A N F L Q A P S C G W * G Y T V F L F C L C V Y V V C F Q S P K V D F3

1951 CTCCCTGAAAATGCTGCTTTCCTATCATGGCTGGATGTTTGCTGAACACGGCAAACTTTCTGCAGGCACCAAGCTGTGGATGGTAAGGATATACTGTCTTTTTGTTCTGTCTCTGTGTGTATGTGGTCTGTTTTCAAAGCCCTAAAGTAG 2100

----:----|----:----|----:----|----:----|----:----|----:----|----:----|----:----|----:----|----:----|----:----|----:----|----:----|----:----|----:----|

I V L * V F G F F F L S C L D F L C Q G T R P V G Y F Y * L L S S F R C C N * A I Q Y G L V E G K A F1

* F C R F L V F F F C L A W I F C V R G Q G L L D I F T N Y C H P L D A V I E L F N M A L W K E K Q F2

S F V G F W F F F S V L L G F S V S G D K A C W I F L L I T V I L * M L * L S Y S I W P C G R K S K F3

2101 ATAGTTTTGTAGGTTTTTGGTTTTTTTTTTCTGTCTTGCTTGGATTTTCTGTGTCAGGGGACAAGGCCTGTTGGATATTTTTACTAATTACTGTCATCCTTTAGATGCTGTAATTGAGCTATTCAATATGGCCTTGTGGAAGGAAAAGCA 2250

----:----|----:----|----:----|----:----|----:----|----:----|----:----|----:----|----:----|----:----|----:----|----:----|----:----|----:----|----:----|

N T L R R T S K F P S A L G L * L K C L L L Y V Q P Q L K L R L * N K K T F L F S P E S T T L I L S F1

T L * E E R L N F P A P W D C D S N A Y F S M C N H S L S S G Y E T K K P S C S L P K A L L * F F L F2

H F K K N V * I S Q R L G T V T Q M P T S L C A T T A * A Q A M K Q K N L L V L S R K H Y S N S F L F3

2251 AACACTTTAAGAAGAACGTCTAAATTTCCCAGCGCCTTGGGACTGTGACTCAAATGCCTACTTCTCTATGTGCAACCACAGCTTAAGCTCAGGCTATGAAACAAAAAAACCTTCTTGTTCTCTCCCGAAAGCACTACTCTAATTCTTTCT 2400

----:----|----:----|----:----|----:----|----:----|----:----|----:----|----:----|----:----|----:----|----:----|----:----|----:----|----:----|----:----|

* S R N V G I F G H P F * T L P L K K T T V C P F K * S * R * D S K Q L E K T H E W V A C Y T I Y T F1

K V E M L V F L V I H S K P Y L * R K Q Q Y V P S N D L S G R T P S S W R K H M S G L L V T Q F T L F2

K * K C W Y F W S S I L N L T F K E N N S M S L Q M I L A V G L Q A V G E N T * V G C L L H N L H L F3

2401 TAAAGTAGAAATGTTGGTATTTTTGGTCATCCATTCTAAACCTTACCTTTAAAGAAAACAACAGTATGTCCCTTCAAATGATCTTAGCGGTAGGACTCCAAGCAGTTGGAGAAAACACATGAGTGGGTTGCTTGTTACACAATTTACACT 2550

----:----|----:----|----:----|----:----|----:----|----:----|----:----|----:----|----:----|----:----|----:----|----:----|----:----|----:----|----:----|

* R * D W N D V A S P D N * V I * K G F K R L P S P L E A V V G V G K V C I G A L K G G V D F Y Y S F1

K D R T G M M W L L L T I K L Y E K G L R G C H L P * R L * * V S G R Y V * V L * K V E W I F I I Q F2

K I G L E * C G F S * Q L S Y M K R V * E A A I S L R G C S R C R E G M Y R C S E R W S G F L L F R F3

2551 TAAAGATAGGACTGGAATGATGTGGCTTCTCCTGACAATTAAGTTATATGAAAAGGGTTTAAGAGGCTGCCATCTCCCTTAGAGGCTGTAGTAGGTGTCGGGAAGGTATGTATAGGTGCTCTGAAAGGTGGAGTGGATTTTTATTATTCA 2700

----:----|----:----|----:----|----:----|----:----|----:----|----:----|----:----|----:----|----:----|----:----|----:----|----:----|----:----|----:----|

G S V V * T L S I V D V E F M C P P K I K I S F H S L K E I K R C V S T * * Y I M E V V Q Q Q I S R F1

A Q L F K L F P L L M * N L C A L L R * K L V S T A L K K S R D V F P R N D I L W K L F N N R Y P E F2

L S C L N S F H C * C R I Y V P S * D K N * F P Q P * R N Q E M C F H V M I Y Y G S C S T T D I Q N F3

2701 GGCTCAGTTGTTTAAACTCTTTCCATTGTTGATGTAGAATTTATGTGCCCTCCTAAGATAAAAATTAGTTTCCACAGCCTTAAAGAAATCAAGAGATGTGTTTCCACGTAATGATATATTATGGAAGTTGTTCAACAACAGATATCCAGA 2850

----:----|----:----|----:----|----:----|----:----|----:----|----:----|----:----|----:----|----:----|----:----|----:----|----:----|----:----|----:----|

I Y I T V * Y * F V T N E I S N Q * F L L E A V S R A S * L E F V Q Y R L * M V R L S V T C L * L S F1

Y T L Q F N I S L L Q M K F L I N S F S W K Q C Q E P R D L N L Y S T G S E W * G F L S L A C S S L F2

I H Y S L I L V C Y K * N F * S I V S P G S S V K S L V T * I C T V Q A L N G K A F C H L L V A L S F3

2851 ATATACATTACAGTTTAATATTAGTTTGTTACAAATGAAATTTCTAATCAATAGTTTCTCCTGGAAGCAGTGTCAAGAGCCTCGTGACTTGAATTTGTACAGTACAGGCTCTGAATGGTAAGGCTTTCTGTCACTTGCTTGTAGCTCTCT 3000

----:----|----:----|----:----|----:----|----:----|----:----|----:----|----:----|----:----|----:----|----:----|----:----|----:----|----:----|----:----|

L F G F S A C P T K Q I C * G C * S Y L I T L A I N S M Y C S A T Y H A L L H I I F I Y F G I * Y A F1

C L G F Q H V L P N R Y V K A V N L T L * H * P * T V C T V V L L T T L C C I L Y L Y T S V Y D M L F2

V W V F S M S Y Q T D M L R L L I L P Y N T S H K Q Y V L * C Y L P R S V A Y Y I Y I L R Y M I C C F3

3001 CTGTTTGGGTTTTCAGCATGTCCTACCAAACAGATATGTTAAGGCTGTTAATCTTACCTTATAACACTAGCCATAAACAGTATGTACTGTAGTGCTACTTACCACGCTCTGTTGCATATTATATTTATATACTTCGGTATATGATATGCT 3150

----:----|----:----|----:----|----:----|----:----|----:----|----:----|----:----|----:----|----:----|----:----|----:----|----:----|----:----|----:----|

A Y I R S I L L C I T C G L K T G H Y T G M P N S I S S L L N M K L Y F I P C F F * V V P T K E K T F1

R I F V L F C C V L H V V * K Q A T I Q V C P T V S H L C L I * S F I S S L A F F R * F Q Q K R R L F2

V Y S F Y F A V Y Y M W F K N R P L Y R Y A Q Q Y L I S A * Y E A L F H P L L F L G S S N K R E D W F3

3151 GCGTATATTCGTTCTATTTTGCTGTGTATTACATGTGGTTTAAAAACAGGCCACTATACAGGTATGCCCAACAGTATCTCATCTCTGCTTAATATGAAGCTTTATTTCATCCCTTGCTTTTTTTAGGTAGTTCCAACAAAAGAGAAGACT 3300

----:----|----:----|----:----|----:----|----:----|----:----|----:----|----:----|----:----|----:----|----:----|----:----|----:----|----:----|----:----|

G F L I K Q H Q L P W L S K P F L S R R D F P V H V L L I F F F F F P I * F F M L I N S I T I Y S Q F1

V S L * N S T N Y L G F L N L S Y P G G I F L C M C C * F F F F F S P S D F S C * L T A * Q F T H N F2

F P Y K T A P T T L A F * T F L I Q E G F S C A C V A D F F F F F P H L I F H A D * Q H N N L L T I F3

3301 GGTTTCCTTATAAAACAGCACCAACTACCTTGGCTTTCTAAACCTTTCTTATCCAGGAGGGATTTTCCTGTGCATGTGTTGCTGATTTTTTTTTTTTTTTTCCCCATCTGATTTTTCATGCTGATTAACAGCATAACAATTTACTCACAA 3450

----:----|----:----|----:----|----:----|----:----|----:----|----:----|----:----|----:----|----:----|----:----|----:----|----:----|----:----|----:----|

Y F S F V Q I V L Q I E N L C C D F S C S I N * L A G * L K V * W P I * W K Y F S F * V I R F F * * F1

I F P L F K * S C R * K I C A A I S A A V L I D W Q A D * K S D G P S N G N I S A S K * L G F S D E F2

F F L C S N S P A D R K S V L R F Q L Q Y * L I G R L I E S L M A H L M E I F Q L L S N * V F L M S F3

3451 TATTTTTCCTTTGTTCAAATAGTCCTGCAGATAGAAAATCTGTGCTGCGATTTCAGCTGCAGTATTAATTGATTGGCAGGCTGATTGAAAGTCTGATGGCCCATCTAATGGAAATATTTCAGCTTCTAAGTAATTAGGTTTTTCTGATGA 3600

----:----|----:----|----:----|----:----|----:----|----:----|----:----|----:----|----:----|----:----|----:----|----:----|----:----|----:----|----:----|

V L G C E I R K A M K D V C P S D H F N G L N L L S K L V V E R V C C I C Q L N G C S L S E E N L S F1

Y * D V K S E K Q * R M C V R Q I T S M V S I S * V N L * L S V F V V F V S * T G A L C L K K I S A F2

I R M * N Q K S N E G C V S V R S L Q W S Q S L K * T C S * A C L L Y L S V E R V L S V * R K S Q P F3

3601 GTATTAGGATGTGAAATCAGAAAAGCAATGAAGGATGTGTGTCCGTCAGATCACTTCAATGGTCTCAATCTCTTAAGTAAACTTGTAGTTGAGCGTGTTTGTTGTATTTGTCAGTTGAACGGGTGCTCTCTGTCTGAAGAAAATCTCAGC 3750

----:----|----:----|----:----|----:----|----:----|----:----|----:----|----:----|----:----|----:----|----:----|----:----|----:----|----:----|----:----|

L A W L K S S G I K * S P S I G S R F S Y Q K L W C F F S P G F K F V L A V K H N Q I S L G * * P W F1

L P G * R V L E * N S L Q V L E V D F L T K S Y G V F S H L A L S L S * P * S T I R F H W A D D P G F2

C L A E E F W N K I V S K Y W K * I F L P K A M V F F L T W L * V C L S R E A Q S D F I G L M T L V F3

3751 CTTGCCTGGCTGAAGAGTTCTGGAATAAAATAGTCTCCAAGTATTGGAAGTAGATTTTCTTACCAAAAGCTATGGTGTTTTTTCTCACCTGGCTTTAAGTTTGTCTTAGCCGTGAAGCACAATCAGATTTCATTGGGCTGATGACCCTGG 3900

----:----|----:----|----:----|----:----|----:----|----:----|----:----|----:----|----:----|----:----|----:----|----:----|----:----|----:----|----:----|

C T * Q I S A V G R S F L L V M L * N C * W L C Y L K Q P P C * Q P P A V L I K E M E R E V A V F I F1

A P S R S L L W G G L F C * * C S E I V N G F A T * N S L L A D N L Q L C * S R K W R G K W L F S * F2

H L A D L C C G E V F F A S N A L K L L M A L L P E T A S L L T T S S C A D Q G N G E G S G C F H K F3

3901 TGCACCTAGCAGATCTCTGCTGTGGGGAGGTCTTTTTTGCTAGTAATGCTCTGAAATTGTTAATGGCTTTGCTACCTGAAACAGCCTCCTTGCTGACAACCTCCAGCTGTGCTGATCAAGGAAATGGAGAGGGAAGTGGCTGTTTTCATA 4050

----:----|----:----|----:----|----:----|----:----|----:----|----:----|----:----|----:----|----:----|----:----|----:----|----:----|----:----|----:----|

S S V F S C S * F G * R G Q V L A I Q * * K D I L S F L L S S A H W N T V Y * A L Y N I L Y R M L S F1

V V F S V V R D L G E E G K Y * Q F S S E R T S S V F F C L Q P I G T Q Y T E L Y T I Y Y I G C * A F2

* C F Q L F V I W V K R A S I S N S V V K G H P Q F S S V F S P L E H S I L S F I Q Y T I * D V E P F3

4051 AGTAGTGTTTTCAGTTGTTCGTGATTTGGGTGAAGAGGGCAAGTATTAGCAATTCAGTAGTGAAAGGACATCCTCAGTTTTCTTCTGTCTTCAGCCCATTGGAACACAGTATACTGAGCTTTATACAATATACTATATAGGATGTTGAGC 4200

----:----|----:----|----:----|----:----|----:----|----:----|----:----|----:----|----:----|----:----|----:----|----:----|----:----|----:----|----:----|

Q D C T S * E V N C * F N T * F Y F H A Q M G * C I S I S F H A * S R I C L A E S F G L V H V T T E F1

K I V L H K K * T V S L I L D F T F M P K W G D V Y Q Y P S M H S L E F A W L R V S V * C M S Q L R F2

R L Y F I R S E L L V * Y L I L L S C P N G V M Y I N I L P C I V * N L L G * E F R F S A C H N * E F3

4201 CAAGATTGTACTTCATAAGAAGTGAACTGTTAGTTTAATACTTGATTTTACTTTCATGCCCAAATGGGGTGATGTATATCAATATCCTTCCATGCATAGTCTAGAATTTGCTTGGCTGAGAGTTTCGGTTTAGTGCATGTCACAACTGAG 4350

----:----|----:----|----:----|----:----|----:----|----:----|----:----|----:----|----:----|----:----|----:----|----:----|----:----|----:----|----:----|

R Y S E T E A A F L S S V S W R M R D A V K F E T E S G I V V F G F F C L F V Y W F L I T P G L Y N F1

G I Q R Q K Q H F Y L Q S R G E C G M L * N L K L S L E L L S L V F F V C L F I G F * * H Q D C I T F2

V F R D R S S I S I F S L V E N A G C C K I * N * V W N C C L W F F L F V C L L V F D N T R T V * H F3

4351 AGGTATTCAGAGACAGAAGCAGCATTTCTATCTTCAGTCTCGTGGAGAATGCGGGATGCTGTAAAATTTGAAACTGAGTCTGGAATTGTTGTCTTTGGTTTTTTTTGTTTGTTTGTTTATTGGTTTTTGATAACACCAGGACTGTATAAC 4500

----:----|----:----|----:----|----:----|----:----|----:----|----:----|----:----|----:----|----:----|----:----|----:----|----:----|----:----|----:----|

I G M L A Q L D V F K S C G * L P G T N L F L K K L P V N L Y C * C * A V T L K N I K T * S Q S I I F1

L A C L H N L M C S N R A V D Y L G Q T C S * R N S L * I C T V D A E L * L * K I S K H D P K A * L F2

W H A C T T * C V Q I V R L I T W D K L V L K E T P C K S V L L M L S C N F E K Y Q N M I P K H N Y F3

4501 ATTGGCATGCTTGCACAACTTGATGTGTTCAAATCGTGCGGTTGATTACCTGGGACAAACTTGTTCTTAAAGAAACTCCCTGTAAATCTGTACTGTTGATGCTGAGCTGTAACTTTGAAAAATATCAAAACATGATCCCAAAGCATAATT 4650

----:----|----:----|----:----|----:----|----:----|----:----|----:----|----:----|----:----|----:----|----:----|----:----|----:----|----:----|----:----|

I N E R Y C Y A G C L T E * F L F W S A I E V G F S I A Y F I S K I S V G T F A F * S L * R K M N E F1

L M N A I V M R D A * L N D S C F G V Q L R W V F L L P I L * V K Y Q W V R L L F N L Y R G K * M R F2

* * T L L L C G M L D * M I P V L E C N * G G F F Y C L F Y K * N I S G Y V C F L I F I E E N E * G F3

4651 ATTAATGAACGCTATTGTTATGCGGGATGCTTGACTGAATGATTCCTGTTTTGGAGTGCAATTGAGGTGGGTTTTTCTATTGCCTATTTTATAAGTAAAATATCAGTGGGTACGTTTGCTTTTTAATCTTTATAGAGGAAAATGAATGAG 4800

----:----|----:----|----:----|----:----|----:----|----:----|----:----|----:----|----:----|----:----|----:----|----:----|----:----|----:----|----:----|

E G H * L T A S I F Y * D R G V S Y V N S I A * * P E D C L G S Q E V S M M L F F F K * C V V Y F R F1

K G T N * L P L F S T E I E V C R M * T V L P N D Q K I V W E V R K S P * C S F S S N D V L F T L E F2

R A L T N C L Y F L L R S R C V V C E Q Y C L M T R R L F G K S G S L H D A L F L Q M M C C L L * K F3

4801 GAAGGGCACTAACTAACTGCCTCTATTTTCTACTGAGATCGAGGTGTGTCGTATGTGAACAGTATTGCCTAATGACCAGAAGATTGTTTGGGAAGTCAGGAAGTCTCCATGATGCTCTTTTTCTTCAAATGATGTGTTGTTTACTTTAGA 4950

----:----|----:----|----:----|----:----|----:----|----:----|----:----|----:----|----:----|----:----|----:----|----:----|----:----|----:----|----:----|

R * F A I A F L P L S N N D K G D S W L N L V * C C L L V K G T P S C V I P Y H S V I F L C G T * * F1

D S L P L P F C L C Q T M I K V I A G * I * S S V A Y W * K E L P A V L F L I T L S F S F V E H N K F2

I V C H C L F A F V K Q * * R * * L A K F S L V L L T G E R N S Q L C Y S L S L C H F P L W N I I N F3

4951 AGATAGTTTGCCATTGCCTTTTTGCCTTTGTCAAACAATGATAAAGGTGATAGCTGGCTAAATTTAGTCTAGTGTTGCTTACTGGTGAAAGGAACTCCCAGCTGTGTTATTCCTTATCACTCTGTCATTTTCCTTTGTGGAACATAATAA 5100

----:----|----:----|----:----|----:----|----:----|----:----|----:----|----:----|----:----|----:----|----:----|----:----|----:----|----:----|----:----|

I E V Y V L I L F F F R L L * N S S R D G S P C C T V S R R L Y H D C Q F R P L K I Q S T G C V E F F1

L K C M F * F C F S S D S C K T L L G T E A H V V Q F P D V F T T I A S S G R * R Y S Q Q G V W N S F2

* S V C F D F V F L Q T L V K L F S G R K P M L Y S F Q T S L P R L P V P A V K D T V N R V C G I Q F3

5101 ATTGAAGTGTATGTTTTGATTTTGTTTTTCTTCAGACTCTTGTAAAACTCTTCTCGGGACGGAAGCCCATGTTGTACAGTTTCCAGACGTCTTTACCACGATTGCCAGTTCCGGCCGTTAAAGATACAGTCAACAGGGTGTGTGGAATTC 5250

----:----|----:----|----:----|----:----|----:----|----:----|----:----|----:----|----:----|----:----|----:----|----:----|----:----|----:----|----:----|

S S L S L V T L N P Y L D A F V F L S E C F V F V M S * N R Y V V V S I L S Y A L N L N T * K L R A F1

V H Y L W * H * I H I * M P L Y F C R S V L F L S C L K I V M W W Y Q Y Y L M H * I L T R R N * G L F2

F I I F G N I E S I F R C L C I F V G V F C F C H V L K S L C G G I N I I L C I K S * H V E I K G * F3

5251 AGTTCATTATCTTTGGTAACATTGAATCCATATTTAGATGCCTTTGTATTTTTGTCGGAGTGTTTTGTTTTTGTCATGTCTTAAAATCGTTATGTGGTGGTATCAATATTATCTTATGCATTAAATCTTAACACGTAGAAATTAAGGGCT 5400

----:----|----:----|----:----|----:----|----:----|----:----|----:----|----:----|----:----|----:----|----:----|----:----|----:----|----:----|----:----|

K T N H * N * * Q L E A D L L H F M L G V T V S D G T L S N E T * Y T G L S V H L V F V I F H S M H F1

R Q T T E T D D S W K L T C F I S C W V L R C L M E H F P M R H D T Q G F Q C I L Y L L Y S I A C I F2

D K P L K L M T V G S * L A S F H V G C Y G V * W N T F Q * D M I H R A F S A S C I C Y I P * H A * F3

5401 AAGACAAACCACTGAAACTGATGACAGTTGGAAGCTGACTTGCTTCATTTCATGTTGGGTGTTACGGTGTCTGATGGAACACTTTCCAATGAGACATGATACACAGGGCTTTCAGTGCATCTTGTATTTGTTATATTCCATAGCATGCAT 5550

----:----|----:----|----:----|----:----|----:----|----:----|----:----|----:----|----:----|----:----|----:----|----:----|----:----|----:----|----:----|

R S M C F M T E K A * C S S S K V Y K * G C V L Q K L K V * R R L K E K R N R L K L T S * V L * * * F1

E V C V L * Q K K H S V V V A K C I S R A A C Y R N * R S E G D * R K R E I A * S * R V E S F D N D F2

K Y V F Y D R K S I V * * * Q S V * V G L R V T E T E G L K E T E G K E K S L K A D E L S P L I M I F3

5551 AGAAGTATGTGTTTTATGACAGAAAAAGCATAGTGTAGTAGTAGCAAAGTGTATAAGTAGGGCTGCGTGTTACAGAAACTGAAGGTCTGAAGGAGACTGAAGGAAAAGAGAAATCGCTTAAAGCTGACGAGTTGAGTCCTTTGATAATGA 5700

----:----|----:----|----:----|----:----|----:----|----:----|----:----|----:----|----:----|----:----|----:----|----:----|----:----|----:----|----:----|

* I C S L K C F L L W R G E C I F R R V T F S T C D L F A N F W K R S H I Y S W Q A N V * K K S K Q F1

K Y V L * N A F F S G E E N V F L E E * P F P R V I C L Q T S G K G L I F I V G R Q M F R R K V N R F2

N M F F K M L S S L E R R M Y F * K S D L F H V * F V C K L L E K V S Y L * L A G K C L E E K * T E F3

5701 TAAATATGTTCTTTAAAATGCTTTCTTCTCTGGAGAGGAGAATGTATTTTTAGAAGAGTGACCTTTTCCACGTGTGATTTGTTTGCAAACTTCTGGAAAAGGTCTCATATTTATAGTTGGCAGGCAAATGTTTAGAAGAAAAGTAAACAG 5850

----:----|----:----|----:----|----:----|----:----|----:----|----:----|----:----|----:----|----:----|----:----|----:----|----:----|----:----|----:----|

K C L T T Q R F A A L * I E S E H S L S F F K Y F L L K R * E G T H L * L H * C * F S S C I L V S G F1

N V * Q H N V L Q P Y E S N L N T L S P F S N T F S * S D E K V H I C D C T D A D S L P V F * Y L E F2

M F N N T T F C S L M N R I * T L S L L F Q I L S L E A M R R Y T S V T A L M L I L F L Y S S I W N F3

5851 AAATGTTTAACAACACAACGTTTTGCAGCCTTATGAATCGAATCTGAACACTCTCTCTCCTTTTTCAAATACTTTCTCTTGAAGCGATGAGAAGGTACACATCTGTGACTGCACTGATGCTGATTCTCTTCCTGTATTCTAGTATCTGGA 6000

----:----|----:----|----:----|----:----|----:----|----:----|----:----|----:----|----:----|----:----|----:----|----:----|----:----|----:----|----:----|

I S P A T Y E * * G V Q K N G G S C K R F C I * L R T E A S V V L K A K I V V G H K L C E * I N R * F1

S V R P L M N D E E F K R M E G L A K D F A F N L G P R L Q W Y L K L K S W W A T N Y V S R S T D D F2

Q S G H L * M M R S S K E W R V L Q K I L H L T * D R G F S G T * S * N R G G P Q T M * V D Q Q M T F3

6001 ATCAGTCCGGCCACTTATGAATGATGAGGAGTTCAAAAGAATGGAGGGTCTTGCAAAAGATTTTGCATTTAACTTAGGACCGAGGCTTCAGTGGTACTTAAAGCTAAAATCGTGGTGGGCCACAAACTATGTGAGTAGATCAACAGATGA 6150

----:----|----:----|----:----|----:----|----:----|----:----|----:----|----:----|----:----|----:----|----:----|----:----|----:----|----:----|----:----|

L K Y S T D V I * F V P C C V F C L M F I L S * M E K K G I E T C A K C I G D R L I C T E I S * C G F1

L N I Q Q M * F D L F P V V F F A * C L F S L K W R R R E L K P V P S A L A T A * F A Q K * V D V G F2

* I F N R C N L I C S L L C F L L N V Y S L L N G E E G N * N L C Q V H W R P L D L H R N K L M W D F3

6151 CTTAAATATTCAACAGATGTAATTTGATTTGTTCCCTGTTGTGTTTTTTGCTTAATGTTTATTCTCTCTTAAATGGAGAAGAAGGGAATTGAAACCTGTGCCAAGTGCATTGGCGACCGCTTGATTTGCACAGAAATAAGTTGATGTGGG 6300

----:----|----:----|----:----|----:----|----:----|----:----|----:----|----:----|----:----|----:----|----:----|----:----|----:----|----:----|----:----|

I L K T E N L V N E T L K S T S L F S S V F I I L E S A I * L L R L L L G Y L C L F L * L N P L G S F1

F * K Q K I W * M K L * R A H L C S V L C S L F W S Q Q F S Y Y D C S * A T Y A C F Y N * T L * V A F2

F E N R K F G K * N F K E H I F V Q F C V H Y S G V S N L V I T T A L R L L M L V F I I K P F R * L F3

6301 ATTTTGAAAACAGAAAATTTGGTAAATGAAACTTTAAAGAGCACATCTTTGTTCAGTTCTGTGTTCATTATTCTGGAGTCAGCAATTTAGTTATTACGACTGCTCTTAGGCTACTTATGCTTGTTTTTATAATTAAACCCTTTAGGTAGC 6450

----:----|----:----|----:----|----:----|----:----|----:----|----:----|----:----|----:----|----:----|----:----|----:----|----:----|----:----|----:----|

C P G S L D R R M L * G N C F M * N * * S I W K R N K Q L * Q M F F T R K K I Q T S V S * * E L C N F1

V Q V V * I E E C F R E I V L C R T D K A Y G K E I N N C N K C F S P E R K S K L Q Y R S E N C V M F2

S R * F R * K N A L G K L F Y V E L I K H M E K K * T I V T N V F H Q K E N P N F S I V V R T V * C F3

6451 TGTCCAGGTAGTTTAGATAGAAGAATGCTTTAGGGAAATTGTTTTATGTAGAACTGATAAAGCATATGGAAAAGAAATAAACAATTGTAACAAATGTTTTTCACCAGAAAGAAAATCCAAACTTCAGTATCGTAGTGAGAACTGTGTAAT 6600

----:----|----:----|----:----|----:----|----:----|----:----|----:----|----:----|----:----|----:----|----:----|----:----|----:----|----:----|----:----|

A I R I V E W C G L E S T L R R T S A S F N S P A V G R V A N H * I K L L T A P H P T W P Q K Q L W F1

Q L E S * N G V G * K V P * G G L Q L V S T P L Q * A E * P T T R S S C L Q P L I Q P G L K N S C G F2

N * N R R M V W V R K Y L K E D F S * F Q L P C S R Q S S Q P L D Q V A Y S P S S N L A S K T A V D F3

6601 GCAATTAGAATCGTAGAATGGTGTGGGTTAGAAAGTACCTTAAGGAGGACTTCAGCTAGTTTCAACTCCCCTGCAGTAGGCAGAGTAGCCAACCACTAGATCAAGTTGCTTACAGCCCCTCATCCAACCTGGCCTCAAAAACAGCTGTGG 6750

----:----|----:----|----:----|----:----|----:----|----:----|----:----|----:----|----:----|----:----|----:----|----:----|----:----|----:----|----:----|

M G H P R L L W E T C S S A S L P S * * R I S S S Y I * L K S P L F * V K V I N P C S I L L C L F T F1

W G I H D F S G K L V P V P R C P H N E E F L P H I S N * N L P S F R L K S L T L V L S F Y A F L Q F2

G A S T T S L G N L F Q C L A A L I M K N F F L I Y L T E I S P L L G * S H * P L F Y P F M P F Y K F3

6751 ATGGGGCATCCACGACTTCTCTGGGAAACTTGTTCCAGTGCCTCGCTGCCCTCATAATGAAGAATTTCTTCCTCATATATCTAACTGAAATCTCCCCTCTTTTAGGTTAAAGTCATTAACCCTTGTTCTATCCTTTTATGCCTTTTTACA 6900

----:----|----:----|----:----|----:----|----:----|----:----|----:----|----:----|----:----|----:----|----:----|----:----|----:----|----:----|----:----|

S P S P T F L * A P S G Y R K V A I S S A Q S L F F S R L N S P K A A L Q L P D V V S K I Y L Y T L F1

V P L Q L S C R P P L G T G R L R * V L L R A F S S P G * T A P R L P F S F L M * Y L K F I S T R * F2

S L S N F P V G P L W V Q E G C D K F C S E P F L L Q A E Q P Q G C P S A S * C S I * N L S L H V N F3

6901 AGTCCCTCTCCAACTTTCCTGTAGGCCCCCTCTGGGTACAGGAAGGTTGCGATAAGTTCTGCTCAGAGCCTTTTCTTCTCCAGGCTGAACAGCCCCAAGGCTGCCCTTCAGCTTCCTGATGTAGTATCTAAAATTTATCTCTACACGTTA 7050

----:----|----:----|----:----|----:----|----:----|----:----|----:----|----:----|----:----|----:----|----:----|----:----|----:----|----:----|----:----|

T V V L L G Y P S I W D F * C I L S V I * R * Q L I Q K L T V T R I K L S H S L L K F A F P E Y Q Q F1

L W F S * V T P A F G I S D A F * V L Y E D D S * S R N L L L P E L N Y L T L Y * S L L S L N T N R F2

C G S L R L P Q H L G F L M H F K C Y M K M T A N P E T Y C Y Q N * I I S L F I E V C F P * I P T E F3

7051 ACTGTGGTTCTCTTAGGTTACCCCAGCATTTGGGATTTCTGATGCATTTTAAGTGTTATATGAAGATGACAGCTAATCCAGAAACTTACTGTTACCAGAATTAAATTATCTCACTCTTTATTGAAGTTTGCTTTCCCTGAATACCAACAG 7200

----:----|----:----|----:----|----:----|----:----|----:----|----:----|----:----|----:----|----:----|----:----|----:----|----:----|----:----|----:----|

K S V E D F C F F L C F * V * G Q A D C F L P I V W S V V L W N Y * H S R Y Q L * K * * T F T H F F F1

N Q W R I F V S F Y V F K C R G K L T V F F L L C G V L Y C G I T N I A G I N Y R N N E L L L I F F F2

I S G G F L F L S M F L S V G A S * L F S S Y C V E C C I V E L L T * Q V S T I E I M N F Y S F F S F3

7201 AAATCAGTGGAGGATTTTTGTTTCTTTCTATGTTTTTAAGTGTAGGGGCAAGCTGACTGTTTTCTTCCTATTGTGTGGAGTGTTGTATTGTGGAATTACTAACATAGCAGGTATCAACTATAGAAATAATGAACTTTTACTCATTTTTTT 7350

----:----|----:----|----:----|----:----|----:----|----:----|----:----|----:----|----:----|----:----|----:----|----:----|----:----|----:----|----:----|

H P T G E * L V G R V Y L P * R A W T N N G * Q * L F C N G R * M L * I P Y E S K K H G K I S A M N F1

I L Q V S D W W E E Y I Y L R G R G P I M V N S N Y F A M V G K C Y K F L M S Q R S M V K F L P * I F2

S Y R * V I G G K S I S T L E G V D Q * W L T V T I L Q W * V N A I N S L * V K E A W * N F C H E S F3

7351 CATCCTACAGGTGAGTGATTGGTGGGAAGAGTATATCTACCTTAGAGGGCGTGGACCAATAATGGTTAACAGTAACTATTTTGCAATGGTAGGTAAATGCTATAAATTCCTTATGAGTCAAAGAAGCATGGTAAAATTTCTGCCATGAAT 7500

----:----|----:----|----:----|----:----|----:----|----:----|----:----|----:----|----:----|----:----|----:----|----:----|----:----|----:----|----:----|

L N L F F F N V F L Y R P P L P L S I W L S F F L S * I * S E T N V L * S A I F L I S I V H V S V R F1

L I Y F S S M F S F I D P H C H * V Y G F L F F F L E Y N L K Q M S Y K V L S S * * A * Y M C Q S G F2

* F I F L Q C F P L * T P I A T E Y M A F F F S F L N I I * N K C L I K C Y L L N K H S T C V S Q A F3

7501 CTTAATTTATTTTTCTTCAATGTTTTCCTTTATAGACCCCCATTGCCACTGAGTATATGGCTTTCTTTTTTTCTTTCTTGAATATAATCTGAAACAAATGTCTTATAAAGTGCTATCTTCTTAATAAGCATAGTACATGTGTCAGTCAGG 7650

----:----|----:----|----:----|----:----|----:----|----:----|----:----|----:----|----:----|----:----|----:----|----:----|----:----|----:----|----:----|

Q T C A V M A S F T V L C L L Q Q A V F K K I H V K K L M H S T V V C F D I C K C L Y R R * N C E R F1

K L V Q * W L L L Q F Y A Y C N K Q S L K R F M * K N * C I A Q L C V L I F V N A Y T G G K I V K D F2

N L C S D G F F Y S S M L T A T S S L * K D S C E K T D A * H S C V F * Y L * M P I Q E V K L * K T F3

7651 CAAACTTGTGCAGTGATGGCTTCTTTTACAGTTCTATGCTTACTGCAACAAGCAGTCTTTAAAAAGATTCATGTGAAAAAACTGATGCATAGCACAGTTGTGTGTTTTGATATTTGTAAATGCCTATACAGGAGGTAAAATTGTGAAAGA 7800

----:----|----:----|----:----|----:----|----:----|----:----|----:----|----:----|----:----|----:----|----:----|----:----|----:----|----:----|----:----|

P * G * R R K * E I L W F * I Q V F S S I I W D D * W L C C R F S D W F C L K K L W L L K K W N * S F1

P E V K G V S R K Y C G F E F K F S V A L Y G M T N G Y A V G F Q T G S V * R N C G Y * R N G I E V F2

L R L K A * V G N I V V L N S S F Q * H Y M G * L M A M L * V F R L V L F K E I V A I E E M E L K S F3

7801 CCCTGAGGTTAAAGGCGTAAGTAGGAAATATTGTGGTTTTGAATTCAAGTTTTCAGTAGCATTATATGGGATGACTAATGGCTATGCTGTAGGTTTTCAGACTGGTTCTGTTTAAAGAAATTGTGGCTATTGAAGAAATGGAATTGAAGT 7950

----:----|----:----|----:----|----:----|----:----|----:----|----:----|----:----|----:----|----:----|----:----|----:----|----:----|----:----|----:----|

H R L T V L V Y F L Y C Q N L A L L T R I * L K K K K K A N T Q K T S S T V Y F S V * Y G N S * L K F1

I D S L Y W C T S C I V K T W L Y * Q E Y S L K K K K K Q T H K K P V V L C I F Q C N M V I A N L R F2

S T H C T G V L P V L S K L G F I D K N I A * K K K K S K H T K N Q * Y C V F F S V I W * * L T * D F3

7951 CATCGACTCACTGTACTGGTGTACTTCCTGTATTGTCAAAACTTGGCTTTATTGACAAGAATATAGCTTAAAAAAAAAAAAAAAGCAAACACACAAAAAACCAGTAGTACTGTGTATTTTTCAGTGTAATATGGTAATAGCTAACTTAAG 8100

----:----|----:----|----:----|----:----|----:----|----:----|----:----|----:----|----:----|----:----|----:----|----:----|----:----|----:----|----:----|

I L I Y F A G L P S F I S H N H T G S * S W * Y Y P C H P A L P E K T G Q T R N Q A S M Y I G L N S F1

F L F T L Q D F L H L S P T T I Q A A R A G N I I H A I L L Y R K K L D R Q E I K P V C I L V * I V F2

S Y L L C R T S F I Y L P Q P Y R Q L E L V I L S M P S C S T G K N W T D K K S S Q Y V Y W F K * F F3

8101 ATTCTTATTTACTTTGCAGGACTTCCTTCATTTATCTCCCACAACCATACAGGCAGCTAGAGCTGGTAATATTATCCATGCCATCCTGCTCTACCGGAAAAAACTGGACAGACAAGAAATCAAGCCAGTATGTATATTGGTTTAAATAGT 8250

----:----|----:----|----:----|----:----|----:----|----:----|----:----|----:----|----:----|----:----|----:----|----:----|----:----|----:----|----:----|

F * S F P P Q F V R S * S A F R A G E Q C A V P E P Y E R C N * I C D L R H F C Y H L N C T C A Q T F1

S D L F H H S L * E V D Q L L E Q E N N V L C Q N H M R D A T K F V I S G I F V I T * I A H V H K L F2

L I F S T T V C K K L I S F * S R R T M C C A R T I * E M Q L N L * S Q A F L L S L E L H M C T N Y F3

8251 TTCTGATCTTTTCCACCACAGTTTGTAAGAAGTTGATCAGCTTTTAGAGCAGGAGAACAATGTGCTGTGCCAGAACCATATGAGAGATGCAACTAAATTTGTGATCTCAGGCATTTTTGTTATCACTTGAATTGCACATGTGCACAAACT 8400

----:----|----:----|----:----|----:----|----:----|----:----|----:----|----:----|----:----|----:----|----:----|----:----|----:----|----:----|----:----|

T I R Y R L L L H Q L I A A L E T D V S I C P K E T * C L V L A Y G K Y T T * Q I I D T Y Q A C H G F1

L S D T G F C Y T S * L Q H L K L T Y L F A Q K R H N V L C L H M V N I P P N K L L I R T K H V M V F2

Y Q I Q A S V T P A N C S T * N * R I Y L P K R D I M S C A C I W * I Y H L T N Y * Y V P S M S W Y F3

8401 ACTATCAGATACAGGCTTCTGTTACACCAGCTAATTGCAGCACTTGAAACTGACGTATCTATTTGCCCAAAAGAGACATAATGTCTTGTGCTTGCATATGGTAAATATACCACCTAACAAATTATTGATACGTACCAAGCATGTCATGGT 8550

----:----|----:----|----:----|----:----|----:----|----:----|----:----|----:----|----:----|----:----|----:----|----:----|----:----|----:----|----:----|

T F E P P T S E N P S R K I I F L F P A K T V C K D E F * I E * H V K * V A C * T I N Y N * M C T A F1

L L N H R L L K I P A E K L F S Y F L L K L S V R M N F K L S S M * N K L H A E R * T T I E C V L L F2

F * T T D F * K S Q Q K N Y F L I S C * N C L * G * I L N * V A C E I S C M L N D K L Q L N V Y C L F3

8551 ACTTTTGAACCACCGACTTCTGAAAATCCCAGCAGAAAAATTATTTTCTTATTTCCTGCTAAAACTGTCTGTAAGGATGAATTTTAAATTGAGTAGCATGTGAAATAAGTTGCATGCTGAACGATAAACTACAATTGAATGTGTACTGCT 8700

----:----|----:----|----:----|----:----|----:----|----:----|----:----|----:----|----:----|----:----|----:----|----:----|----:----|----:----|----:----|

C Y V F M L F F M I S A Y S C F A Y * I C Q M Q * I Y L L D I * R W K T K T A V Y C C I Y S F L N L F1

A M F S C C S L * F L L T V A L L T E S A K C S E F I Y L I Y E G G K Q K L Q Y I A A F T R F * T W F2

L C F H V V L Y D F C L Q L L C L L N L P N A V N L F T * Y M K V E N K N C S I L L H L L V S E P G F3

8701 TGCTATGTTTTCATGTTGTTCTTTATGATTTCTGCTTACAGTTGCTTTGCTTACTGAATCTGCCAAATGCAGTGAATTTATTTACTTGATATATGAAGGTGGAAAACAAAAACTGCAGTATATTGCTGCATTTACTCGTTTCTGAACCTG 8850

----:----|----:----|----:----|----:----|----:----|----:----|----:----|----:----|----:----|----:----|----:----|----:----|----:----|----:----|----:----|

V * Q * S * S T F K K K L N L T E K K F R N T * F T L C V T S H F L R T L H F Q K T * V S F K Q V M F1

C S N K A S Q L L K K S * I * L K R S L E T L D L H C V L L H T F S G H Y I S R K L K C L S S R * C F2

V A I K L V N F * K K V K F N * K E V * K H L I Y T V C Y F T L S Q D T T F P E N L S V F Q A G N V F3

8851 GTGTAGCAATAAAGCTAGTCAACTTTTAAAAAAAAGTTAAATTTAACTGAAAAGAAGTTTAGAAACACTTGATTTACACTGTGTGTTACTTCACACTTTCTCAGGACACTACATTTCCAGAAAACTTAAGTGTCTTTCAAGCAGGTAATG 9000

----:----|----:----|----:----|----:----|----:----|----:----|----:----|----:----|----:----|----:----|----:----|----:----|----:----|----:----|----:----|

F E C * V C F Y F I F Y S S K H H N P Q P I Y L V C D E S V D A V F F L V A Y K E T L R N V Y * I V F1

S N A R F V S I S Y F I Q V N I I T P S P Y T * S V M S Q W M Q F F S * W H I K K H * E M F T K L * F2

R M L G L F L F H I L F K * T S * P P A H I L S L * * V S G C S F F P S G I * R N T K K C L L N C S F3

9001 TTCGAATGCTAGGTTTGTTTCTATTTCATATTTTATTCAAGTAAACATCATAACCCCCAGCCCATATACTTAGTCTGTGATGAGTCAGTGGATGCAGTTTTTTTCCTAGTGGCATATAAAGAAACACTAAGAAATGTTTACTAAATTGTA 9150

----:----|----:----|----:----|----:----|----:----|----:----|----:----|----:----|----:----|----:----|----:----|----:----|----:----|----:----|----:----|

V F L Q S E C P R E V F L L F C L F G C D P D N W S K Y I V C L R N H I W Q A E H C I K N I Q T S E F1

F F F K A S V L G K S F Y C S V C L D V T Q I I G A N I * C V * E I I F G R Q N T A L R T S R L Q R F2

F S S K R V S * G S L S T V L F V W M * P R * L E Q I Y S V S K K S Y L A G R T L H * E H P D F R G F3

9151 GTTTTTCTTCAAAGCGAGTGTCCTAGGGAAGTCTTTCTACTGTTCTGTTTGTTTGGATGTGACCCAGATAATTGGAGCAAATATATAGTGTGTCTAAGAAATCATATTTGGCAGGCAGAACACTGCATTAAGAACATCCAGACTTCAGAG 9300

----:----|----:----|----:----|----:----|----:----|----:----|----:----|----:----|----:----|----:----|----:----|----:----|----:----|----:----|----:----|

E F R S * Y G H S G C K K V C F E N K G H H W K R A V Q * L L L F E A K V V L G L G V V I S P A H * F1

N S D P D M D I V V V R K S A L K T K D T I G R E Q F S S C Y C L K Q R L S L D L V L * F L L H I D F2

I Q I L I W T * W L * E S L L * K Q R T P L E E S S S V A V T V * S K G C P W T W C C D F S C T L T F3

9301 GAATTCAGATCCTGATATGGACATAGTGGTTGTAAGAAAGTCTGCTTTGAAAACAAAGGACACCATTGGAAGAGAGCAGTTCAGTAGCTGTTACTGTTTGAAGCAAAGGTTGTCCTTGGACTTGGTGTTGTGATTTCTCCTGCACATTGA 9450

----:----|----:----|----:----|----:----|----:----|----:----|----:----|----:----|----:----|----:----|----:----|----:----|----:----|----:----|----:----|

L H C A V L * I F G L * Y K A G S Q S R * E R V G G E L C T F L G L * C S F I A R L T R N K R R N Q F1

F T V R F F E Y L G F D T R Q G A K V D K K G * E E S C V L S W V Y D V V S * L D * P G T K E E I K F2

S L C G S L N I W A L I Q G R E P K * I R K G R R R V V Y F L G F M M * F H S * I N Q E Q K K K S R F3

9451 CTTCACTGTGCGGTTCTTTGAATATTTGGGCTTTGATACAAGGCAGGGAGCCAAAGTAGATAAGAAAGGGTAGGAGGAGAGTTGTGTACTTTCTTGGGTTTATGATGTAGTTTCATAGCTAGATTAACCAGGAACAAAAGAAGAAATCAA 9600

----:----|----:----|----:----|----:----|----:----|----:----|----:----|----:----|----:----|----:----|----:----|----:----|----:----|----:----|----:----|

G V T Q I D H R A H W I S D A R Y L Q C L L L N G E L * * E E K N A A T G M S V K S F T C S S R E S F1

G * P R L T I E H I G F L M Q G I F S A C S * M V S F D K R K K M Q L L E C Q * N L S R V V Q G K A F2

G N P D * P * S T L D F * C K V S S V L A P E W * A L I R G K K C S Y W N V S E I F H V * F K G K Q F3

9601 GGGGTAACCCAGATTGACCATAGAGCACATTGGATTTCTGATGCAAGGTATCTTCAGTGCTTGCTCCTGAATGGTGAGCTTTGATAAGAGGAAAAAAATGCAGCTACTGGAATGTCAGTGAAATCTTTCACGTGTAGTTCAAGGGAAAGC 9750

----:----|----:----|----:----|----:----|----:----|----:----|----:----|----:----|----:----|----:----|----:----|----:----|----:----|----:----|----:----|

K E Q G K K K K R C I R N I I V L Q P L F N R T V F S F * F T N G I C * I R I * S T C F V Q I L L M F1

R N R G K K K K D A L E I L L F C S L C L T E L F F L F N L L M V Y A K S E F N L H V L Y R F F * W F2

G T G E K K K K M H * K Y Y C S A A S V * P N C F F F L I Y * W Y M L N Q N L I Y M F C T D S S D G F3

9751 AAGGAACAGGGGAAAAAAAAAAAAAGATGCATTAGAAATATTATTGTTCTGCAGCCTCTGTTTAACCGAACTGTTTTTTCTTTTTAATTTACTAATGGTATATGCTAAATCAGAATTTAATCTACATGTTTTGTACAGATTCTTCTGATG 9900

----:----|----:----|----:----|----:----|----:----|----:----|----:----|----:----|----:----|----:----|----:----|----:----|----:----|----:----|----:----|

G S T V P L C S A Q W E R M F N T S R I P G E E S G K Q S S S R R E T S E W N S Q C W G Y F L L E S F1

D L L F H F A Q L S G S G C L I P P A S Q E K N Q V S R A V Q G E K H L N G I H S V G A I F C * S H F2

I Y C S T L L S S V G A D V * Y L P H P R R R I R * A E Q F K E R N I * M E F T V L G L F S A R V T F3

9901 GGATCTACTGTTCCACTTTGCTCAGCTCAGTGGGAGCGGATGTTTAATACCTCCCGCATCCCAGGAGAAGAATCAGGTAAGCAGAGCAGTTCAAGGAGAGAAACATCTGAATGGAATTCACAGTGTTGGGGCTATTTTCTGCTAGAGTCA 10050

----:----|----:----|----:----|----:----|----:----|----:----|----:----|----:----|----:----|----:----|----:----|----:----|----:----|----:----|----:----|

L F P S * G V E R H Q G T N T A V S R E V M N C H S T * I V F A V Q N D F E I S L Q C L C L * L K A F1

F S P A K G L K D T R A P I L L F L G K * * I A I A R R * F L L F R M T L K F H F S V C V F N * K L F2

F P Q L R G * K T P G H Q Y C C F S G S N E L P * H V D S F C C S E * L * N F T S V F V S L I K S * F3

10051 CTTTTCCCCAGCTAAGGGGTTGAAAGACACCAGGGCACCAATACTGCTGTTTCTCGGGAAGTAATGAATTGCCATAGCACGTAGATAGTTTTTGCTGTTCAGAATGACTTTGAAATTTCACTTCAGTGTTTGTGTCTTTAATTAAAAGCT 10200

----:----|----:----|----:----|----:----|----:----|----:----|----:----|----:----|----:----|----:----|----:----|----:----|----:----|----:----|----:----|

D F R K V S A L C F * S I C N L Y F F L L F Y S F R F L L I H V V F L C E D R V F L S K T F * N L S F1

T S G K C L P C A F N L Y V T F T F F Y C S T L S G F F * Y M W F F F V K I E F S Y L R H F K I C P F2

L Q E S V C P V L L I Y M * P L L F F T V L L F Q V S F D T C G F S L * R S S F L I * D I L K F V L F3

10201 GACTTCAGGAAAGTGTCTGCCCTGTGCTTTTAATCTATATGTAACCTTTACTTTTTTTTACTGTTCTACTCTTTCAGGTTTCTTTTGATACATGTGGTTTTTCTTTGTGAAGATCGAGTTTTCTTATCTAAGACATTTTAAAATTTGTCC 10350

----:----|----:----|----:----|----:----|----:----|----:----|----:----|----:----|----:----|----:----|----:----|----:----|----:----|----:----|----:----|

C F D F F S F C L S F T T A S * Y I L L N M N V C S A L L L T * V K G R Y N L I P G R L F S T S V I F1

V L T F F L S V Y R L Q Q H P N I F F S I * M Y V Q H C C L P R L K A G I I L F L A G F S A P A L S F2

F * L F F F L F I V Y N S I L I Y S S Q Y E C M F S T A A Y L G * R Q V * S Y S W Q A F Q H Q R Y L F3

10351 TGTTTTGACTTTTTTTCTTTCTGTTTATCGTTTACAACAGCATCCTAATATATTCTTCTCAATATGAATGTATGTTCAGCACTGCTGCTTACCTAGGTTAAAGGCAGGTATAATCTTATTCCTGGCAGGCTTTTCAGCACCAGCGTTATC 10500

----:----|----:----|----:----|----:----|----:----|----:----|----:----|----:----|----:----|----:----|----:----|----:----|----:----|----:----|----:----|

L M F C M C C K I P G R * I S C R K D S D * I F L C N T L R K C M L F L L A P A L T P V N E L I I L F1

* C F V C A V R Y Q V D E S A V G R T V T K S F S V T H * E N V C F F Y * L L L * L P S M N * L F * F2

D V L Y V L * D T R * M N Q L * E G Q * L N L S L * H I K K M Y A F F I S S C S N S R Q * T D Y S K F3

10501 TTGATGTTTTGTATGTGCTGTAAGATACCAGGTAGATGAATCAGCTGTAGGAAGGACAGTGACTAAATCTTTCTCTGTAACACATTAAGAAAATGTATGCTTTTTTTATTAGCTCCTGCTCTAACTCCCGTCAATGAACTGATTATTCTA 10650

----:----|----:----|----:----|----:----|----:----|----:----|----:----|----:----|----:----|----:----|----:----|----:----|----:----|----:----|----:----|

N S E * L N L F I L L L N R G R Y L F K Y N R L G F F V * I C S C S F C G T L R V C L Q A I M L A L F1

I L S N * I F S S Y C * I E G G T C L S T T G W V F L C E S V P A A S V E H * E C V Y K Q S C * P C F2

F * V T K S F H L T V E * R E V P V * V Q Q V G F F C V N L F L Q L L W N T K S V F T S N H V S P A F3

10651 AATTCTGAGTAACTAAATCTTTTCATCTTACTGTTGAATAGAGGGAGGTACCTGTTTAAGTACAACAGGTTGGGTTTTTTTGTGTGAATCTGTTCCTGCAGCTTCTGTGGAACACTAAGAGTGTGTTTACAAGCAATCATGTTAGCCCTG 10800

----:----|----:----|----:----|----:----|----:----|----:----|----:----|----:----|----:----|----:----|----:----|----:----|----:----|----:----|----:----|

P K K * L Y L L V I V H L Y I F T V N V H R L S I Q T T G V N Y V L K E G R C M H S L T L P I G R K F1

P K N N C I C * S * F I C T F S L * M Y I G F Q F R Q Q E L I T S * R K A D A C I H * H Y L * V E K F2

Q K I I V S V S H S S S V H F H C K C T * A F N S D N R S * L R L E G R Q M H A F I N I T Y R * K S F3

10801 CCCAAAAAATAATTGTATCTGTTAGTCATAGTTCATCTGTACATTTTCACTGTAAATGTACATAGGCTTTCAATTCAGACAACAGGAGTTAATTACGTCTTGAAGGAAGGCAGATGCATGCATTCATTAACATTACCTATAGGTAGAAAA 10950

----:----|----:----|----:----|----:----|----:----|----:----|----:----|----:----|----:----|----:----|----:----|----:----|----:----|----:----|----:----|

V G Y V T S L F Q S F Q L L V E V S H I T T G * F H L L I L F F N A F S G R L H Y I S M V E L E Y P F1

* G M S H L C F S P F S C L L K L A I * L R G N F I S L F Y F L M P S L A D Y I T Y P W * S W S T L F2

R V C H I S V S V L S V A C * S * P Y N Y G V I S S P Y F I F * C L L W Q I T L H I H G R V G V P F F3

10951 GTAGGGTATGTCACATCTCTGTTTCAGTCCTTTCAGTTGCTTGTTGAAGTTAGCCATATAACTACGGGGTAATTTCATCTCCTTATTTTATTTTTTAATGCCTTCTCTGGCAGATTACATTACATATCCATGGTAGAGTTGGAGTACCCT 11100

----:----|----:----|----:----|----:----|----:----|----:----|----:----|----:----|----:----|----:----|----:----|----:----|----:----|----:----|----:----|

F Q V T V M F F S S V M K S Q N G L S L K S Y * R S C S S N P C H G Q G T F Q I P S H P P A W T D I F1

S K * Q * C S L V V S * S H R M V * V * R A I K D H V V P T P A M D R A P S R Y L P I P L P G Q I F F2

P S D S D V L * * C H E V T E W F E F E E L L K I M * F Q P L P W T G H L P D T F P S P C L D R Y S F3

11101 TTCCAAGTGACAGTGATGTTCTTTAGTAGTGTCATGAAGTCACAGAATGGTTTGAGTTTGAAGAGCTATTAAAGATCATGTAGTTCCAACCCCTGCCATGGACAGGGCACCTTCCAGATACCTTCCCATCCCCCTGCCTGGACAGATATT 11250

----:----|----:----|----:----|----:----|----:----|----:----|----:----|----:----|----:----|----:----|----:----|----:----|----:----|----:----|----:----|

P D L P A A S P V I H L R W C E P * * L E M L T L V V N R * K K M F L S I F L I F E N Q T L Y * L C F1

Q I C Q L L L L * F I C A G V S L S N W K C * H * * S T D E R K C F Y P F F S F L R I K R C T N C A F2

R F A S C F S C N S F A L V * A L V T G N A N I S S Q Q M K E N V S I H F S H F * E S N A V L I V Q F3

11251 CCAGATTTGCCAGCTGCTTCTCCTGTAATTCATTTGCGCTGGTGTGAGCCTTAGTAACTGGAAATGCTAACATTAGTAGTCAACAGATGAAAGAAAATGTTTCTATCCATTTTTCTCATTTTTGAGAATCAAACGCTGTACTAATTGTGC 11400

----:----|----:----|----:----|----:----|----:----|----:----|----:----|----:----|----:----|----:----|----:----|----:----|----:----|----:----|----:----|

S R F S * S F Q V P L * Y L L Q K L E L Y V G * P K V P * V * N C F * S V D T S L N L M D R S E H P F1

A D F L R A F R C H C D T C Y K S L S F M * A S Q R C H K Y K T A S D Q W I P P * T L W I G Q N T Q F2

Q I F L E L S G A I V I L A T K A * A L C R L A K G A I S I K L L L I S G Y L P K P Y G * V R T P K F3

11401 AGCAGATTTTCTTAGAGCTTTCAGGTGCCATTGTGATACTTGCTACAAAAGCTTGAGCTTTATGTAGGCTAGCCAAAGGTGCCATAAGTATAAAACTGCTTCTGATCAGTGGATACCTCCCTAAACCTTATGGATAGGTCAGAACACCCA 11550

----:----|----:----|----:----|----:----|----:----|----:----|----:----|----:----|----:----|----:----|----:----|----:----|----:----|----:----|----:----|

K Q R T L S W Y C V S Q N N E C * A F R M S L S S L S L R M S A V L W K S E R T E L L V S I I * G V F1

S R E L S P G T V F L R I M N V R L L G C L F L H C L * E C Q Q F F G R A K E L N Y W F P S Y E E Y F2

A E N S L L V L C F S E * * M L G F * D V S F F T V S K N V S S S L E E R K N * I T G F H H M R S I F3

11551 AAGCAGAGAACTCTCTCCTGGTACTGTGTTTCTCAGAATAATGAATGTTAGGCTTTTAGGATGTCTCTTTCTTCACTGTCTCTAAGAATGTCAGCAGTTCTTTGGAAGAGCGAAAGAACTGAATTACTGGTTTCCATCATATGAGGAGTA 11700

----:----|----:----|----:----|----:----|----:----|----:----|----:----|----:----|----:----|----:----|----:----|----:----|----:----|----:----|----:----|

Y L A A * K A S L G V * H K A F M S * L * A Y S S * P S V Y K R S E D K L I * M S C G * L I V H A V F1

T * L L R K L H * V F D T K P S C H N F R L I P V D L L F I N E V R I N S F K * A V A N * * Y M Q S F2

P S C L E S F T R C L T Q S L H V I T L G L F Q L T F C L * T K * G * T H L N E L W L I D S T C S R F3

11701 TACCTAGCTGCTTAGAAAGCTTCACTAGGTGTTTGACACAAAGCCTTCATGTCATAACTTTAGGCTTATTCCAGTTGACCTTCTGTTTATAAACGAAGTGAGGATAAACTCATTTAAATGAGCTGTGGCTAATTGATAGTACATGCAGTC 11850

----:----|----:----|----:----|----:----|----:----|----:----|----:----|----:----|----:----|----:----|----:----|----:----|----:----|----:----|----:----|

G T * H Y I V Y Y Y Y L * T S A L F S * K Q F K I L D T L Q H V K D S K H I V V Y H K G R Y F K V W F1

A L D I I * C I I T I C R L Q L C F H K S N L K F * I L S S T * K T A N T L L S I T R G V T S K Y G F2

H L T L Y S V L L L F V D F S F V F I K A I * N S R Y S P A R E R Q Q T H C C L S Q G A L L Q S M A F3

11851 GGCACTTGACATTATATAGTGTATTATTACTATTTGTAGACTTCAGCTTTGTTTTCATAAAAGCAATTTAAAATTCTAGATACTCTCCAGCACGTGAAAGACAGCAAACACATTGTTGTCTATCACAAGGGGCGTTACTTCAAAGTATGG 12000

----:----|----:----|----:----|----:----|----:----|----:----|----:----|----:----|----:----|----:----|----:----|----:----|----:----|----:----|----:----|

L Y H D G R L L K P R E I E Q Q I Q R I L D D D S E P Q A G E E K L A A L T A G D R Y G Y Y H F S N F1

C T M M A D C * N L E R L N S R Y R E F L M M I Q S L R L V K R N * Q L L L Q E I G M A I I I F Q I F2

V P * W Q T V E T S R D * T A D T E N S * * * F R A S G W * R E I S S S Y C R R * V W L L S F F K L F3

12001 CTGTACCATGATGGCAGACTGTTGAAACCTCGAGAGATTGAACAGCAGATACAGAGAATTCTTGATGATGATTCAGAGCCTCAGGCTGGTGAAGAGAAATTAGCAGCTCTTACTGCAGGAGATAGGTATGGCTATTATCATTTTTCAAAT 12150

----:----|----:----|----:----|----:----|----:----|----:----|----:----|----:----|----:----|----:----|----:----|----:----|----:----|----:----|----:----|

* F H N L E V Y C * H I T A V L S F V M M M W N * K * F C N C S S V * T P H T E L E S * G S L L Y L F1

N F I I * K Y I A D I L L L C C P L * * * C G I K N N F A I A A L Y K L P I L S W R A R E V Y F I C F2

I S * F R S I L L T Y Y C C A V L C N D D V E L K I I L Q L Q L C I N S P Y * A G E L G K F T L F V F3

12151 TAATTTCATAATTTAGAAGTATATTGCTGACATATTACTGCTGTGCTGTCCTTTGTAATGATGATGTGGAATTAAAAATAATTTTGCAATTGCAGCTCTGTATAAACTCCCCATACTGAGCTGGAGAGCTAGGGAAGTTTACTTTATTTG 12300

----:----|----:----|----:----|----:----|----:----|----:----|----:----|----:----|----:----|----:----|----:----|----:----|----:----|----:----|----:----|

W * E V * F E A L Q * * A C * T M * A L D F L * G S L V F * E Y T Q T S E * E C K F S I C Y C R I K F1

G K R C N L R L C S S K H V K L C K L * I F S E V H W Y F K N I L K L Q S E N V N S A S A T A E * S F2

V R G V I * G F A V V S M L N Y V S F R F S L R F T G I L R I Y S N F R V R M * I Q H L L L Q N K A F3

12301 TGGTAAGAGGTGTAATTTGAGGCTTTGCAGTAGTAAGCATGTTAAACTATGTAAGCTTTAGATTTTCTCTGAGGTTCACTGGTATTTTAAGAATATACTCAAACTTCAGAGTGAGAATGTAAATTCAGCATCTGCTACTGCAGAATAAAG 12450

----:----|----:----|----:----|----:----|----:----|----:----|----:----|----:----|----:----|----:----|----:----|----:----|----:----|----:----|----:----|

Q Y L F T W L M V R D H * V Q F Y F D K F S S V L S D L F L D T E E K E L W V L M Y C L R D I V L * F1

S T Y S L G S W * E I I E F N S I L T N S V R S Y L I C F W T L K R K S Y G F * C T A * E I L Y F N F2

V L I H L A H G E R S L S S I L F * Q I Q F G L I * F V S G H * R E R A M G F N V L P K R Y C T L I F3

12451 CAGTACTTATTCACTTGGCTCATGGTGAGAGATCATTGAGTTCAATTCTATTTTGACAAATTCAGTTCGGTCTTATCTGATTTGTTTCTGGACACTGAAGAGAAAGAGCTATGGGTTTTAATGTACTGCCTAAGAGATATTGTACTTTAA 12600

----:----|----:----|----:----|----:----|----:----|----:----|----:----|----:----|----:----|----:----|----:----|----:----|----:----|----:----|----:----|

Y I F F I G K T Y K F L N K * M W H C Y I E I P L F V N F K S V Y H G S L P G E L S R C L Y R C Y S F1

T F F S L E K P T N F * I N K C G T A I * K S L C S L T L N L F T M E V F L E N C P D A Y I G V T A F2

H F F H W K N L Q I S K * I N V A L L Y R N P F V R * L * I C L P W K S S W R T V Q M L I * V L Q Q F3

12601 TACATTTTTTTCATTGGAAAAACCTACAAATTTCTAAATAAATAAATGTGGCACTGCTATATAGAAATCCCTTTGTTCGTTAACTTTAAATCTGTTTACCATGGAAGTCTTCCTGGAGAACTGTCCAGATGCTTATATAGGTGTTACAGC 12750

----:----|----:----|----:----|----:----|----:----|----:----|----:----|----:----|----:----|----:----|----:----|----:----|----:----|----:----|----:----|

N G Y F Y L * I V F I F M T I F F H I I G I F F C S V L K C R * S V L Y I L R A A D T S E E * R L C F1

M D I F I C E L F S F L * L F S F T * * V S S F V Q F L N V D D Q Y Y T Y C V Q Q T L L K S N A Y A F2

W I F L S V N C F H F Y D Y F L S H N R Y L L L F S S * M * M I S I I H I A C S R H F * R V T L M L F3

12751 AATGGATATTTTTATCTGTGAATTGTTTTCATTTTTATGACTATTTTCTTTCACATAATAGGTATCTTCTTTTGTTCAGTTCTTAAATGTAGATGATCAGTATTATACATATTGCGTGCAGCAGACACTTCTGAAGAGTAACGCTTATGC 12900

----:----|----:----|----:----|----:----|----:----|----:----|----:----|----:----|----:----|----:----|----:----|----:----|----:----|----:----|----:----|

F A F V L * Y D S I I E * F G L E G S * K F I * F Q L P C H G Q G Y F P L D Q V A Q R P S N L A L N F1

L H L C Y D M I A L L N S L D W K G A E S S F S S S S P A M D R V I F H * T R L L K G P P T W P * T F2

C I C V M I * * H Y * I V W I G R E L K V H L V P A P L P W T G L F S T R P G C S K A L Q P G P E H F3

12901 TTTGCATTTGTGTTATGATATGATAGCATTATTGAATAGTTTGGATTGGAAGGGAGCTGAAAGTTCATTTAGTTCCAGCTCCCCTGCCATGGACAGGGTTATTTTCCACTAGACCAGGTTGCTCAAAGGCCCTCCAACCTGGCCCTGAAC 13050

----:----|----:----|----:----|----:----|----:----|----:----|----:----|----:----|----:----|----:----|----:----|----:----|----:----|----:----|----:----|

T C R D V A S T A S L G S V F Q C L T T I V V M N F F L M S N L N L P S F S L K S F L T A L P D K E F1

L A G M * H P Q L L W A A C S S A S P P L * * * I S S * C L I * I Y L L L V * S H F S L H S L I K S F2

L Q G C S I H S F S G Q R V P V P H H H C S D E F L P D V * F K S T F F * F E V I S H C T P * * R V F3

13051 ACTTGCAGGGATGTAGCATCCACAGCTTCTCTGGGCAGCGTGTTCCAGTGCCTCACCACCATTGTAGTGATGAATTTCTTCCTGATGTCTAATTTAAATCTACCTTCTTTTAGTTTGAAGTCATTTCTCACTGCACTCCCTGATAAAGAG 13200

----:----|----:----|----:----|----:----|----:----|----:----|----:----|----:----|----:----|----:----|----:----|----:----|----:----|----:----|----:----|

S L P S F I E E T L C S Y * T T A E L F P S I V L Y L Y V * E L F C A D I V * M N A S S S F K F D T F1

P S P A L L K R P S V A T E L L L N C F L A L Y Y T C M F K N F S V L I * F K * M Q V V A L N L I Q F2

P P Q L Y * R D P L * L L N Y C * T V S * H C I I P V C L R T F L C * Y S L N E C K * * L * I * Y K F3

13201 TCCCTCCCCAGCTTTATTGAAGAGACCCTCTGTAGCTACTGAACTACTGCTGAACTGTTTCCTAGCATTGTATTATACCTGTATGTTTAAGAACTTTTCTGTGCTGATATAGTTTAAATGAATGCAAGTAGTAGCTTTAAATTTGATACA 13350

----:----|----:----|----:----|----:----|----:----|----:----|----:----|----:----|----:----|----:----|----:----|----:----|----:----|----:----|----:----|

S R R N V S Q C Y * G * * N M H * S T * P G C K P N S F C R V P W A K A R Q A Y F S R G K N K Q S L F1

V G G M S H S V I K D D K I C T N P R N L A V N Q I L S A G Y H G L R L D R L I L A V E R T S S P W F2

* E E C L T V L L R M I K Y A L I H V T W L * T K F F L Q G T M G * G S T G L F * P W K E Q A V L G F3

13351 AGTAGGAGGAATGTCTCACAGTGTTATTAAGGATGATAAAATATGCACTAATCCACGTAACCTGGCTGTAAACCAAATTCTTTCTGCAGGGTACCATGGGCTAAGGCTCGACAGGCTTATTTTAGCCGTGGAAAGAACAAGCAGTCCTTG 13500

----:----|----:----|----:----|----:----|----:----|----:----|----:----|----:----|----:----|----:----|----:----|----:----|----:----|----:----|----:----|

D A V E K A A F F V T L D D D E Q G Y S K E D P V S S L D A Y A K S L I H G R C Y D R Y S I D S V K F1

M L L K K Q H F L * H W M M M N K G T A K K I Q * A H * M H M Q N P * Y M A D V M T G T V L T L * K F2

C C * K S S I F C D I G * * * T R V Q Q R R S S E L T R C I C K I L N T W Q M L * Q V Q Y * L C E K F3

13501 GATGCTGTTGAAAAAGCAGCATTTTTTGTGACATTGGATGATGATGAACAAGGGTACAGCAAAGAAGATCCAGTGAGCTCACTAGATGCATATGCAAAATCCTTAATACATGGCAGATGTTATGACAGGTACAGTATTGACTCTGTGAAA 13650

----:----|----:----|----:----|----:----|----:----|----:----|----:----|----:----|----:----|----:----|----:----|----:----|----:----|----:----|----:----|

R P F S F S K T C * F M W L M H M S * * L I S I * I C P F Q T K K K T I V F E S F * Q T * S * S I K F1

D P S L F Q R L A N L C G * C I * V S D L * A Y K F V H F K Q K K K P L C L N L F D R H E V R V * K F2

T L L F F K D L L I Y V A N A Y E L V T Y K H I N L S I S N K K K N H C V * I F L T D M K L E Y K N F3

13651 AGACCCTTCTCTTTTTCAAAGACTTGCTAATTTATGTGGCTAATGCATATGAGTTAGTGACTTATAAGCATATAAATTTGTCCATTTCAAACAAAAAAAAAAACCATTGTGTTTGAATCTTTTTGACAGACATGAAGTTAGAGTATAAAA 13800

----:----|----:----|----:----|----:----|----:----|----:----|----:----|----:----|----:----|----:----|----:----|----:----|----:----|----:----|----:----|

M F L K Y K N S * S K S N L F V G G L I K R L L L * Y S K M A K W A * M L S T L G Q M L L L L D T C F1

C F * N T K I H K A N L I F L * V V * * N V Y S C S I Q K W Q N G P E C * A L L G R C S Y C W T P V F2

V S E I Q K F I K Q I * S F C R W F D K T F T L V V F K N G K M G L N A E H S W A D A P I V G H L W F3

13801 ATGTTTCTGAAATACAAAAATTCATAAAGCAAATCTAATCTTTTTGTAGGTGGTTTGATAAAACGTTTACTCTTGTAGTATTCAAAAATGGCAAAATGGGCCTGAATGCTGAGCACTCTTGGGCAGATGCTCCTATTGTTGGACACCTGT 13950

----:----|----:----|----:----|----:----|----:----|----:----|----:----|----:----|----:----|----:----|----:----|----:----|----:----|----:----|----:----|

G R * V F H T K G * C R I C * M L H A T A L N N I R F L S S L L Q L C C * * V I L D P F C L L C C Q F1

G G K C F I Q K V N V E Y V R C F M Q Q L * I T S G S F P V S C N Y V A D E * F W I P F V F S V V S F2

E V S V S Y K R L M * N M L D A S C N S S E * H Q V P F Q S L A T M L L M S D F G S L L S S L L S A F3

13951 GGGAGGTAAGTGTTTCATACAAAAGGTTAATGTAGAATATGTTAGATGCTTCATGCAACAGCTCTGAATAACATCAGGTTCCTTTCCAGTCTCTTGCAACTATGTTGCTGATGAGTGATTTTGGATCCCTTTTGTCTTCTCTGTTGTCAG 14100

----:----|----:----|----:----|----:----|----:----|----:----|----:----|----:----|----:----|----:----|----:----|----:----|----:----|----:----|----:----|

Q N * K Q T S K T C * K Q I A L P * I * G L Y * N L M T T D V F I E K * Y I T L S S L F L I F * I K F1

R I K N K Q A K L A K N K L H C L K F K V F I K I L * Q Q M C L * K S D I S P Y L A C F * Y S K S N F2

E L K T N K Q N L L K T N C T A L N L R S L L K S Y D N R C V Y R K V I Y H P I * L V F N I L N Q T F3

14101 CAGAATTAAAAACAAACAAGCAAAACTTGCTAAAAACAAATTGCACTGCCTTAAATTTAAGGTCTTTATTAAAATCTTATGACAACAGATGTGTTTATAGAAAAGTGATATATCACCCTATCTAGCTTGTTTTTAATATTCTAAATCAAA 14250

----:----|----:----|----:----|----:----|----:----|----:----|----:----|----:----|----:----|----:----|----:----|----:----|----:----|----:----|----:----|

L L P V * H C A S L L V Y T C S * S T * A S L C C K I P H L F L K L I N A T E Y N F F P * S Y F * V F1

C C L F D I A L H C L Y I R A P E A L K L P C A V K S H T Y F * N * * M L R S I T S F P E A I F E F F2

V A C L T L R F I A C I Y V L L K H L S F L V L * N P T L I F K T D K C Y G V * L L S L K L F L S S F3

14251 CTGTTGCCTGTTTGACATTGCGCTTCATTGCTTGTATATACGTGCTCCTGAAGCACTTAAGCTTCCTTGTGCTGTAAAATCCCACACTTATTTTTAAAACTGATAAATGCTACGGAGTATAACTTCTTTCCCTGAAGCTATTTTTGAGTT 14400

----:----|----:----|----:----|----:----|----:----|----:----|----:----|----:----|----:----|----:----|----:----|----:----|----:----|----:----|----:----|

L D * * I I * I C S C V L L Q * M V * M H L F S D L F I M K I V Y C L K S H L N I F S L * S L D L * F1

* I D R L F K F A L V F C C S E W Y K C T C S Q I C S S * K * F I A * N P I * I F F L F E V * T Y K F2

R L I D Y L N L L L C F A A V N G I N A L V L R F V H H E N S L L L E I P S E Y F F S L K F R P I N F3

14401 CTAGATTGATAGATTATTTAAATTTGCTCTTGTGTTTTGCTGCAGTGAATGGTATAAATGCACTTGTTCTCAGATTTGTTCATCATGAAAATAGTTTATTGCTTGAAATCCCATCTGAATATTTTTTCTCTTTGAAGTTTAGACCTATAA 14550

----:----|----:----|----:----|----:----|----:----|----:----|----:----|----:----|----:----|----:----|----:----|----:----|----:----|----:----|----:----|

T V M T S S L N I I S E T L A N S D L I M Y L C K W N P G P T D A P S H V L I L L S M S T H L H K K F1

Q L * L H H S I L F Q K H * L I L I * L C I C A S G T Q V Q L M P Q V M S * F Y Y L * V P I C I R R F2

S Y D F I T Q Y Y F R N T S * F * F N Y V S V Q V E P R S N * C P K S C P D F I I Y E Y P F A * E E F3

14551 ACAGTTATGACTTCATCACTCAATATTATTTCAGAAACACTAGCTAATTCTGATTTAATTATGTATCTGTGCAAGTGGAACCCAGGTCCAACTGATGCCCCAAGTCATGTCCTGATTTTATTATCTATGAGTACCCATTTGCATAAGAAG 14700

----:----|----:----|----:----|----:----|----:----|----:----|----:----|----:----|----:----|----:----|----:----|----:----|----:----|----:----|----:----|

R K S * L Y S L N G R K S K L I R C * L S S L V Q T S C S R Q W K R I T L L Y F R E S L S M L S S Y F1

E S P N Y I L * M G E S Q N S L D V D * A V * F R L A V L G S G K E L L C C I S E N H S V C L V H I F2

K V L T I F S K W E K V K T H * M L I E Q S S S D * L F S A V E K N Y F V V F Q R I T Q Y A * F I S F3

14701 AGAAAGTCCTAACTATATTCTCTAAATGGGAGAAAGTCAAAACTCATTAGATGTTGATTGAGCAGTCTAGTTCAGACTAGCTGTTCTCGGCAGTGGAAAAGAATTACTTTGTTGTATTTCAGAGAATCACTCAGTATGCTTAGTTCATAT 14850

----:----|----:----|----:----|----:----|----:----|----:----|----:----|----:----|----:----|----:----|----:----|----:----|----:----|----:----|----:----|

L C T Y C * S V P F L L R I G K P K V K E N S V S * E I L A V S * L V C C V H * L R K L K R L E I S F1

F V L T V S Q S L F C * E L G S P K * R R I Q F P K K F W Q F H S W C A V S T N * E S * R D * K * V F2

L Y L L L V S P F S A K N W E A Q S K G E F S F L R N S G S F I V G V L C P L T E K V E E I R N K Y F3

14851 CTTTGTACTTACTGTTAGTCAGTCCCTTTTCTGCTAAGAATTGGGAAGCCCAAAGTAAAGGAGAATTCAGTTTCCTAAGAAATTCTGGCAGTTTCATAGTTGGTGTGCTGTGTCCACTAACTGAGAAAGTTGAAGAGATTAGAAATAAGT 15000

----:----|----:----|----:----|----:----|----:----|----:----|----:----|----:----|----:----|----:----|----:----|----:----|----:----|----:----|----:----|

M Y M * Q I C I K L L S T L N R L I I S R L K T K Q K H Q K T A Q S F T * N L V R R S L C C S V C I F1

C T C N R F V S N F F P H * T D * * S Q G * K Q N K N T K K Q H N L S L E I L * G G V F V V Q F A L F2

V H V T D L Y Q T S F H I E Q I D N L K V K N K T K T P K N S T I F H L K S C K A E S L L F S L H C F3

15001 ATGTACATGTAACAGATTTGTATCAAACTTCTTTCCACATTGAACAGATTGATAATCTCAAGGTTAAAAACAAAACAAAAACACCAAAAAACAGCACAATCTTTCACTTGAAATCTTGTAAGGCGGAGTCTTTGTTGTTCAGTTTGCATT 15150

----:----|----:----|----:----|----:----|----:----|----:----|----:----|----:----|----:----|----:----|----:----|----:----|----:----|----:----|----:----|

V R L E N * P K K S N V C I K C T E T L S S M Q I L K R I Q Y F * L I F W L E F A L N L Q * T V F C F1

S V L K T S P R N L M C V * N V L K H * A A C R S * K E S N I F D * F F G * N L H L I F N K L F F V F2

P S * K L A Q E I * C V Y K M Y * N I E Q H A D L E K N P I F L I N F L V R I C T * S S I N C F L L F3

15151 GTCCGTCTTGAAAACTAGCCCAAGAAATCTAATGTGTGTATAAAATGTACTGAAACATTGAGCAGCATGCAGATCTTGAAAAGAATCCAATATTTTTGATTAATTTTTTGGTTAGAATTTGCACTTAATCTTCAATAAACTGTTTTTTGT 15300

----:----|----:----|----:----|----:----|----:----|----:----|----:----|----:----|----:----|----:----|----:----|----:----|----:----|----:----|----:----|

* T G F I R Y Q T Y R N C L E S K L D K G * K P F L G E V * L K C F A P L W W R I L C L S G I F L L F1

E L V S S D I R L T E I V * N Q N W T K V E S L F W G R F N * S V L H H C G G E F Y V Y L V F S C L F2

N W F H Q I S D L Q K L S R I K I G Q R L K A F S G G G L I K V F C T T V V E N F M S I W Y F L A * F3

15301 TGAACTGGTTTCATCAGATATCAGACTTACAGAAATTGTCTAGAATCAAAATTGGACAAAGGTTGAAAGCCTTTTCTGGGGGAGGTTTAATTAAAGTGTTTTGCACCACTGTGGTGGAGAATTTTATGTCTATCTGGTATTTTCTTGCTT 15450

----:----|----:----|----:----|----:----|----:----|----:----|----:----|----:----|----:----|----:----|----:----|----:----|----:----|----:----|----:----|

R S K D A L K * Q A I F E N R L R F A V C L K M C Y P K C V G M C V Q N C I L L * N * N L M C L L K F1

E V K M P * N N R Q Y L R I D C V L L C V * K C V I L N V L E C V F K I A Y C C E I R I * C V C * K F2

K * R C L K I T G N I * E * T A F C C V F K N V L S * M C W N V C S K L H I V V K L E F N V F A E N F3

15451 AGAAGTAAAGATGCCTTAAAATAACAGGCAATATTTGAGAATAGACTGCGTTTTGCTGTGTGTTTAAAAATGTGTTATCCTAAATGTGTTGGAATGTGTGTTCAAAATTGCATATTGTTGTGAAATTAGAATTTAATGTGTTTGCTGAAA 15600

----:----|----:----|----:----|----:----|----:----|----:----|----:----|----:----|----:----|----:----|----:----|----:----|----:----|----:----|----:----|

T * F * K R I F L H R M * W Q L S I L N W A T W K T D T A K E I P I K I F L S L P N Y S G K F Q K R F1

R N S E N V S F F T E C D G N * V S * T G L L G R R T L Q R R Y Q S K Y S Y P Y Q T T V G N S R R G F2

V I L K T Y L S S Q N V M A T E Y L E L G Y L E D G H C K G D T N Q N I P I P T K L Q W E I P E E V F3

15601 ACGTAATTCTGAAAACGTATCTTTCTTCACAGAATGTGATGGCAACTGAGTATCTTGAACTGGGCTACTTGGAAGACGGACACTGCAAAGGAGATACCAATCAAAATATTCCTATCCCTACCAAACTACAGTGGGAAATTCCAGAAGAGG 15750

----:----|----:----|----:----|----:----|----:----|----:----|----:----|----:----|----:----|----:----|----:----|----:----|----:----|----:----|----:----|

* E H R R W * L G K G A Q L F L S G Q I T * L I T E N L I S S * L S * A L * N V H F A C A P C P V Y F1

K S T E D G D W G K E L S F S * A V K S H N * L L K T L * V V N F L E P C K M C I L L V R P A Q F T F2

R A Q K M V T G E R S S A F L E R S N H I I N Y * K P Y K * L T F L S P V K C A F C L C A L P S L L F3

15751 TAAGAGCACAGAAGATGGTGACTGGGGAAAGGAGCTCAGCTTTTCTTGAGCGGTCAAATCACATAATTAATTACTGAAAACCTTATAAGTAGTTAACTTTCTTGAGCCCTGTAAAATGTGCATTTTGCTTGTGCGCCCTGCCCAGTTTAC 15900

----:----|----:----|----:----|----:----|----:----|----:----|----:----|----:----|----:----|----:----|----:----|----:----|----:----|----:----|----:----|

L K G W R G F N T A F * K F C S G N S S F L V D I F L A * Q L V K N E W P S I V N R N S T T V K F K F1

* K G G E V L T L L F K N S V Q E I A A F * * T F S * L S N L S K M N G H P L * T G T V L Q * S L S F2

K R V E R F * H C F L K I L F R K * Q L F S R H F L S L A T C Q K * M A I H C K Q E Q Y Y S E V * V F3

15901 TTAAAAGGGTGGAGAGGTTTTAACACTGCTTTTTAAAAATTCTGTTCAGGAAATAGCAGCTTTTTAGTAGACATTTTCTTAGCTTAGCAACTTGTCAAAAATGAATGGCCATCCATTGTAAACAGGAACAGTACTACAGTGAAGTTTAAG 16050

----:----|----:----|----:----|----:----|----:----|----:----|----:----|----:----|----:----|----:----|----:----|----:----|----:----|----:----|----:----|

L L L C S Y Y A C V T V P * D Y L S Y M D S E F C L D V N N V I C F F P R V Q D K L P * S Y F I G W F1

C Y C V L I M L V * Q C L E I I C L I W I Q N S V * M L I M * F V S F P E F K T N Y H D P I S * A G F2

A T V F L L C L C N S A L R L S V L Y G F R I L S R C * * C N L F L S P S S R Q T T M I L F H R L V F3

16051 TTGCTACTGTGTTCTTATTATGCTTGTGTAACAGTGCCTTGAGATTATCTGTCTTATATGGATTCAGAATTCTGTCTAGATGTTAATAATGTAATTTGTTTCTTTCCCCGAGTTCAAGACAAACTACCATGATCCTATTTCATAGGCTGG 16200

----:----|----:----|----:----|----:----|----:----|----:----|----:----|----:----|----:----|----:----|----:----|----:----|----:----|----:----|----:----|

* Y A * C S S D H G Q S * Y V F L L L F * E H F W T P N V Y W L K T F * L F F L K L I G P * L S H K F1

D M L N V Q V I M D N L N M F F C C C F K S I F G L Q T C I G L K L F N Y F F * S * L D L D F P T N F2

I C L M F K * S W T I L I C F F V V V L R A F L D S K R V L A * N F L I I F F K A N W T L T F P Q I F3

16201 TGATATGCTTAATGTTCAAGTGATCATGGACAATCTTAATATGTTTTTTTGTTGTTGTTTTAAGAGCATTTTTGGACTCCAAACGTGTATTGGCTTAAAACTTTTTAATTATTTTTTTTAAAGCTAATTGGACCTTGACTTTCCCACAAA 16350

----:----|----:----|----:----|----:----|----:----|----:----|----:----|----:----|----:----|----:----|----:----|----:----|----:----|----:----|----:----|

Y T F F C S K G R T F K L * C * W L I K R E H L F C I S * T C V P F S C I S F E C F Q M L V L V Y I F1

I P S F A P K E E R L N Y D V N G L * K G N I Y F V S V K R V C P F P A F L L N A F K C L Y L Y I F F2

Y L L L L Q R K N V * T M M L M A Y K K G T F I L Y Q L N V C A L F L H F F * M L S N A C T C I Y L F3

16351 TATACCTTCTTTTGCTCCAAAGGAAGAACGTTTAAACTATGATGTTAATGGCTTATAAAAAGGGAACATTTATTTTGTATCAGTTAAACGTGTGTGCCCTTTTCCTGCATTTCTTTTGAATGCTTTCAAATGCTTGTACTTGTATATATT 16500

----:----|----:----|----:----|----:----|----:----|----:----|----:----|----:----|----:----|----:----|----:----|----:----|----:----|----:----|----:----|

C I * I S N L L V Y F S A K M * S R G L * A L L E L W Q M M W T S I H S T L M F L G R G * * R K Q K F1

A Y R * V I Y L S I S V P R C D R E V S E H C * S S G R * C G L L F I L L * C F W E G A N K E S K N F2

H I D K * F T C L F Q C Q D V I E R S L S T A R A L A D D V D F Y S F Y F D V F G K G L I K K A K T F3

16501 TGCATATAGATAAGTAATTTACTTGTCTATTTCAGTGCCAAGATGTGATCGAGAGGTCTCTGAGCACTGCTAGAGCTCTGGCAGATGATGTGGACTTCTATTCATTCTACTTTGATGTTTTTGGGAAGGGGCTAATAAAGAAAGCAAAAA 16650

----:----|----:----|----:----|----:----|----:----|----:----|----:----|----:----|----:----|----:----|----:----|----:----|----:----|----:----|----:----|

P A L M P S F N L P C S L L T T E * E Q I L T Y T V A N L I R F C V S V F V I R T C D C V K Y T V L F1

Q P * C L H S T C P A A C S L P S K N R F * L I L * P I * * D S V F L Y L * L E R V I V * S T L Y F F2

S P D A F I Q L A L Q L A H Y R V R T D F D L Y C S Q F N K I L C F C I C D * N V * L C K V H C T S F3

16651 CCAGCCCTGATGCCTTCATTCAACTTGCCCTGCAGCTTGCTCACTACCGAGTAAGAACAGATTTTGACTTATACTGTAGCCAATTTAATAAGATTCTGTGTTTCTGTATTTGTGATTAGAACGTGTGATTGTGTAAAGTACACTGTACTT 16800

----:----|----:----|----:----|----:----|----:----|----:----|----:----|----:----|----:----|----:----|----:----|----:----|----:----|----:----|----:----|

P I N F A L L V * G Y * R H L V E V S T N A K Q I T S R K F I V Q R L T Y * C * C S F F S S A * F F F1

Q L T L H C L C K V I K D I * W K S V Q M L N R S P A G N S L S K G * L I D A N A V F S L L L S F F F2

N * L C T A C V R L L K T F S G S Q Y K C * T D H Q P E I H C P K V D L L M L M Q F F L F C L V F F F3

16801 CCAATTAACTTTGCACTGCTTGTGTAAGGTTATTAAAGACATTTAGTGGAAGTCAGTACAAATGCTAAACAGATCACCAGCCGGAAATTCATTGTCCAAAGGTTGACTTATTGATGCTAATGCAGTTTTTTCTCTTCTGCTTAGTTTTTT 16950

----:----|----:----|----:----|----:----|----:----|----:----|----:----|----:----|----:----|----:----|----:----|----:----|----:----|----:----|----:----|

F Y P * V S F R Y E N F C R * I C Y C C * N L H S I K L T C * F G V P L N S V V V N I P F L K E I N F1

F I P R Y L S D M K T S V G E F V I A V K T Y I A L N L P V S L E S P S I L * * * T F H S L K R L I F2

L S L G I F Q I * K L L * V N L L L L L K P T * H * T Y L L V W S P P Q F C S S E H S I P * R D * L F3

16951 TTTTATCCCTAGGTATCTTTCAGATATGAAAACTTCTGTAGGTGAATTTGTTATTGCTGTTAAAACCTACATAGCATTAAACTTACCTGTTAGTTTGGAGTCCCCCTCAATTCTGTAGTAGTGAACATTCCATTCCTTAAAGAGATTAAT 17100

----:----|----:----|----:----|----:----|----:----|----:----|----:----|----:----|----:----|----:----|----:----|----:----|----:----|----:----|----:----|

* C A C L L V F F L V S S P S F F S L R I F I E C P H S K M L C I L A V I V * Y L L V S A L L I Y L F1

D V P A C W F F F L L A H L L F F P C V F S * N A H I A K C F A F * L S L F N I Y W Y Q L S * F I L F2

M C L P V G F F S C * L T F F F F P A Y F H R M P T * Q N A L H F S C H C L I F I G I S S P N L S C F3

17101 TGATGTGCCTGCCTGTTGGTTTTTTTTCTTGTTAGCTCACCTTCTTTTTTTTCCCTGCGTATTTTCATAGAATGCCCACATAGCAAAATGCTTTGCATTTTAGCTGTCATTGTTTAATATTTATTGGTATCAGCTCTCCTAATTTATCTT 17250

----:----|----:----|----:----|----:----|----:----|----:----|----:----|----:----|----:----|----:----|----:----|----:----|----:----|----:----|----:----|

V G F L T Q F Q M S K H T D S L A A I T T Y C L C Y H S L G W L H G K * L D F H T P S Q A P F I F I F1

L V F * R S S K C Q S I Q I P L L L S L L T V F V T I P * G G Y M A N N L I F T H H P K H L L F L Y F2

W F F N A V P N V K A Y R F P C C Y H Y L L S L L P F L R V A T W Q I T * F S H T I P S T F Y F Y I F3

17251 GTTGGTTTTTTAACGCAGTTCCAAATGTCAAAGCATACAGATTCCCTTGCTGCTATCACTACTTACTGTCTTTGTTACCATTCCTTAGGGTGGCTACATGGCAAATAACTTGATTTTCACACACCATCCCAAGCACCTTTTATTTTTATA 17400

----:----|----:----|----:----|----:----|----:----|----:----|----:----|----:----|----:----|----:----|----:----|----:----|----:----|----:----|----:----|

F S I T I * S L N L K L N K L L Q P G R V G V M Y * I S F C N I K T T E S M G N K K A A V F G F * R F1

F Q * P S N L L I * S * T N F C N L E E L V S C T R * A F A I * K L Q N L W V I R K Q L F L D F E D F2

F N N H L I S * F E A E Q T F A T W K S W C H V L D K L L Q Y K N Y R I Y G * * E S S C F W I L K I F3

17401 TTTTCAATAACCATCTAATCTCTTAATTTGAAGCTGAACAAACTTTTGCAACCTGGAAGAGTTGGTGTCATGTACTAGATAAGCTTTTGCAATATAAAAACTACAGAATCTATGGGTAATAAGAAAGCAGCTGTTTTTGGATTTTGAAGA 17550

----:----|----:----|----:----|----:----|----:----|----:----|----:----|----:----|----:----|----:----|----:----|----:----|----:----|----:----|----:----|

S F S V F T F S D H I L F S L F L S S C C H T N L C Y * A L N * K Y C R L L N Y S C V S * V A T E L F1

R F Q F S H S Q I T F F F H F S C P P A A T Q I Y A I K L * I E N I V G Y L T T A V L V K * P L N F F2

V F S F H I L R S H S F F T F L V L L L P H K S M L L S F E L K I L * V T * L Q L C * L S S H * T F F3

17551 TCGTTTTCAGTTTTCACATTCTCAGATCACATTCTTTTTTCACTTTTCTTGTCCTCCTGCTGCCACACAAATCTATGCTATTAAGCTTTGAATTGAAAATATTGTAGGTTACTTAACTACAGCTGTGTTAGTTAAGTAGCCACTGAACTT 17700

----:----|----:----|----:----|----:----|----:----|----:----|----:----|----:----|----:----|----:----|----:----|----:----|----:----|----:----|----:----|

S N K L L I V F E L S S L Y D E P K K S G Y K N V * R S L C * F G C S R S I Y Q I S L L F F S V R L F1

L T S F * * F L N * V L F M T N Q R R V G I K T C S V A F A S L V V L E A F I K S H Y Y F F L * D S F2

* Q A F D S F * I E F S L * R T K E E W V * K R V A * P L L V W L F * K H L S N L I I I F F C K T L F3

17701 TCTAACAAGCTTTTGATAGTTTTTGAATTGAGTTCTCTTTATGACGAACCAAAGAAGAGTGGGTATAAAAACGTGTAGCGTAGCCTTTGCTAGTTTGGTTGTTCTAGAAGCATTTATCAAATCTCATTATTATTTTTTTCTGTAAGACTC 17850

----:----|----:----|----:----|----:----|----:----|----:----|----:----|----:----|----:----|----:----|----:----|----:----|----:----|----:----|----:----|

C * W * T T N N L K C C L T C G V I R I L C A Y L E C Y H * L R K R G * E * L S H * V L L F Q L S R F1

A N G K L L T T * N V A * L V E * F V S S V L T L N A T I D * E R E A K S D * A I K S F F F N F Q G F2

L M V N Y * Q P K M L L N L W S D S Y P L C L P * M L P L I K K E R L R V T K P L S P S F S T F K D F3

17851 TGCTAATGGTAAACTACTAACAACCTAAAATGTTGCTTAACTTGTGGAGTGATTCGTATCCTCTGTGCTTACCTTGAATGCTACCATTGATTAAGAAAGAGAGGCTAAGAGTGACTAAGCCATTAAGTCCTTCTTTTTCAACTTTCAAGG 18000

----:----|----:----|----:----|----:----|----:----|----:----|----:----|----:----|----:----|----:----|----:----|----:----|----:----|----:----|----:----|

T W E N F L * H T R P L * H A C S E K A E L K P S G H V P L N H V I L F K P W R T Q V K V * V D C V F1

H G K I F F D I R G L Y D T P V Q R R Q N * N R P V M Y H * I M * F C S N H G E P K * K C K L T V L F2

M G K F S L T Y E A S M T R L F R E G R T E T V R S C T I E S C N F V Q T M E N P S E S V S * L C * F3

18001 ACATGGGAAAATTTTCTTTGACATACGAGGCCTCTATGACACGCCTGTTCAGAGAAGGCAGAACTGAAACCGTCCGGTCATGTACCATTGAATCATGTAATTTTGTTCAAACCATGGAGAACCCAAGTGAAAGTGTAAGTTGACTGTGTT 18150

----:----|----:----|----:----|----:----|----:----|----:----|----:----|----:----|----:----|----:----|----:----|----:----|----:----|----:----|----:----|

D F * Y N P * S T Q L A L F I R V N F C I V F N I * R K G D A S F * E R K C S S R * L S S S S I R T F1

I S N T I H K A R S * H F L F V * I S V * F L T F K E R V M P R F E N G S V A A D N C L H P L S E R F2

F L I Q S I K H A V S T F Y S C K F L Y S F * H L K K G * C L V L R T E V * Q Q I T V F I L Y Q N G F3

18151 GATTTCTAATACAATCCATAAAGCACGCAGTTAGCACTTTTTATTCGTGTAAATTTCTGTATAGTTTTTAACATTTAAAGAAAGGGTGATGCCTCGTTTTGAGAACGGAAGTGTAGCAGCAGATAACTGTCTTCATCCTCTATCAGAACG 18300

----:----|----:----|----:----|----:----|----:----|----:----|----:----|----:----|----:----|----:----|----:----|----:----|----:----|----:----|----:----|

A G I K G Y V K R E * L V T N N Q I C Q L L W I L N S R K L M I F V N F L L F L K Q R M S R Q T L N F1

Q E L K A T S K E N D * S L I T K Y A N C Y G S * T P E N * * Y L * I S C Y F * S K G C H A K L S I F2

R N * R L R Q K R M T S H * * P N M P T V M D P K L Q K T D D I C E F L V I F E A K D V T P N S Q L F3

18301 GCAGGAATTAAAGGCTACGTCAAAAGAGAATGACTAGTCACTAATAACCAAATATGCCAACTGTTATGGATCCTAAACTCCAGAAAACTGATGATATTTGTGAATTTCTTGTTATTTTTGAAGCAAAGGATGTCACGCCAAACTCTCAAT 18450

----:----|----:----|----:----|----:----|----:----|----:----|----:----|----:----|----:----|----:----|----:----|----:----|----:----|----:----|----:----|

Y H * R T L L W C E N W L S * I A A V E L I G S V * Q N V L N I Q Q A L E I S * N K I W N E R I L V F1

T T K E L Y C G V R I G S L E L L L * S * * V R F N R M C L T F S K L * R Y L R I K F G M N A F * F F2

P L K N F I V V * E L A L L N C C C R V D R F G L T E C A * H S A S S R D I L E * N L E * T H F S F F3

18451 TACCACTAAAGAACTTTATTGTGGTGTGAGAATTGGCTCTCTTGAATTGCTGCTGTAGAGTTGATAGGTTCGGTTTAACAGAATGTGCTTAACATTCAGCAAGCTCTAGAGATATCTTAGAATAAAATTTGGAATGAACGCATTTTAGTT 18600

----:----|----:----|----:----|----:----|----:----|----:----|----:----|----:----|----:----|----:----|----:----|----:----|----:----|----:----|----:----|

L T W Y L T R I L T Y H C E T V R F I E M W A L P N V H I P T P S S P * I Y C F C I F * Y I A Y S H F1

* H G I * R G F * P T I V K L F G S * K C G H F L T C I Y P H H H P P E Y T V S A F F D T * L I H I F2

N M V F N E D S D L P L * N C S V H R N V G T S * R A Y T H T I I P L N I L F L H F L I H S L F T L F3

18601 TTAACATGGTATTTAACGAGGATTCTGACCTACCATTGTGAAACTGTTCGGTTCATAGAAATGTGGGCACTTCCTAACGTGCATATACCCACACCATCATCCCCCTGAATATACTGTTTCTGCATTTTTTGATACATAGCTTATTCACAT 18750

----:----|----:----|----:----|----:----|----:----|----:----|----:----|----:----|----:----|----:----|----:----|----:----|----:----|----:----|----:----|

C L E S Y V G R Y L C * Y T Q M L I Q S F L I L A F T A C F L P V C F C I E F V S S L D Y P Q S F T F1

V W K A M W V D T F A S I L R C * F S L F * Y L P L L L A S Y L C V F A * N L C P P W T I H K A L L F2

F G K L C G * I P L L V Y S D V N S V F F N T C L Y C L L L T C V F L H R I C V L P G L S T K L Y S F3

18751 TGTTTGGAAAGCTATGTGGGTAGATACCTTTGCTAGTATACTCAGATGTTAATTCAGTCTTTTTTAATACTTGCCTTTACTGCTTGCTTCTTACCTGTGTGTTTTTGCATAGAATTTGTGTCCTCCCTGGACTATCCACAAAGCTTTACT 18900

----:----|----:----|----:----|----:----|----:----|----:----|----:----|----:----|----:----|----:----|----:----|----:----|----:----|----:----|----:----|

P E C Q * * A C L A Q L P V I Y K F S S R R L T G C S E H L E * V A * N I M M L D L S D R L P Y F Q F1

Q N A N S R H A W R N Y Q * F I S F H P G D * Q A V Q S I * N K W H K I * * C W T * V T G Y H I F S F2

R M P I V G M L G A T T S D L * V F I P E T N R L F R A S R I S G I K Y N D A G P K * Q V T I F S V F3

18901 CCAGAATGCCAATAGTAGGCATGCTTGGCGCAACTACCAGTGATTTATAAGTTTTCATCCCGGAGACTAACAGGCTGTTCAGAGCATCTAGAATAAGTGGCATAAAATATAATGATGCTGGACCTAAGTGACAGGTTACCATATTTTCAG 19050

----:----|----:----|----:----|----:----|----:----|----:----|----:----|----:----|----:----|----:----|----:----|----:----|----:----|----:----|----:----|

C C I * Y Y K I L A E L S * S E F E T E I F F Q P L H L Y S Y T V S L P S K M A L A V P A F L K S H F1

V A F D I I K S * Q N Y L K V S L R L K F S F S P C I Y T H T L S V C L L K W L L Q F L P F L N H I F2

L H L I L * N L S R T I L K * V * D * N F L S A L A F I L I H C Q F A F * N G S C S S C L S * I T L F3

19051 TGTTGCATTTGATATTATAAAATCTTAGCAGAACTATCTTAAAGTGAGTTTGAGACTGAAATTTTCTTTCAGCCCTTGCATTTATACTCATACACTGTCAGTTTGCCTTCTAAAATGGCTCTTGCAGTTCCTGCCTTTCTTAAATCACAT 19200

----:----|----:----|----:----|----:----|----:----|----:----|----:----|----:----|----:----|----:----|----:----|----:----|----:----|----:----|----:----|

* D L I F C S F L R N * M G F T Y Q I F F I F L S S L E * K * D E V I P A C S H Q T P A L I S S C H F1

E I * F S V A F * G T K W D S L T R F F S S F F H R * N E N K M K S F R L A A T K H Q H L Y R L A M F2

R F D F L * L S K E L N G I H L P D F F H L S F I V R M K I R * S H S G L Q P P N T S T Y I V L P * F3

19201 TGAGATTTGATTTTCTGTAGCTTTCTAAGGAACTAAATGGGATTCACTTACCAGATTTTTTTCATCTTTCTTTCATCGTTAGAATGAAAATAAGATGAAGTCATTCCGGCTTGCAGCCACCAAACACCAGCACTTATATCGTCTTGCCAT 19350

----:----|----:----|----:----|----:----|----:----|----:----|----:----|----:----|----:----|----:----|----:----|----:----|----:----|----:----|----:----|

D W C W H * P P S V L P L C R F Q V P F C R F S L P Q G S K * S L F I G Y S K * P F V Y S A F S G T F1

T G A G I D R H L F C L Y V V S K Y L S V D S P F L K E V S D L Y S L V T V N N R L F I Q H F L V P F2

L V L A L T A I C S A F M S F P S T F L * I L P S S R K * V I F I H W L Q * I T V C L F S I F W Y L F3

19351 GACTGGTGCTGGCATTGACCGCCATCTGTTCTGCCTTTATGTCGTTTCCAAGTACCTTTCTGTAGATTCTCCCTTCCTCAAGGAAGTAAGTGATCTTTATTCATTGGTTACAGTAAATAACCGTTTGTTTATTCAGCATTTTCTGGTACC 19500

----:----|----:----|----:----|----:----|----:----|----:----|----:----|----:----|----:----|----:----|----:----|----:----|----:----|----:----|----:----|

C L R I N G T P V F L R K E I R V * I A W L N I L Q L V F I S I Y I Y T L T N R L S V I P C * G L Q F1

A Y V L M E L Q F S S E R R * E Y R * H G L T S Y S L F S F L Y I Y T H L L I D F Q * Y R V K V C K F2

L T Y * W N S S F P Q K G D K S I D S M A * H P T A C F H F Y I Y I H T Y * * T F S N T V L R F A R F3

19501 TGCTTACGTATTAATGGAACTCCAGTTTTCCTCAGAAAGGAGATAAGAGTATAGATAGCATGGCTTAACATCCTACAGCTTGTTTTCATTTCTATATATATATACACACTTACTAATAGACTTTCAGTAATACCGTGTTAAGGTTTGCAA 19650

----:----|----:----|----:----|----:----|----:----|----:----|----:----|----:----|----:----|----:----|----:----|----:----|----:----|----:----|----:----|

E R C N Q Y Y N K I L A L N T C S P Q A A M C F L S R I L F W N C N K I T L L * V N M S * G S H I L F1

K D V I S I T I K Y W L L T H V H R R Q L C A F S Q E Y Y F G T V T K L L C C K L T * A K G A I Y C F2

K M * S V L Q * N T G S * H M F T A G S Y V L S L K N I I L E L * Q N Y F V V S * H E L R E P Y I A F3

19651 GAAAGATGTAATCAGTATTACAATAAAATACTGGCTCTTAACACATGTTCACCGCAGGCAGCTATGTGCTTTCTCTCAAGAATATTATTTTGGAACTGTAACAAAATTACTTTGTTGTAAGTTAACATGAGCTAAGGGAGCCATATATTG 19800

----:----|----:----|----:----|----:----|----:----|----:----|----:----|----:----|----:----|----:----|----:----|----:----|----:----|----:----|----:----|

Q F L F F T V Y C S A K C F M K P * N R I L K L E F M K * N V C S C F K L R I K Q P C T A S A A F Q F1

N F C F L P F T A V P N A L * N R E T V F * S W N S * N K M C V P A L S * G S N N L V Q L L Q P S K F2

I F V F Y R L L Q C Q M L Y E T V K P Y F K V G I H E I K C V F L L * V K D Q T T L Y S F C S L P R F3

19801 CAATTTTTGTTTTTTACCGTTTACTGCAGTGCCAAATGCTTTATGAAACCGTGAAACCGTATTTTAAAGTTGGAATTCATGAAATAAAATGTGTGTTCCTGCTTTAAGTTAAGGATCAAACAACCTTGTACAGCTTCTGCAGCCTTCCAA 19950

----:----|----:----|----:----|----:----|----:----|----:----|----:----|----:----|----:----|----:----|----:----|----:----|----:----|----:----|----:----|

D I C V L L G I L Q T S N Y C S I H L G F E Y H H C * S C * Q D L L C R V Y Q A I D Q F H L F * N N F1

T S V Y C W G F Y K L L I T V V F T W D L S T T I A D L V D K T C Y A E F T R L * T S F I C F E I I F2

H L C I V G D F T N F * L L * Y S L G I * V P P L L I L L T R P A M Q S L P G Y R P V S S V L K * Y F3

19951 GACATCTGTGTATTGTTGGGGATTTTACAAACTTCTAATTACTGTAGTATTCACTTGGGATTTGAGTACCACCATTGCTGATCTTGTTGACAAGACCTGCTATGCAGAGTTTACCAGGCTATAGACCAGTTTCATCTGTTTTGAAATAAT 20100

----:----|----:----|----:----|----:----|----:----|----:----|----:----|----:----|----:----|----:----|----:----|----:----|----:----|----:----|----:----|

I S T L R N F I T S L E I N M K I * Y V D G L Y V R T S * I V S S S T L N Y S F H * H V N * S Y L L F1

F L L * E I L L L L * K L T * R S N M L M V Y T L G Q V K L F L L A L S I I H S I D M * T R V T F * F2

F Y F K K F Y Y F F R N * H E D L I C * W F I R * D K L N C F F * H S Q L F I P L T C E L E L P F N F3

20101 ATTTCTACTTTAAGAAATTTTATTACTTCTTTAGAAATTAACATGAAGATCTAATATGTTGATGGTTTATACGTTAGGACAAGTTAAATTGTTTCTTCTAGCACTCTCAATTATTCATTCCATTGACATGTGAACTAGAGTTACCTTTTA 20250

----:----|----:----|----:----|----:----|----:----|----:----|----:----|----:----|----:----|----:----|----:----|----:----|----:----|----:----|----:----|

I L L Y * I F S K L F L L F L E R V A C S * L I C C T * L Y L F L P L I K V L S E P W R L S T S Q T F1

Y C F I E Y F L S Y F Y Y F L R E L H V P N * Y V V L D Y I F F Y L * * R F C L S L G D Y Q R V R H F2

I A L L N I F * V I F I I S * E S C M F L I N M L Y L I I S F F T S N K G F V * A L E T I N E S D T F3

20251 ATATTGCTTTATTGAATATTTTCTAAGTTATTTTTATTATTTCTTGAGAGAGTTGCATGTTCCTAATTAATATGTTGTACTTGATTATATCTTTTTTTACCTCTAATAAAGGTTTTGTCTGAGCCTTGGAGACTATCAACGAGTCAGACA 20400

----:----|----:----|----:----|----:----|----:----|----:----|----:----|----:----|----:----|----:----|----:----|----:----|----:----|----:----|----:----|

P Q Q H I D L K K N P E M L S S G G G F G P V C L L N T S I P F K N S F V S D P * Y L N V T Y * K C F1

H S N T L I * R R T L R C Y L L V V D L D L Y V Y * I Q A S L L K T V L F Q T H N T L M L H I R S A F2

T A T H * S E E E P * D V I F W W W I W T C M F T K Y K H P F * K Q F C F R P I I P * C Y I L E V Q F3

20401 CCACAGCAACACATTGATCTGAAGAAGAACCCTGAGATGTTATCTTCTGGTGGTGGATTTGGACCTGTATGTTTACTAAATACAAGCATCCCTTTTAAAAACAGTTTTGTTTCAGACCCATAATACCTTAATGTTACATATTAGAAGTGC 20550

----:----|----:----|----:----|----:----|----:----|----:----|----:----|----:----|----:----|----:----|----:----|----:----|----:----|----:----|----:----|

N V R F K Y V L N I F R L Y E C A Q K N Q R H L F P L S H * N M D C S * G N M Q L S N V A * G S T A F1

M Y A L N T C L I F S D Y T N V L R K T N V T F F P C P T K I W I V L K E I C S Y Q M W R K A P Q Q F2

C T L * I R A * Y F P T I R M C S E K P T S P F S L V P L K Y G L F L R K Y A V I K C G V R L H S K F3

20551 AATGTACGCTTTAAATACGTGCTTAATATTTTCCGACTATACGAATGTGCTCAGAAAAACCAACGTCACCTTTTTCCCTTGTCCCACTAAAATATGGATTGTTCTTAAGGAAATATGCAGTTATCAAATGTGGCGTAAGGCTCCACAGCA 20700

----:----|----:----|----:----|----:----|----:----|----:----|----:----|----:----|----:----|----:----|----:----|----:----|----:----|----:----|----:----|

R R S N K L G A S I A V P M P A D Y V C Q T I I T V T I S L L L T L D F K V G I S R K * L F H C S V F1

G D L I N W G L L S L Y L C L Q I M Y V K L L L Q * Q S V Y Y L H * T L R L G S V E S N Y F T V P C F2

E I * * T G G F Y R C T Y A C R L C M S N Y Y Y S D N Q F T T Y T R L * G W D Q * K V I I S L F R V F3

20701 AGGAGATCTAATAAACTGGGGGCTTCTATCGCTGTACCTATGCCTGCAGATTATGTATGTCAAACTATTATTACAGTGACAATCAGTTTACTACTTACACTAGACTTTAAGGTTGGGATCAGTAGAAAGTAATTATTTCACTGTTCCGTG 20850

----:----|----:----|----:----|----:----|----:----|----:----|----:----|----:----|----:----|----:----|----:----|----:----|----:----|----:----|----:----|

F F I Y N S Q L T K E I I I I V H A F L * I K L L R L V C L P S * I * V V A R I R M K V F C S F C G F1

F L F I I P N * L K K * * * L F M L S F E L S Y S D W S A C L P K Y E * * L G * E * K F S A A F V D F2

F Y L * F P I N * R N N N N C S C F P L N * A T Q I G L L A F L N M S S S * D K N E S F L Q L L W T F3

20851 TTTTTTATTTATAATTCCCAATTAACTAAAGAAATAATAATAATTGTTCATGCTTTCCTTTGAATTAAGCTACTCAGATTGGTCTGCTTGCCTTCCTAAATATGAGTAGTAGCTAGGATAAGAATGAAAGTTTTCTGCAGCTTTTGTGGA 21000

----:----|----:----|----:----|----:----|----:----|----:----|----:----|----:----|----:----|----:----|----:----|----:----|----:----|----:----|----:----|

L * R S * C I G G V L M S F * L T I V F Y * D F N T E I D N I P V N S V R Q A F P C G R L D S S I S F1

F R G A S V L V E F * * V S D L L * Y F T E I L I R K L I I F L L T L L G R L F H V V D * I H L F Q F2

L E E L V Y W W S F N E F L T Y Y S I L L R F * Y G N * * Y S C * L C * A G F S M W * T R F I Y F K F3

21001 CTTTAGAGGAGCTAGTGTATTGGTGGAGTTTTAATGAGTTTCTGACTTACTATAGTATTTTACTGAGATTTTAATACGGAAATTGATAATATTCCTGTTAACTCTGTTAGGCAGGCTTTTCCATGTGGTAGACTAGATTCATCTATTTCA 21150

----:----|----:----|----:----|----:----|----:----|----:----|----:----|----:----|----:----|----:----|----:----|----:----|----:----|----:----|----:----|

K S I S I K R T V R D H R T T L W L R N H N N Y C V F * L T R Q A E S W F C G H W L I V * K V K K K F1

K A F L L K E Q * G I T G L L F G S E I T I I I V Y F S * L V K L K A G F V A I G * L C R K * R K S F2

K H F Y * K N S E G S Q D Y S L A Q K S Q * L L C I L V D S S S * K L V L W P L V D C V E S E E K V F3

21151 AAAAGCATTTCTATTAAAAGAACAGTGAGGGATCACAGGACTACTCTTTGGCTCAGAAATCACAATAATTATTGTGTATTTTAGTTGACTCGTCAAGCTGAAAGCTGGTTTTGTGGCCATTGGTTGATTGTGTAGAAAGTGAAGAAAAAG 21300

----:----|----:----|----:----|----:----|----:----|----:----|----:----|----:----|----:----|----:----|----:----|----:----|----:----|----:----|----:----|

* V * N L F L S F F F P * V A D D G Y G V S Y I I L D E N S I H F H V S S K F S C S E T V R D N P F F1

K F K T C S C H F S S L R W L M M V T V F L T * S W M R T P S I S M S P A N S L V L K R * E T I L S F2

S L K L V L V I F L P L G G * * W L R C F L H N L G * E L H P F P C L Q Q I L L F * N G K R Q S F P F3

21301 TAAGTTTAAAACTTGTTCTTGTCATTTTTCTTCCCTTAGGTGGCTGATGATGGTTACGGTGTTTCTTACATAATCTTGGATGAGAACTCCATCCATTTCCATGTCTCCAGCAAATTCTCTTGTTCTGAAACGGTAAGAGACAATCCTTTC 21450

----:----|----:----|----:----|----:----|----:----|----:----|----:----|----:----|----:----|----:----|----:----|----:----|----:----|----:----|----:----|

L F * Y L L L R L F Y S L S T K N L V G L A T Y V I F C F L L C Q H M L Y I N V F L S F Y K F S D F F1

C S D I C F * D S S T H C L L K I * * D L Q L T L Y S A F Y F V N I C C I L M F S S A S I S L V T S F2

V L I S A F K T L L L T V Y * K F S R T C N L R Y I L L F T L S T Y A V Y * C F P Q L L * V * * L H F3

21451 CTGTTCTGATATCTGCTTTTAAGACTCTTCTACTCACTGTCTACTAAAAATTTAGTAGGACTTGCAACTTACGTTATATTCTGCTTTTTACTTTGTCAACATATGCTGTATATTAATGTTTTCCTCAGCTTCTATAAGTTTAGTGACTTC 21600

----:----|----:----|----:----|----:----|----:----|----:----|----:----|----:----|----:----|----:----|----:----|----:----|----:----|----:----|----:----|

M N Q G N G V I L H L P V R C N K K K K P K V M L V H M * F S G T Y C H K Q * D R C L L V F V I T V F1

* T K E M V * Y F T F L S D V I K K K S L R S C L C T C S S L E L T A I S S K I G V S W F L L L Q F F2

E P R K W C N T S P S C Q M * * K K K A * G H A C A H V V L W N L L P * A V R * V S P G F C Y Y S L F3

21601 ATGAACCAAGGAAATGGTGTAATACTTCACCTTCCTGTCAGATGTAATAAAAAAAAAAAGCCTAAGGTCATGCTTGTGCACATGTAGTTCTCTGGAACTTACTGCCATAAGCAGTAAGATAGGTGTCTCCTGGTTTTTGTTATTACAGTT 21750

----:----|----:----|----:----|----:----|----:----|----:----|----:----|----:----|----:----|----:----|----:----|----:----|----:----|----:----|----:----|

* L * L A S G C S G K D N T V V C T L F F E S P W S K * F * W K L M D K F * S V V G K L N Y H N F S F1

S F D L P L A A V V K I T Q * Y A L Y S L N L L G P N S S D G S * W I N F N L W L G N L I I I I S R F2

A L T C L W L Q W * R * H S S M H F I L * I S L V Q I V L M E V D G * I L I C G W E T * L S * F L G F3

21751 TAGCTTTGACTTGCCTCTGGCTGCAGTGGTAAAGATAACACAGTAGTATGCACTTTATTCTTTGAATCTCCTTGGTCCAAATAGTTCTGATGGAAGTTGATGGATAAATTTTAATCTGTGGTTGGGAAACTTAATTATCATAATTTCTCG 21900

----:----|----:----|----:----|----:----|----:----|----:----|----:----|----:----|----:----|----:----|----:----|----:----|----:----|----:----|----:----|

E * H P A I E M * * C H P S I S F C C R E Q R * V S F S L S F P F V L * L T N I L S F G R I T * T L F1

N S T R L L K C S D V I H Q Y P S V A E N S V E * A S V C P S P L F Y D L P T Y S A L A G L H R P * F2

I A P G Y * N V V M S S I N I L L L Q R T A L S E L Q F V L P L C F M T Y Q H T Q L W Q D Y I D L N F3

21901 GAATAGCACCCGGCTATTGAAATGTAGTGATGTCATCCATCAATATCCTTCTGTTGCAGAGAACAGCGTTGAGTGAGCTTCAGTTTGTCCTTCCCCTTTGTTTTATGACTTACCAACATACTCAGCTTTGGCAGGATTACATAGACCTTA 22050

----:----|----:----|----:----|----:----|----:----|----:----|----:----|----:----|----:----|----:----|----:----|----:----|----:----|----:----|----:----|

T C L M D L E A S G * S L N I R K Y D F V H V W F T L F F R C L L L Y T L N F P V V L S V K A K V H F1

L V * W I L K P L V N H * I L E N T T L F M C G S H C S S D A F Y F I R * I S L * S Y Q S R Q K S I F2

L S D G S * S L W L I I K Y * K I R L C S C V V H I V L Q M P F T L Y V K F P C S L I S Q G K S P * F3

22051 ACTTGTCTGATGGATCTTGAAGCCTCTGGTTAATCATTAAATATTAGAAAATACGACTTTGTTCATGTGTGGTTCACATTGTTCTTCAGATGCCTTTTACTTTATACGTTAAATTTCCCTGTAGTCTTATCAGTCAAGGCAAAAGTCCAT 22200

----:----|----:----|----:----|----:----|----:----|----:----|----:----|----:----|----:----|----:----|----:----|----:----|----:----|----:----|----:----|

R E K A A L C V P V L D I I R V S C S R F * H V A N D L Y L * S F S S L Y H S S L G I V S * C L S N F1

E R R Q H F V C L S L I L L E F P V V G F N M * L M I Y I C N L F L P C T I H L L E * F L S V * V I F2

R E G S T L C A C P * Y Y * S F L * * V L T C S * * F I F V I F F F P V P F I S W N S F L V F K * L F3

22201 AGAGAGAAGGCAGCACTTTGTGTGCCTGTCCTTGATATTATTAGAGTTTCCTGTAGTAGGTTTTAACATGTAGCTAATGATTTATATTTGTAATCTTTTTCTTCCCTGTACCATTCATCTCTTGGAATAGTTTCTTAGTGTTTAAGTAAT 22350

----:----|----:----|----:----|----:----|----:----|----:----|----:----|----:----|----:----|----:----|----:----|----:----|----:----|----:----|----:----|

C D F H F P Y L M L F F * * I Q D S N R S S N A I F F F F F F A F S V F L G F F R C F T L S G * S F F1

V I F T F L I * C C S S S R F R I P I G A Q M Q F F F F F F L L F Q F F * G F L G V L H F L G S L L F2

* F S L S L F D V V L L V D S G F Q * E L K C N F F F F F F C F F S F F R V F * V F Y T F W V V F C F3

22351 TGTGATTTTCACTTTCCTTATTTGATGTTGTTCTTCTAGTAGATTCAGGATTCCAATAGGAGCTCAAATGCAATTTTTTTTTTTTTTTTTTTTGCTTTTTCAGTTTTTTTAGGGTTTTTTAGGTGTTTTACACTTTCTGGGTAGTCTTTT 22500

----:----|----:----|----:----|----:----|----:----|----:----|----:----|----:----|----:----|----:----|----:----|----:----|----:----|----:----|----:----|

V L * E M A * I K S S C M H * M F * F S F F K Q * Y K A I D T F D F K G R F S V L C V N N I T A * C F1

S C E K W L R * N H L V C T R C F S F L F L N N D T K L * T H L I S K V D S Q F C V * I T * L H N V F2

L V R N G L D K I I L Y A L D V L V F F F * T M I Q S Y R H I * F Q R * I L S S V C K * H N C I M * F3

22501 GTCTTGTGAGAAATGGCTTAGATAAAATCATCTTGTATGCACTAGATGTTTTAGTTTTCTTTTTTTAAACAATGATACAAAGCTATAGACACATTTGATTTCAAAGGTAGATTCTCAGTTCTGTGTGTAAATAACATAACTGCATAATGT 22650

----:----|----:----|----:----|----:----|----:----|----:----|----:----|----:----|----:----|----:----|----:----|----:----|----:----|----:----|----:----|

R I Y Q S V I A * T W I N V Y S I R T F V L I P C F M V I Y V I * I C I P K L Q N V K N S F F Y L K F1

E F T N Q * * H K P G L M Y I A L E L L F * F P V L W * Y M * S K S V F Q S Y K M L K I L S F T * K F2

N L P I S N S I N L D * C I * H * N F C S N S L F Y G N I C D L N L Y S K V T K C * K F F L L L K R F3

22651 AGAATTTACCAATCAGTAATAGCATAAACCTGGATTAATGTATATAGCATTAGAACTTTTGTTCTAATTCCCTGTTTTATGGTAATATATGTGATCTAAATCTGTATTCCAAAGTTACAAAATGTTAAAAATTCTTTCTTTTACTTAAAA 22800

----:----|----:----|----:----|----:----|----:----|----:----|----:----|----:----|----:----|----:----|----:----|----:----|----:----|----:----|----:----|

N C Y Q T A Q L I V F L F I Y * G F S S L W K E H P K S I G * H H G F V S T Y * K L Y Q M I W V I F1

I T V T R Q H S S L Y F Y L F I K D S H R F G K N I Q K A L V D I M G L F Q P T K N C T K * S G L L F2

* L L P D S T A H C I F I Y L L R I L I A L E R T S K K H W L T S W V C F N L L K T V P N D L G Y C F3

22801 GATAACTGTTACCAGACAGCACAGCTCATTGTATTTTTATTTATTTATTAAGGATTCTCATCGCTTTGGAAAGAACATCCAAAAAGCATTGGTTGACATCATGGGTTTGTTTCAACCTACTAAAAACTGTACCAAA**TGA**TCTGGGTTATT 22950

----:----|----:----|----:----|----:----|----:----|----:----|----:----|----:----|----:----|----:----|----:----|----:----|----:----|----:-**---**|----:----|

A T K Q P P I V G M E T L L N S E * K Q G L R K H * L V K K L S H N P V D L L R K P * P S L N V S * F1

P R S N L P S L G W K L F * T A N E S K V C G N T S W * R N * V T I R L I F Y E N L S Q A * M F H D F2

H E A T S H R W D G N S S E Q R M K A R F A E T L A G E E T E S Q S G * S S T K T L A K L E C F M X F3

22951 GCCACGAAGCAACCTCCCATCGTTGGGATGGAAACTCTTCTGAACAGCGAATGAAAGCAAGGTTTGCGGAAACACTAGCTGGTGAAGAAACTGAGTCACAATCCGGTTGATCTTCTACGAAAACCTTAGCCAAGCTTGAATGTTTCATGA 23100

----:----|----:----|----:----|----:----|----:----|----:----|----:----|----:----|----:----|----:----|----:----|----:----|----:----|----:----|----:----|

X F1

F2

F3

23101 T 23101

-

Several avian sequences of the ISNL5 protein are available in databases, including that of the RJF (sequence ID: XP_004936930.1). ISNL5 is composed of three exons while ISNL5 is an isoform only comprising the two outer exons. The gene encoding these proteins is available in two RJF models, within the scaffold AADN05000647.1 (positions 1,296,132 - 1,297,544) and the Gallus gallus breed Yeonsan Ogye chromosome 3 (positions 28,007,289 - 28,005,877 on minus strand). Below is supplied the sequence of the scaffold AADN05000647.1 between positions 1,295,132 - 1,298,544. Exons are highlighted in yellow. In other vertebrate lineages, the ISNL5 gene is surrounded upstream by the gene encoding the regulatory factor X1 (RFX1) and downstream by the gene encoding the interleukin 27 receptor subunit alpha (IL27RA). These two genes were not found in the AADN05000647.1 scaffold, neither in another scaffold or chromosome. In the 50,000 nucleotides located upstream the RLN3 gene we found two RJF genes encoding for mesoderm induction early response protein 1 (Sequence ID: XP_025008655.1) between positions 1,250,222 to 1,260,863 and a WD repeat-containing protein 78 for positions 1,279,633to 1,292,954. In the 50,000 nucleotides located downstream the RLN3 gene we found two RJF genes encoding for the SH3-containing GRB2-like protein 3-interacting protein 1 (Sequence ID: XP_015146594.1) between positions 1,303,754 to 1,325,982 and a cAMP-specific 3',5'-cyclic phosphodiesterase 4B from positions 1,340,835 to 1,346,889 in the scaffold AADN05000817.1.

S * L P S F P F E A F Y C G N F L L E F T A T V A N N T Q T G L G R S D S T D P L T C S P T A A P L F1

P N Y P V F H L R H F I A G T F Y * S L Q Q Q * P T T R R Q G W A A L I A L I H * P V V P R L L H S F2

L I T Q F S I * G I L L R E L F I R V Y S N S S Q Q H A D R A G P L * * H * S T D L * S H G C S T L F3

1 TCCTAATTACCCAGTTTTCCATTTGAGGCATTTTATTGCGGGAACTTTTTATTAGAGTTTACAGCAACAGTAGCCAACAACACGCAGACAGGGCTGGGCCGCTCTGATAGCACTGATCCACTGACCTGTAGTCCCACGGCTGCTCCACTC 150

----:----|----:----|----:----|----:----|----:----|----:----|----:----|----:----|----:----|----:----|----:----|----:----|----:----|----:----|----:----|

W L S D V Y Q * E K A G R M L I L S C S V L C P E P S P L F S V S F E * G P L S T D K A K Q I A M G F1

G S Q M S I S R K R L A G C * S L A V L C F A R S H R P S S P C L L S K A L C P R T K Q S K * L W V F2

A L R C L S V G K G W Q D A D P * L F C A L P G A I A P L L R V F * V R P S V H G Q S K A N S Y G C F3

151 TGGCTCTCAGATGTCTATCAGTAGGAAAAGGCTGGCAGGATGCTGATCCTTAGCTGTTCTGTGCTTTGCCCGGAGCCATCGCCCCTCTTCTCCGTGTCTTTTGAGTAAGGCCCTCTGTCCACGGACAAAGCAAAGCAAATAGCTATGGGT 300

----:----|----:----|----:----|----:----|----:----|----:----|----:----|----:----|----:----|----:----|----:----|----:----|----:----|----:----|----:----|

A R C G T T T A S L Q L A A V S S L C S K P H M L S T Q C P S A Q L Y A C T T Q G L P G P Q P A L K F1

Q G V E Q L Q H L Y S L L Q * A P C A P S P T C S A R N A L L L S S M P A Q H R G C L A L S L L * S F2

K V W N N Y S I S T A C C S E L P V L Q A P H A Q H A M P F C S A L C L H N T G A A W P S A C S E A F3

301 GCAAGGTGTGGAACAACTACAGCATCTCTACAGCTTGCTGCAGTGAGCTCCCTGTGCTCCAAGCCCCACATGCTCAGCACGCAATGCCCTTCTGCTCAGCTCTATGCCTGCACAACACAGGGGCTGCCTGGCCCTCAGCCTGCTCTGAAG 450

----:----|----:----|----:----|----:----|----:----|----:----|----:----|----:----|----:----|----:----|----:----|----:----|----:----|----:----|----:----|

H D A Q L P A A * G D A Q Q E D E A G E M E G C T T E G A H G R S Q F P S L F G I T D R A R P H I T F1

M M H S C Q Q H E G M L S R R T K Q G K W R G A Q L R V P M E E V N S H P Y L G S Q T E Q G P T S L F2

* C T A A S S M R G C S A G G R S R G N G G V H N * G C P W K K S I P I L I W D H R Q S K A P H H L F3

451 CATGATGCACAGCTGCCAGCAGCATGAGGGGATGCTCAGCAGGAGGACGAAGCAGGGGAAATGGAGGGGTGCACAACTGAGGGTGCCCATGGAAGAAGTCAATTCCCATCCTTATTTGGGATCACAGACAGAGCAAGGCCCCACATCACT 600

----:----|----:----|----:----|----:----|----:----|----:----|----:----|----:----|----:----|----:----|----:----|----:----|----:----|----:----|----:----|

C T V L T G T A G Q Q T A K E R H G D E T H Y L Q N T * * T F L Q H I H I N S T D V S P F P I N M L F1

A R C * Q A Q L G N R L Q R K G M E M K P I T Y K T H N K H F C S T Y T L T A L M * A R S L * T C Y F2

H G A N R H S W A T D C K G K A W R * N P L P T K H I I N I F A A H T H * Q H * C K P V P Y K H V M F3

601 TGCACGGTGCTAACAGGCACAGCTGGGCAACAGACTGCAAAGGAAAGGCATGGAGATGAAACCCATTACCTACAAAACACATAATAAACATTTTTGCAGCACATACACATTAACAGCACTGATGTAAGCCCGTTCCCTATAAACATGTTA 750

----:----|----:----|----:----|----:----|----:----|----:----|----:----|----:----|----:----|----:----|----:----|----:----|----:----|----:----|----:----|

C Y K Y S Y C K A V T Q S * E V F N T Q T N P Q G T * N I T T N T I G S G K H E V T L Q R A A A Q W F1

A T S I P T V K Q L H S P K K S L T P K Q T P R A L K T S P Q T Q L V L A N M R S P C S E L L P S G F2

L Q V F L L * S S Y T V L R S L * H P N K P P G H L K H H H K H N W F W Q T * G H P A A S C C P V E F3

751 TGCTACAAGTATTCCTACTGTAAAGCAGTTACACAGTCCTAAGAAGTCTTTAACACCCAAACAAACCCCCAGGGCACTTAAAACATCACCACAAACACAATTGGTTCTGGCAAACATGAGGTCACCCTGCAGCGAGCTGCTGCCCAGTGG 900

----:----|----:----|----:----|----:----|----:----|----:----|----:----|----:----|----:----|----:----|----:----|----:----|----:----|----:----|----:----|

S G W A T P I N G S F Q Q H S P P Q P H R A L L R C P P S P T A Q N E E H A A G P G C A L P A G C A F1

A D G P P L * T G A S S S T P H L S P T E H S S A A L P A P Q P R M R S T L L A L A A L C L L A V L F2

R M G H P Y K R E L P A A L P T S A P Q S T P P L P S Q P H S P E * G A R C W P W L R S A C W L C C F3

901 AGCGGATGGGCCACCCCTATAAACGGGAGCTTCCAGCAGCACTCCCCACCTCAGCCCCACAGAGCACTCCTCCGCTGCCCTCCCAGCCCCACAGCCCAGAATGAGGAGCACGCTGCTGGCCCTGGCTGCGCTCTGCCTGCTGGCTGTGCT 1050

----:----|----:----|----:----|----:----|----:----|----:----|----:----|----:----|----:----|----:----|----:----|----:----|----:----|----:----|----:----|

A P G * R G G Q C C E A L R E R L R Q S H R L H L R R L S V E E G S G * L P V P A W * V L I N A L P F1

H Q A E G E G N A V K L C G R D F V R A I V F T C G G S R W K R D L A N Y Q Y L L G K Y * L M H Y L F2

T R L K G R A M L * S S A G E T S S E P S S S P A A A L G G R G I W L I T S T C L V S T N * C T T Y F3

1051 GCACCAGGCTGAAGGGGAGGGCAATGCTGTGAAGCTCTGCGGGAGAGACTTCGTCAGAGCCATCGTCTTCACCTGCGGCGGCTCTCGGTGGAAGAGGGATCTGGCTAATTACCAGTACCTGCTTGGTAAGTACTAATTAATGCACTACCT 1200

----:----|----:----|----:----|----:----|----:----|----:----|----:----|----:----|----:----|----:----|----:----|----:----|----:----|----:----|----:----|

I T L L S T N A G Q A Y P S A G C A W L * G G * G G A D Q L Q V L S W E V H P E H S S T G F T L R Q F1

S P C Y Q L M Q G R L I P V L G V P G C E G A E E E Q T S C R C C P G R Y T P S T A A Q A S P * D S F2

H L A I N * C R A G L S Q C W V C L A V R G L R R S R P A A G A V L G G T P R A Q Q H R L H P E T A F3

1201 ATCACCTTGCTATCAACTAATGCAGGGCAGGCTTATCCCAGTGCTGGGTGTGCCTGGCTGTGAGGGGGCTGAGGAGGAGCAGACCAGCTGCAGGTGCTGTCCTGGGAGGTACACCCCGAGCACAGCAGCACAGGCTTCACCCTGAGACAG 1350

----:----|----:----|----:----|----:----|----:----|----:----|----:----|----:----|----:----|----:----|----:----|----:----|----:----|----:----|----:----|

H S F V Y V N M * P V L P H G L L S * I H V L L C * V E K V W I R V L Q V I A R N * E L G P R L W Q F1

T A S C T * I C D L C F L T A C S L R S T C C C A K W K R Y G S G C C K S L R G T E S * V Q G Y G R F2

Q L R V R E Y V T C A S S R L A L L D P R A V V L S G K G M D P G A A S H C E E L R A R S K V M A G F3

1351 CACAGCTTCGTGTACGTGAATATGTGACCTGTGCTTCCTCACGGCTTGCTCTCTTAGATCCACGTGCTGTTGTGCTAAGTGGAAAAGGTATGGATCCGGGTGCTGCAAGTCATTGCGAGGAACTGAGAGCTAGGTCCAAGGTTATGGCAG 1500

----:----|----:----|----:----|----:----|----:----|----:----|----:----|----:----|----:----|----:----|----:----|----:----|----:----|----:----|----:----|

D T P V L * C V R V R Q K P C C S * H V L H L H P E G H T P L S H S N P E T I * P I * E Y E D * A Y F1

T P L C Y D V Y V S D R S L A A V S T C C T F I P R G T H L F P T Q T Q K L Y D Q F E N M R I K H T F2

H P C A M M C T C Q T E A L L Q L A R V A P S S R G A H T S F P L K P R N Y M T N L R I * G L S I Q F3

1501 GACACCCCTGTGCTATGATGTGTACGTGTCAGACAGAAGCCTTGCTGCAGTTAGCACGTGTTGCACCTTCATCCCGAGGGGCACACACCTCTTTCCCACTCAAACCCAGAAACTATATGACCAATTTGAGAATATGAGGATTAAGCATAC 1650

----:----|----:----|----:----|----:----|----:----|----:----|----:----|----:----|----:----|----:----|----:----|----:----|----:----|----:----|----:----|

R M * D P Y S A V T F P H R C V L S Y C M R C W K A V L * * Q * L E E K E D G * G R * Q E Q G L P T F1

E C E T R T L Q * P S H T A V Y S A I A C A A G K L Y S D D N N W R K K K M G E A D D R S R G C P H F2

N V R P V L C S N L P T P L C T Q L L H A L L E S C T L M T I T G G K R R W V R Q M T G A G A A H I F3

1651 AGAATGTGAGACCCGTACTCTGCAGTAACCTTCCCACACCGCTGTGTACTCAGCTATTGCATGCGCTGCTGGAAAGCTGTACTCTGATGACAATAACTGGAGGAAAAAGAAGATGGGTGAGGCAGATGACAGGAGCAGGGGCTGCCCACA 1800

----:----|----:----|----:----|----:----|----:----|----:----|----:----|----:----|----:----|----:----|----:----|----:----|----:----|----:----|----:----|

F S T Q P H L P V W T T L L Q K Q L P T E K A M P S A T C Y Q R A E S T A A E * S Q I A P L A V * A F1

F P R S H I C L F G Q H C C R S S F Q Q K K Q C H L Q R A T R E Q K A L L L N E A R * P L W L Y E L F2

F H A A T S A C L D N T V A E A A S N R K S N A I C N V L P E S R K H C C * M K P D S P S G C M S C F3

1801 TTTTCCACGCAGCCACATCTGCCTGTTTGGACAACACTGTTGCAGAAGCAGCTTCCAACAGAAAAAGCAATGCCATCTGCAACGTGCTACCAGAGAGCAGAAAGCACTGCTGCTGAATGAAGCCAGATAGCCCCTCTGGCTGTATGAGCT 1950

----:----|----:----|----:----|----:----|----:----|----:----|----:----|----:----|----:----|----:----|----:----|----:----|----:----|----:----|----:----|

A D V C A Q T M R P R C F E R T * L E A Q H K * T S R S L G A G F V S M C V F Q E T T S C S S S I L F1

Q T S V P R Q C A H A V L N A H N * K H N I S E H P G V W G P G L L A C V C F R K P Q A V L V P S S F2

R R L C P D N A P T L F * T H I I R S T T * V N I Q E F G G R V C * H V C V S G N H K L F * F H P R F3

1951 GCAGACGTCTGTGCCCAGACAATGCGCCCACGCTGTTTTGAACGCACATAATTAGAAGCACAACATAAGTGAACATCCAGGAGTTTGGGGGCCGGGTTTGTTAGCATGTGTGTGTTTCAGGAAACCACAAGCTGTTCTAGTTCCATCCTC 2100

----:----|----:----|----:----|----:----|----:----|----:----|----:----|----:----|----:----|----:----|----:----|----:----|----:----|----:----|----:----|

G R P D I S A R L R G T T S N S Q L L I P R S R E Q R E L P V L V P G E R R F R R A L P I R S T V A F1

E G Q T S A P G * E A P L L T L S F * F H A A E S S E S S P S S S Q E S G A S A E P S P F A A Q W L F2

K A R H Q R Q A E R H H F * L S A S N S T Q Q R A A R A P R P R P R R A A L P Q S P P H S Q H S G * F3

2101 GGAAGGCCAGACATCAGCGCCAGGCTGAGAGGCACCACTTCTAACTCTCAGCTTCTAATTCCACGCAGCAGAGAGC**AG**CGAGAGCTCCCCGTCCTCGTCCCAGGAGAGCGGCGCTTCCGCAGAGCCCTCCCCATTCGCAGCACAGTGGCT 2250

----:----|----:----|----:----|----:----|----:----|----:----|----:----|----:----|----:----|----:----|----:----|----:----|----:----|----:----|----:----|

E G R * * A E P G G Q S A G G A R R A A Q Q E G G G A Q E A R G G Q T A H H V L L Q R R L Q * E G H F1

R A A D E Q S Q E G R A Q E E R D V Q R S R K V A V L K R R E V A K L L T T S C C S V G C S E R D I F2

G P L M S R A R R A E R R R S A T C S A A G R W R C S R G A R W P N C S P R P A A A S A A V R G T S F3

2251 GAGGGCCGCTGATGAGCAGAGCCAGGAGGGCAGAGCGCAGGAGGAGCGCGACGTGCAGCGCAGCAGGAAGGTGGCGGTGCTCAAGAGGCGCGAGGTGGCCAAACTGCTCACCACGTCCTGCTGCAGCGTCGGCTGCAGTGAGAGGGACAT 2400

----:----|----:----|----:----|----:----|----:----|----:----|----:----|----:----|----:----|----:----|----:----|----:----|----:----|----:----|----:----|

Q P A V L T Q L L P G G F E A I A * R Y * S G H D I K H G P F S R V L * L Y L Y S F K Y A E G R R L F1

S L L C * R S C C R G G S R P L L E G I K A V M T * S T V L S V V F F S C T F I L S S M R R G E G * F2

A C C A N A A A A G G V R G H C L K V L K R S * H K A R S F Q S C S L A V P L F F Q V C G G A K A E F3

2401 CAGCCTGCTGTGCTAACGCAGCTGCTGCCGGGGGGGTTCGAGGCCATTGCTTGAAGGTATTAAAGCGGTCATGACATAAAGCACGGTCCTTTCAGTCGTGTTCTTTAGCTGTACCTTTATTCTTTCAAGTATGCGGAGGGGCGAAGGCTG 2550

----:----|----:----|----:----|----:----|----:----|----:----|----:----|----:----|----:----|----:----|----:----|----:----|----:----|----:----|----:----|

S V R S C N A T R V G L L G F Y K A R Q C F S I A I L D V S T N I S S H L T P V L A H E K P * H S E F1

V L G A A M Q L G W V C W D S T K H D S V L A L Q S * T * V Q I S P P T S L L S W H M R S R N I A S F2

C * E L Q C N S G G F V G I L Q S T T V F * H C N L R R E Y K Y L L P P H S C L G T * E A V T * R A F3

2551 AGTGTTAGGAGCTGCAATGCAACTCGGGTGGGTTTGTTGGGATTCTACAAAGCACGACAGTGTTTTAGCATTGCAATCTTAGACGTGAGTACAAATATCTCCTCCCACCTCACTCCTGTCTTGGCACATGAGAAGCCGTAACATAGCGAG 2700

----:----|----:----|----:----|----:----|----:----|----:----|----:----|----:----|----:----|----:----|----:----|----:----|----:----|----:----|----:----|

L Q K P Q S S F W T R V S P A R Q G * Q * A C A G T T P S S W L C A G R R S V G A A A A A H T A T P F1

Y K N P R A A F G P G Y H P R D R G S S K H V P V P R R A R G S V P G G A A W G L Q Q Q L T Q R P L F2

T K T P E Q L L D P G I T R E T G V A V S M C R Y H A E L V A L C R A A Q R G G C S S S S H S D P S F3

2701 CTACAAAAACCCCAGAGCAGCTTTTGGACCCGGGTATCACCCGCGAGACAGGGGTAGCAGTAAGCATGTGCCGGTACCACGCCGAGCTCGTGGCTCTGTGCCGGGCGGCGCAGCGTGGGGGCTGCAGCAGCAGCTCACACAGCGACCCCT 2850

----:----|----:----|----:----|----:----|----:----|----:----|----:----|----:----|----:----|----:----|----:----|----:----|----:----|----:----|----:----|

L S A L E T R R R S R D L S P V Y R T S C P L R G H F A A G Q M G N T P L S S E R C I S P V I F L A F1

C L P S K H A G G A V I Y L P F T G R P A R C A V T L Q L G R W A I R L * A Q R D A F L L L S F * L F2

V C P R N T Q E E P * F I S R L P D V L P A A R S L C S W A D G Q Y A S E L R E M H F S C Y L S S F F3

2851 CTGTCTGCCCTCGAAACACGCAGGAGGAGCCGTGATTTATCTCCCGTTTACCGGACGTCCTGCCCGCTGCGCGGTCACTTTGCAGCTGGGCAGATGGGCAATACGCCTCTGAGCTCAGAGAGATGCATTTCTCCTGTTATCTTTCTAGCT 3000

----:----|----:----|----:----|----:----|----:----|----:----|----:----|----:----|----:----|----:----|----:----|----:----|----:----|----:----|----:----|

F C L L H G H T M G L C M S R A A V A W G R G * A A P S C G L V P D A F V L P S K K H S V I S W R I F1

S A F C M A T Q W D S A * A G Q R S H G A G A E P P H R A G W F Q M H S F C L L K S T L * F L G G S F2

L P S A W P H N G T L H E Q G S G R M G P G L S R P I V R V G S R C I R F A F * K A L C D F L E D H F3

3001 TTCTGCCTTCTGCATGGCCACACAATGGGACTCTGCATGAGCAGGGCAGCGGTCGCATGGGGCCGGGGCTGAGCCGCCCCATCGTGCGGGTTGGTTCCAGATGCATTCGTTTTGCCTTCTAAAAAGCACTCTGTGATTTCTTGGAGGATC 3150

----:----|----:----|----:----|----:----|----:----|----:----|----:----|----:----|----:----|----:----|----:----|----:----|----:----|----:----|----:----|

M H S R E I Q G E K V L Q G H P V L K A A K G A K W H P T E D H S P E K A L G N T V M A Q G N H A S F1

C T H G K F K E K K C Y R D T Q C * K Q L K V Q N G I L Q K I T R R R K P W G I Q * W L R G T T L L F2

A L T G N S R R K S V T G T P S A E S S * R C K M A S Y R R S L A G E S P G E Y S D G S G E P R F * F3

3151 ATGCACTCACGGGAAATTCAAGGAGAAAAAGTGTTACAGGGACACCCAGTGCTGAAAGCAGCTAAAGGTGCAAAATGGCATCCTACAGAAGATCACTCGCCGGAGAAAGCCCTGGGGAATACAGTGATGGCTCAGGGGAACCACGCTTCT 3300

----:----|----:----|----:----|----:----|----:----|----:----|----:----|----:----|----:----|----:----|----:----|----:----|----:----|----:----|----:----|

K Q V N V V L V L H C C S Q Q Q E D S G * N S F L L L L K A A T W L F K I X F1

S R * M L S W S F T A A L S N R R T Q A E T H F S C C * K Q R P G Y S K * X F2

A G E C C L G P S L L L S A T G G L R L K L I S P A A K S S D L A I Q N R F3

3301 AAGCAGGTGAATGTTGTCTTGGTCCTTCACTGCTGCTCTCAGCAACAGGAGGACTCAGGCTGAAACTCATTTCTCCTGCTGCTAAAAGCAGCGACCTGGCTATTCAAAATAGA 3413

----:----|----:----|----:----|----:----|----:----|----:----|----:----|----:----|----:----|----:----|----:----|---
