# Supplementary material for: Sequence properties of certain GC rich avian genes, their origins and absence from genome assemblies: case studies
Source: BMC Genomics. 2019 Oct 14;20:734. doi: 10.1186/s12864-019-6131-1 (PMC6792250; doi:10.1186/s12864-019-6131-1)
Supplement: Supplementary file 5 — Additional file 5. Genomic sequences of the avian leptin gene [file 12864_2019_6131_MOESM5_ESM.docx]

**a. Coding exons of the leptin gene in the zebrafinch (*Taeniopygia guttata)* genome (76.51% GC content) - KJ196274.1 , positions 3464 to 4663.**

100

| | | | | | | | | | | |

ctggggtcccaaggcccacccccgtggtcacggccgtgccgcgggtggcggctgccaaagggctccggcggggacacggtggcaccgcgacacggtgacgagggctccggagcccgtgtg

200

| | | | | | | | | | | |

ccgccggcgccgcttcccgcacgtcccctgccccgtccctggtgacaccggccacccctcagcctgccgggggtggcggtgacacgcgtgtcccctcccgggggcgcggagccgccggtg

300

| | | | | | | | | | | |

accgccgcgtccccgtgcccagagcggcggg**ATG**CGGTGTCCCGGCGTGTCGCTGTGGGGCCTGCTGTGCCTGGGGGCGGCGGCGGGCGGGGGTCGCCCCGTGCGGCTCGAGGGGCTCCG

*M R C P G V S L W G L L C L G A A A G G G R P V R L E G L R*

400

| | | | | | | | | | | |

AGCGGACGCGCGGGCCCTGACCCGGACCCTCAGCACGCGCCTGCAGCAGCTCCAG**GT**gaccccggcccggggaccgcggggacaccggggagggggctgggacacagccgggacaccccc

*A D A R A L T R T L S T R L Q Q L Q*

500 600

| | | | | | | | | | | |

ggagggacccccagagcggggggacgttcccagactcgggggctgctcctcagtacggggggatcctgttctccaaagtggggtggggtccccgggggggtctcccatgggggggatccc

700

| | | | | | | | | | | |

gtgtccagatttggggggtccccagggaggggcgcctggggctgggtgggcgtgggggctctggtcccaaaccgggcacccccagggatggggtctggggatctgcacccccagggatgg

800

| | | | | | | | | | | |

ggtctggaggggtctgcacccccagggatggggtctgggggtctgcacccccagggatggggtctggggatctgcacccccagggatgggctctggaggggtttgggggtctgcaccccc

900

| | | | | | | | | | | |

aggaatggggtctggaggggtctgcacccccagggatggggtctggaggggtctgcacccccagggatgggctctgggggtctgcgcccccagggatggggtctggaggggtttgggggt

1000

| | | | | | | | | | | |

ctgcgcccccagggatgggctctgggagcaccccgagggttgggggtccccaaggagggacagccatgctgggggggccctgtgccccacgctggggtccccggggaggccggcggcgct

1100

| | | | | | | | | | | |

gaccccccctgtgcccccc**AG**CTGTTCCCGCTGACCCTGCGCCTCAGCGGGCTGGAGGGGGTCCCGGAGGGGGTCCCGGAGGGGGTCCCGGAGGGGGGGGTCCCGCCGGGGCTGGGCTGG

*L F P L T L R L S G L E G V P E G V P E G V P E G G V P P G L G W*

1100 1200

| | | | | | | | | | | |

GCCGCCCAGCGGCTGCAGCTCTTCCAGCGGCTCCTGGGGGCGCTGCCCGGGCCCGACCCGCGCCTGGCCCAGGTGGCCAACGACCTGGAGAACCTGCGCAGCCTGCTGGCCCTGCTGGGG

*A A Q R L Q L F Q R L L G A L P G P D P R L A Q V A N D L E N L R S L L A L L G*

1300

| | | | | | | | | | | |

ACCCTGCTGGGCTGCCCCCCGCCCCGCGACCCCCGCCCGCCGCCCCCCGCCCCGCTGGCCGAGGCTCCGCACACCGTGGCCGGGGTGGCCCTGGCGCGGCTCCGCCGCTGCCTGGACGGG

T L L G C P P P R D P R P P P P A P L A E A P H T V A G V A L A R L R R C L D G

1400

| | | | | | | | | | | |

GTCGCCGCCTGCCTGGAGGGGGTCCCCGCGTGT**TAG**ggacccccgggacccccgggacccccgggcccacagggacagcccgggacccccgcggggtctcgtcaggacccacagacagga

*V A A C L E G V P A C*

Nucleic acid sequence model of the region containing the coding exons split by a 685 bp intron in the zebrafinch leptin gene. The start and stop codons and the dinucleotides at the RNA splicing site are shown in bold and indicated in blue and red respectively. The protein sequence of the zebrafinch leptin is shown in italics, below the nucleic acid sequence of the coding regions. Statistically significant blocks in direct repeats were identified with MEME at http://meme-suite.org/tools/meme and are highlighted in yellow and grey respectively. Motifs matching with the consensus of G-quadruplex structures (G_3_+_N1-12_G_3_+N_1-12_G_3_+N_1-12_G_3_) are underlined and involved guanine stretches are shown in red.

**b. Coding exons of the leptin gene in the wavy parakeet (*Melopsittacus undulatus*) genome (70,27% GC content) - KJ196275.1, positions 6301 to 6756.**

100

| | | | | | | | | | | |

cctcaccccctttccccccttgtccccatctctcccctttccccccctgtccccctttgtcctcccccatccccatgtgttccacatgtcccccaccctatgcccgccccaggggctcac

200

| | | | | | | | | | | |

tcccttctctcattcccccccgagtctcctcggtccggtttggggtccccccccgtgtcccagggccggtgcc**ATG**TGGGGCCCGGCGGCTCCGCTCTGGGCGCTGCTCTGCCTGGGGCT

*M W G P A A P L W A L L C L G L*

300

| | | | | | | | | | | |

GCCCGCGGCCGCGGGGCGTCCCGTGGGGCTGGACAGGGTGCGGGTGGACGCGAGGAACCTGATCCGCACCCTCAGCACCCGCCTCCAGCGCCTGCAG**GT**gagggggggcccaacagggac

*P A A A G R P V G L D R V R V D A R N L I R T L S T R L Q R L Q*

400

| | | | | | | | | | | |

cccggtcaggcaaggggaggggggagcctcggcttgctccatggggggcacccagaatggatgaggggagatgggggggcccgagggggggatatggaaggagggggggtctgctcctaa

500 600

| | | | | | | | | | | |

attgtgggtccgggggggggggggctctgaggcacagattgggggtccttgtgggtgggatgctggactgggggtccccaggggtccatttaatgggggttatctgtgctctcacggggg

700

| | | | | | | | | | | |

gttccaggggaaaggtccccgtggctgtaggggttagggggtgctctgaggcagggattggaggtcctcgaggggggaggctatctacggggtccccataggggatccctattgctgagg

800

| | | | | | | | | | | |

ggtctgggtgggagggctctgtacccttaataggggtccccaagtggggtggtctgtgcccaaactcggggtctccaggaggggtccccattgctgtgtgtggtggtggggtctctgggc

900

| | | | | | | | | | | |

ccagatctggggtacccaaggaggggggctgtgcccctagacaccggtctcaggaggggtatccccatggttggggggggcgtccatgcccagatagggggttccaaaggcgagtttgtt

1000

| | | | | | | | | | | |

tgtggtgggggggtattcccatggctggggaggggggttgggggtgtgtctatgccctggatagggatgttcccaaggggtagatgctgggagaggggatccccatgcccagatgtgggg

1100 1200

| | | | | | | | | | | |

tccccaaggctgaggaagctgagtgggggctcagtgacctggatggtgggggacccccataactggggggtgtctatggggtgtctacggcggtatctatgggggtgtctctgggggtgt

1300

| | | | | | | | | | | |

ctgtgggtgctgacccctctgtgttcccccctcccgc**AG**CTGTTCCCGCTGGGGGTGCAGATCCTCGGGCTGGGGGCGGTGCTGGAGGGGCCGCCCCCCCCGCTGGGGCTGGGGGCCATG

*L F P L G V Q I L G L G A V L E G P P P P L G L G A M*

1400

| | | | | | | | | | | |

GGGCAGCACCTGCAGCTGTTCCAGCGCCTGCTGGGGGCGCTGCCGGGCGGGGGGGCCGCGGGGGGGGCTCCGCTGGCGCAGGTGGCCACGGACCTGGAGACCCTGCGGCACCTGCTGGGG

1500

G Q H L Q L F Q R L L G A L P G G G A A G G A P L A Q V A T D L E T L R H L L G

| | | | | | | | | | | |

GCCATGGGCGCGCTCCTGCGCTGCCCCCCCCCGCCCCCGCCCCCCCCCGCGCCGCCCCCCGCACCCCCCGCCGTGGCCGAGGCCCCCCACACGGCGGCAGGGGTGACCCTGGCCCGGCTG

*A M G A L L R C P P P P P P P P A P P P A P P A V A E A P H T A A G V T L A R L R*

1600

| | | | | | | | | | | |

CGCCGCTGCCTCGATGTGCTGGAGAGGGGGCTGGAGGGGGGGGACATGGGGTGT**TAA**tgggggggagagggggaatacgtggggacaatgtgggggagccaggatgttgatggggcgaga

*R C L D V L E R G L E G G D M G C*

1700 1800

| | | | | | | | | | | |

tgggacacaggggacatggggtgttaatggggggggacatggagggacatggggtgttaataggggggacatggagggacatggggtgctaatggggggggacacggagggacatggggt

Nucleic acid sequence model of the region containing the coding exons split by a 901 bp intron in the zebrafinch leptin gene. The start and stop codons and the dinucleotides at the RNA splicing site are shown in bold and indicated in blue and red respectively. The protein sequence of the zebrafinch leptin is shown in italics, below the nucleic acid sequence of the coding regions. Statistically significant direct repeats were identified with MEME at http://meme-suite.org/tools/meme and are highlighted in yellow. Motifs matching with the consensus of G-quadruplex structures (G_3_+_N1-12_G_3_+N_1-12_G_3_+N_1-12_G_3_) are underlined and involved guanine stretches are shown in red

**c. Coding exon 1 and 5' end of the coding exon 2 of the leptin gene in the genome of collared flycatcher, *Ficedula albicollis* (78.71 % GC content)*.***

100

| | | | | | | | | | | |

ggcccgcgggacacgcggggacggcgcatccccgggacgggtg**ATG**TCACCAGGGACGGGACAGGGGACACGAGCGGGGCACGCGGGAGGTGGCGCCGCTGGCGCAGGGCCGTGGGCGCT

*M S P G T G Q G T R A G H A G G G A A G A G P W A L*

| | | | | | | | | | | |

GCTGTGGCTGCTGGTGGCCGTGGCCGGGGGTCGCCCCGTGCGGCTCGAGCGGGTCCGGGCGGACGCGCGGGCCCTGACCCGGACCCTGAGCGCGCGCCTGCAGCAGCTCCAG**GT**Gacccc

*L W L L V A V A G G R P V R L E R V R A D A R A L T R T L S A R L Q Q L Q*

200

| | | | | | | | | | | |

ggcccccgggtgatgtcaccagggacgggacaggggacacgagcggggcacgcgggaggtggcgcggctggcgcagggaccgcggagcgccggcggccccgtggcNNNNNNNNNNNNNNN

300

| | | | | | | | | | | |

NNNNNNNNNNNNNNNNNNNNNNNNNNNNNNNNNNNNNNNNNNNNNNNNNNNNNNNNNNNNNNNNNNNNNNNNNNNNNNNNNNNNNNNNNNNNNNNNNNNNNNNNNNNNNNNNNNNNNNNN

400

| | | | | | | | | | | |

gatctagcccagccccggggggagccccccgtccgggagccccccctcggggaccccctccaggccggtgacgcgcagggtcagcgggaacagctgcggggatctcccgcAGCTGTTCCC

500 600

| | | | | | | | | | | |

GCTGACCCGGCGCGTCACCGGCCTGGAGGGGGTCCCCGAGGGGGGGCTCCCGGACGGGGGGCTCCCCCCGGGGCTGGGCTGGGCTGCCCACCGCCTCC**AG**CTCTTCCAGCGCCTCCTGGG

*L F Q R L L G*

| | | | | | | | |

CGCCCTGGCCGCGGGCGACCTGCGCCTGGCCCAGGTGGCCAACGACCTGGAGAACCTGCGCAGCCTCCTGGGCGCGCGGGGGACCCCGCTA

*A L A A G D L R L A Q V A N D L E N L R S L L G A R G T P L*

Nucleic acid sequence model of the region containing the coding exons split by intron with inner region that remained to elucidate in the leptin gene of the collared flycatcher. The start and stop codons and the dinucleotides at the RNA splicing site are shown in bold and indicated in blue and red respectively. The protein sequence of the leptin is shown in italics, below the nucleic acid sequence of the coding regions. Motifs matching with the consensus of G-quadruplex structures (G_3_+_N1-12_G_3_+N_1-12_G_3_+N_1-12_G_3_) are underlined and involved guanine stretches are shown in red. Our searches using Illumina reads of the mallard duck (*Anas platyrhynchos*) genome sequencing project to reconstruct the genomic copy of the leptin gene was in vain.

**d. Coding exons of the leptin gene in the genome of the bald eagle, *Haliaeetus leucocephalus* (72.27% GC content) - JPRR01009366, positions 773 to 1980, inverse complementary strand.**

100

| | | | | | | | | | | |

CTCTGTGTCCCCTCTCGGGGAGAGGTGTGTGTGGCATTTTTGGGGTGCAGGGGACCCCCCCCCGAGGCACCTGACACCCCCGTGCCACCCGGCGCAGAGCCCAGCAGG**ATG**CGGTGGCCC

*M R W P*

*200*

| | | | | | | | | | | |

AGTGTGTCCCTCTGGAGTCTCCTCTGGCTGTGGGTGCCGCTGGCCAGTGGCCGTCCCGTCCGGCTGGAGAAGGTCCGGGCGGACACCAGGAACCTCACCCGCCCCCTCAGCACCCGCATC

*S V S L W S L L W L W V P L A S G R P V R L E K V R A D T R N L T R P L S T R I*

300

| | | | | | | | | | | |

CAGCAGCTGCAG**GT**GAGGCCCGGGGGGGGGGGAACGGGACGGGCGGGGGGGGTTGGGGGGTGTCCCCCCCCCTCCCAGGGCCACCCCATAGGGTTCACTGCATGCCCCATGTCACCCAAG

*Q Q L Q*

400

| | | | | | | | | | | |

GGAGGGGGCTGGGCTGTGAGGTGGGGGGCCCTGCGCCCCAGGGTGGTCGCTGTGAGGGGGGACACGATGACACCCAGCAGAGGGTGGGGGGGCTCTGTGCTCCAGACTGGGGGAGGGAGT

500 600

| | | | | | | | | | | |

CCCCAGAGAGGGACACCTATGGCTGGGGTGGGGGACCCTGTGCCCCAAGGGATGCCCATGGCTGGGGGTGGGGGGCTCTGTGCCCCAGATGGGGGGTCCCCGAGGAGGGATGCTGGGGGG

700

| | | | | | | | | | | |

GTGTCTGTGCCCCAGATGAGGGGGGGTCCCAGGGAGGGATGCCCATGACTGGGTGGGGGACCCTGTGCCCCAAGGGATGCCCGGGGCTGAGTGTGAGGGGGTCTCTACACCCCAGCTGGG

800

| | | | | | | | | | | |

GAGGGGGGGTCCCCAAGGAAGGTCTCCCACAGCAGGTGGGGCAGGACCCCTGCCCGAGGAGCGGGCAGTGGGTGCCATGCCTTCCCCCCCCCCCCCTC**AG**CTCTTCCCCCTGAGCCTGAA

*L F P L S L K*

900

| | | | | | | | | | | |

GATCAGCGGGCTGGAGGCCATCCCGGGGGAGGGGGCTCCCGAGGGGCTGGGGGCCATGGACCACCGTCTCCAGCTCTTCCAGCGCCTGCTGGGCGGCCTGGCGGCCGGCAACCTACCGCT

*I S G L E A I P G E G A P E G L G A M D H R L Q L F Q R L L G G L A A G N L P L*

1000

| | | | | | | | | | | |

TGCCCAGATTGCCAACGACATGGAGAACCTCCGCAGCCTCCTGGCCGCCCTGGCCACCCACCTGGGCTGTCCCCCGCTCCGCACCCCCCCGGGGCCCCCGGGACCCCCCGGTTTATCCGA

*A Q I A N D M E N L R S L L A A L A T H L G C P P L R T P P G P P G P P G L S D*

1100 1200

| | | | | | | | | | | |

CTTGCTGGTCGAAGCACCCCACACTGCCGCCGGGCTGGCCCTGGCGCGGCTTCGCGTTTGCCTGGACGGCATCGCCGCCCGCCTCGATGGCCTCCCTGCCTGC**TAG**GCATGCCCTGGGGA

*L L V E A P H T A A G L A L A R L R V C L D G I A A R L D G L P A C*

|

CCCCAGCC

Nucleic acid sequence model of the region containing the first coding exon in the bald eagle leptin gene. The start and stop codons and the dinucleotides at the RNA splicing site are shown in bold and indicated in blue and red respectively. The protein sequence of the leptin is shown in italics, below the nucleic acid sequence of the coding regions. Statistically significant direct repeats were identified with MEME at http://meme-suite.org/tools/meme are highlighted in yellow. Motifs matching with the consensus of G-quadruplex structures (G_3_+_N1-12_G_3_+N_1-12_G_3_+N_1-12_G_3_) are underlined and involved guanine stretches are shown in red.

**e. Coding exons of the leptin gene in the genome of the golden eagle, *Aquila chrysaetos canadensis* (73.61% GC content) - JRUM01013750.1, positions 7837 to 9045, inverse complementary strand.**

100

| | | | | | | | | | | |

CTCCGTGTCCCCTCTCGGGGGGGGGGGGGTGGCATTTTTGGGGTGCAGGGGACCCCCCCCCGAGACACCTGACACCCCCGTGCCGCCCAGCGCAGAGCCCAGCAGG**ATG**CGGTGGCCCGG

*M R W P G*

*200*

| | | | | | | | | | | |

CGTGTCCCTCTGGGGTCTCCTCTGGCTCTGGGTGCCGCTGGCCGGTGGCCGTCCCGTCCGGCTGGAGAAGGTCCGGGCGGACACCAGGAACCTCACCCGCACCCTCAGCACCCGCATCCA

*V S L W G L L W L W V P L A G G R P V R L E K V R A D T R N L T R T L S T R I Q*

300

| | | | | | | | | | | |

GCAGCTGCAG**GT**GAGACCGGGGGGGACACAGATGGGGGGGCTTGGGGGGTGTCCCCCCTCCTCCCAGGGCCACCCCATAGGGTTCACTGCATGCCCCATGTCACCCAAGGGAGGGGGCTG

*Q L Q*

400

| | | | | | | | | | | |

GGCTGTGAGGTGGGGGGCCCTGCGCCCCAGGGTGGTCGCTGCGGGGGGGACGACGATGACACCCAGCACGGGGTGGGGGGCTCTGTGCTCCAGATTGGCGGGGGGGGGAGTCCCCAGAGA

500 600

| | | | | | | | | | | |

GGGACACCCATGGCTGGGGTGGGGGACCCTGTGCCCCAAGGGATGCCCATGGCTGGGGGGGGAGGCTCTGTGCCCCAGATGGGGGGTCCCTGAGGAGGGATGCTGGGGGGGGGGGGGGCT

700

| | | | | | | | | | | |

CTGTGCCCCAGATGAGGGGGGTTCCCAGGGAGGGATGCCCATGACTGGGTGGGGGACCCTGTGCCCCAAGGGATGCCCGGGGCTGAGTGTGGGGGGGGTCTCTACACCCCAGCTGGGGAG

800

| | | | | | | | | | | |

GGGGTTCCCCAAGGAGGGTCTCCCACAGCAGGTGGGGCAGGACCCCTGCCCGAGGAGCGGGCAGTGGGTGCCATGCCTTCACCCCCCCCCGCCCCCCCC**AG**CTCTTCCCCCTGAGCCTGA

*L F P L S L K*

900

| | | | | | | | | | | |

AGATCAGCGGGCTGGAGGCCATCCCGGGGGAGGGGGCTCCCGAGGGGCTGGGGGCCATGGACCACCGCCTCCAGCTCTTCCAGCGCCTGCTGGGCGGCCTGGCGGCCGGCAACCTGCCGC

*I S G L E A I P G E G A P E G L G A M D H R L Q L F Q R L L G G L A A G N L P L*

1000

| | | | | | | | | | | |

TGGCCCAGATCGCCAACGACATGGAGAACCTCCGCAGCCTCCTGGCCGCCCTGGCCACCCACCTGGGCTGCCCCCTGCCCCGCACCCCCCCGGGACCCCCGGGACCCCCCGGTTTATCCG

*A Q I A N D M E N L R S L L A A L A T H L G C P L P R T P P G P P G P P G L S D*

1100 1200

| | | | | | | | | | | |

ACTTGCTGGTCGAAGCACCCCACACCGCCGCCGGGCTGGCCCTGGCGCGGCTTCGCGTTTGCCTGGACGGCATCGCCGCCCGCCTCGACGGCCTCCCCGCCTGC**TAG**GGACCCCCTGGGG

*L L V E A P H T A A G L A L A R L R V C L D G I A A R L D G L P A C*

|

ACCCCAGCC

Nucleic acid sequence model of the region containing the first coding exon in the golden eagle leptin gene. The start and stop codons and the dinucleotides at the RNA splicing site are in bold and indicated typed in blue and red respectively. The protein sequence of the leptin is shown in italics, below the nucleic acid sequence of the coding regions. Statistically significant direct repeats were identified with MEME at http://meme-suite.org/tools/meme are highlighted in yellow. Motifs matching with the consensus of G-quadruplex structures (G_3_+_N1-12_G_3_+N_1-12_G_3_+N_1-12_G_3_) are underlined and involved guanine stretches are shown in red.

**f. Coding exons of the leptin gene in the genome of the double-crested cormorant, *Nannopterum auritus* (73.43% GC content) - NFMC01000563.1, positions 7837 to 87157 to 88530, inverse complementary strand.**

| | | | | | | | | | | |

CGGGGTCGGAGCACCCCTNGGGGGGGGGGGGGGGGGGGGGGGGGGGGGGACACGGGTGGCATTTCGGGGGTGCCCTGACCCCGCGCCCCCCCGGCGCAGAGCCCGGCGCC**ATG**CGCGGCC

*M R G P*

| | | | | | | | | | | |

CCGCCGTGTCCCCCTGGGGTCTCCTCTGGCTGTGGCTCCCGCTGGCCGCCGGCCGCCCCGGCCGCCTCGACAAGGTGCAGGCCGACACCCGCAACCTCACCCGCACCCTCAGCGCCCGCA

*A V S P W G L L W L W L P L A A G R P G R L D K V Q A D T R N L T R T L S A R I*

| | | | | | | | | | | |

TCCAGCAGCTCCAG**GC**GAGCCCCGGGGGGGGGGACACACACACACCCCCCCCACCCACCCAGGGACCCCCCACCCACCCACCCAGGGAGCGGCGGGGGGCCGGGGCGCTGCAGCCGCGTC

*Q Q L Q*

| | | | | | | | | | | |

CCGTCTCACCCGAGGGAGGGGGCGGTGTCTGTGCCTCAGGGGGGTCCCTGGGGGGGGACACACCCAGTTCAGGGGTGGGGGGGGGGCTGTGATCCGGGTGGGGGGTGTCTCCAGGGAGGG

| | | | | | | | | | | |

GCAGCTGGCACAGGGTGTCCCCCACATGGAGAAGGGTGTCCCCAGGGCTGGGTGGGATTGGGGGGGGGACGACTCGATGCATAGTTGGNNNNNNNNNNNNNNNNNNNNNNNNNNNNNNNN

| | | | | | | | | | | |

NNNNNNNNNNNNNNNGGGGTGGGGTTGGGGGGCTCTACACCCCAAATGGGGGGGTCCCCAAGGAGATATGCCGAGGGATGGGTGGGATTTGGAGGTGAAGTGGGGGGGCCCTGTGACCTA

| | | | | | | | | | | |

GATGGGGGGGGTCACTCGAGGCTGGGTGGGATTGGGGGGTGGGGGGCTCTGTGCCCTAGATGGGAGGGTTCCCTGGGGGGCTCTACACCCCAAACGGGGGGTCCCCAGGAAGGGATGCTG

| | | | | | | | | | | |

GGGGTGGGGGCGCTCTGCACCCTGGCTGGGGGTCCCCTGGGGCGGTTGCCCAGGGCTGGGCGGGGTTGGGGGGGGGGCTCTGCGCCCCAGCGGGGGGGTCTCCCGAGGTGGGGGGCCGTG

| | | | | | | | | | | |

CCCTGACACCCCACCCCCCCTTCGCCCCCCTCC**AG**CTCTTCCCCCTGAGCCTGAAGATCAGCGGGCTGGAGGCCATCCCGGGGGAGGGGGCTCCGGAGGGGCTGGGGGCCATGGACCACC

*L F P L S L K I S G L E A I P G E G A P E G L G A M D H R*

| | | | | | | | | | | |

GCCTCCAGCTCTTCCAGCGCCTGCTGGGCCGGCTGGCGGCCGCGGGGCTACCGCTGGCCCAGATCGCCAACGACATGGAGAACCTGCGCAGCCTCCTGGGGGTCCTGGGCGCCCACCTGG

*L Q L F Q R L L G R L A A A G L P L A Q I A N D M E N L R S L L G V L G A H L G*

| | | | | | | | | | | |

GCTGCAGCCCCCCCCGCGCCCCCCCGGGCCCCCCGGCCCTGCCCGACCCCCTGGCCGAAGCCCCCCACACGGCCGCCGGGCTGGCCCTGGCACGGCTCCGCGTCTGCCTGGACGGCATCG

*C S P P R A P P G P P A L P D P L A E A P H T A A G L A L A R L R V C L D G I A*

| | | | |

CCGCCCGCCTCGACGGGCACCCCGCCTGC**TAG**GGGCACCCCGGGGGGTCCCACA

*A R L D G H P A C*

Nucleic acid sequence model of the region containing the first coding exon in the double-crested cormorant leptin gene. The start and stop codons and the dinucleotides at the RNA splicing site are shown in bold and indicated in blue and red respectively. The protein sequence of the leptin is shown in italics, below the nucleic acid sequence of the coding regions. Statistically significant direct and inverted repeats were identified with MEME at http://meme-suite.org/tools/meme and were highlighted in yellow, and in light and dark grey, respectively. Motifs matching with the consensus of G-quadruplex structures (G_3_+_N1-12_G_3_+N_1-12_G_3_+N_1-12_G_3_) are underlined and involved guanine stretches are shown in red.

**g. Coding exons of the leptin gene in the genome of african fisher eagle, *Haliaeetus vocifer* (72.72% GC content).**

100

| | | | | | | | | | | |

CTCTGTGTCCCCTCTCGGGGAGAGGTGTGTGTGGCATTTTTGGGGTGCAGGGGACCCCCCCCCGAGGCACCTGACACCCCCGTGCCACCCGGCGCAGAGCCCGGCAGG**ATG**CGGTGGCCC

*M R W P*

200

| | | | | | | | | | | |

AGTGTGTCCCTCTGGGGTCTCCTCTGGCTGTGGGTGCCGCTGGCCAGTGGCCGTCCCGTCCGGCTGGAGAAGGTCCGGGCGGACACCAGGAACCTCACCCGCACCCTCAGCACCCGCATC

*S V S L W G L L W L W V P L A S G R P V R L E K V R A D T R N L T R T L S T R I*

300

| | | | | | | | | | | |

CAGCAGCTGCAG**GT**gaggcccgggggggggaacgggacggacggggggcgttggggggtgtgtgtccccctcccagggccaccccatagggttcactgcatgccccatgtcacccaaggg

*Q Q L Q*

400

| | | | | | | | | | | |

agggggctgggctgtgaggtggggggccctgcgccccaggatggtcgctgcggggggggccacgatgacacccagcacagggtggggggctctgtgctccagactggggggggggagtcc

500 600

| | | | | | | | | | | |

ccagagagggacacctatggctggggtgggggaccctgtgccccaagggatgcccatggctggggctgggggtggggggctctgtgccccagatgggggtccccgaggcgggatgctggg

700

| | | | | | | | | | | |

gggggtgtctgtgccccagatgaggggggctcccagggagggatgcccatgactgggtgggggaccctgtgccccaagggatgcccggggctgagtgtgagggggtctctacaccccagc

800

| | | | | | | | | | | |

tggggagggggggtccccaaggaaggtctcccacagcaggtggggcaggacccctgcccgaggagcgggcagtgggtgccatgccttcaccccccccccc**AG**CTCTTCCCCCTGAGCCTG

*L F P L S L*

900

| | | | | | | | | | | |

AAGATCAGCGGGCTGGAGGCCTTCCCAGGGGAGGGGGCTCCTGAGGGGCTGGGGGCCATGGACCACCGTCTCCAGCTCTTCCAGCGCCTGCTGGGCGGCCTGGCAGCCGGCAACCTGCCG

*K I S G L E A F P G E G A P E G L G A M D H R L Q L F Q R L L G G L A A G N L P*

1000

| | | | | | | | | | | |

CTGGCCCAGATTGCCAACGACATGGAGAACCTCCGCAGCCTCCTGGCCGCCCTGGCCACCCACCTGGGCTGTCCCCCGCTCCGCACCCCCCCGGGACCCCCGGGACCCCCCGGTTTATCC

*L A Q I A N D M E N L R S L L A A L A T H L G C P P L R T P P G P P G P P G L S*

1100 1200

| | | | | | | | | | | |

GACTTGCTGGTCGAAGCACCCCACACCGCCGCCGGGCTGGCCCTGGCGCGGCTTCGCGTTTGCCTGGACGGCATCGCCGCCCGCCCCGACGGCCTCCCCGCCTGC**TAG**GGATGCCCCGGG

*D L L V E A P H T A A G L A L A R L R V C L D G I A A R P D G L P A C*

|

GACCCCAGCC

Nucleic acid sequence model of the region containing the coding exons split by intron with inner region that remained to elucidate in the leptin gene of the african fisher eagle. The start and stop codons and the dinucleotides at the RNA splicing site are shown in bold and indicated in blue and red respectively. The protein sequence of the leptin is shown in italics, below the nucleic acid sequence of the coding regions. Motifs matching with the consensus of G-quadruplex structures (G_3_+_N1-12_G_3_+N_1-12_G_3_+N_1-12_G_3_) are underlined and involved guanine stretches are shown in red. Statistically significant direct and inverted repeats were identified with MEME at http://meme-suite.org/tools/meme and are highlighted in yellow, and in grey and black respectively.

**h. Second coding exon of the leptin gene in the genome of black-chinned hummingbird, *Archilochus alexandri* (82,49 % GC content).**

100

| | | | | | | | | | | |

GTGGGGGGGGCCAGCGAGAGAGGGGGGGGGGGCCGGCCCCCCCGGGGCCCCTTTGGGGGGGGGGCCCCCGGGGGGGTACGGGCCCCCCCCCCCTTCCCCCCCCCCCGTTTTTCCCCCCCC

200

| | | | | | | | | | | |

CCCCCCCCCCCCCGC**AG**CTCTTCCCTCCGGGCCCTGACCTGGGGGGGCTGCCGGGGGTGCCTGGGGGGGGGTCCGCAGGCGGGGGGGGCCTGGGGGCCATGGCCCAGCGCCTCCTCCTCT

*L F P P G P D L G G L P G V P G G G S A G G G G L G A M A Q R L L L F*

300

| | | | | | | | | | | |

TCCAGCGCCTCTTGGCCGCTTTGGCTACCGGCGGCCACGTCCCGCTAGCCCAGGTGGCCAACGACCTGGAGAACCTCCGCAGCCTCCTGGGGGCTTGGGCCACCCATTTGGGGTGCCCCC

Q R L L A A L A T G G H V P L A Q V A N D L E N L R S L L G A W A T H L G C P P

400

| | | | | | | | | | | |

CCCCCCCCCCCGACCCCCCCCCGACCCCCCCCGGCCCCCCCCTGGCCGAAGCCCCCCACTCGGTGGCCACGGTGGCCCTGGAGCGGCTGCGGGGGTCCCTGGAGGGCATCGCGGGGACAC

P P P D P P P T P P G P P L A E A P H S V A T V A L E R L R G S L E G I A G T L

500 600

| | | | | | | | | | | |

TCGAGGGGCACCCCCCCTGC**TAG**GGACATCGGGGGGGGGGGCAGGGGGCACCCCCCACCCCGGGGGGGGCCCGGGGGACGTGTTGGGGGGGGGGGGGGGGgCCCaGGGGGGGGCCCGGGG

E G H P P C

700

| | | | | | | | | | | |

GGCGGGGGGGGGGGGGGGGGCGAGCGGGGGGGGGGGCGCCCCGGGGGGGCGGGGGGGGGGGGGGGTGGGGGGCCGGGGGGGGGGGGGTGGGGGGTGTGTTTGGGGGGGTTTGCACTCCAG

|

GGGGGGTCCCTTGG

Nucleic acid sequence model of the region containing the second coding exon in the leptin gene of the black-chinned hummingbird. The stop codon and the 3' dinucleotide at the RNA splicing site are shown in bold and indicated in blue and red respectively. The protein sequence of the leptin is shown typed in italics, below the nucleic acid sequence of the coding regions. Motifs matching with the consensus of G-quadruplex structures (G_3_+_N1-12_G_3_+N_1-12_G_3_+N_1-12_G_3_) are underlined and involved guanine stretches are shown in red.

**i. Coding exons of the leptin gene in the Ostrich (*Struthio camelus*) genome (70.22% GC content)**

100

| | | | | | | | | | | |

GGCGGTCGCGGGGGGGCACCGCACAGGCCCTGGGGACTTTGGGCCGCGCTTGCGAAAGGGCAGCTCCGGGAAGGCTCCCGGCACGGCCACCCTGGCACGTGCCGGCAGGGGACGGGGACA

200

| | | | | | | | | | | |

CCGCCGACACCATGTCTCCCCGGGCGCCTCATGTCACCCTCTTCCTCCCTGTGCACCCGGGATGGGGGGCAAGGTGCAGTGACACTCTCCGTTTTGTGATGCCTTGTTGCAAGAACCTGG

300

| | | | | | | | | | | |

GTGTTTTGTGTCCCACGTCCCCTTGGACGGGGGGACAGGGAGGATACGTAGCCCCCAGGGAGGGACGGGGTCAGCACCAAGACGGTGCCTGCGTGCCGCAGCAGCCCGGCGCCCCGGTCC

400

| | | | | | | | | | | |

CCTCCCCAGGGGCGCCGTGGGGTGGCACTTGCGCCCGGCGAGTGCCCGAGCGCCTCGGCGCGTCTCGCCCGGCAGAGCCCAGCAGG**ATG**CGGTGGCCCGGCGTGTCGCTCTGCGGGCTGC

S P G A P W G G T C A R R V P E R L G A S R P A E P S R *M R W P G V S L C G L L*

500 600

| | | | | | | | | | | |

TCTGGATGTGGCTCCCGCTCTCCTGCGGCCGCCCCGTGAAGATCGACAAGGTCAAGGCAGACACCAAGAACCTCACCCGGACCCTCATCGCCCGCATCCAGGAGTGCAGG**GC**AAGGGCCC

*W M W L P L S C G R P V K I D K V K A D T K N L T R T L I A R I Q E C R* A R A R

700

| | | | | | | | | | | |

GCAGGACGACGGGGCAGCGGGCGGTGGTAGGGACCCTGGCGGTCACCCCCGCCCTGACGTCCCCGCCATGGAGCCGCTGGGTCCCAGCAGGGACGGGGACAGCTCGGGGCTCAGAGGCAT

800

| | | | | | | | | | | |

CCTGGGTGTCAGCAGGGGGGACACTCGCCTGCAGCGGAGCAGGGGTGCCAAAGCGCTCCGGAGGGGCCCCGGCTCCCTGGGAAGGGGCAGACATCGCGGGCCTGGGCTGGGCTGTGACAG

900

| | | | | | | | | | | |

GGGGCCCTGTGCTCCTGAAGGGTCCGCAGGGAGGGACATGCACCCTGGGATGGGGTATGACGGGGTGGTCCTTTGGCCCAGGGGTTCCTAGAGAGGGACACCCACCCCAGGCTCAAGTGT

1000

| | | | | | | | | | | |

GCCAGGGTGTCCCTGCAGCCCAGGGGTCCCCAGGGAGGGACCTGCACCCCGGGCTGGGGTGTGCTAGGGTGTCCCTGCAGCCCAGAGTGGTCCCCAGGGAGGGACACCCATCCCAGGCTG

1100 1200

| | | | | | | | | | | |

GGGGGTACTGAGGTGTCCCTGCAGCCCAGAGGTCCCCAGGGAGGGACATGCACCCCGGGCTGGGGCTGGGTGCCAGGGTGTCCCTGCAGCCCAAAGGTCCCCAGGGAGGGACGTGCACCC

1300

| | | | | | | | | | | |

TGGGATGGAGCTGGGGCTGGGGTGTGCCGGGATGTCCCTGCAGCCCAGAGGTCCCCAGGCAGGGACGTGCACCCCGGGCTGGGGCTGGGGCTGGGGTGTGCCGGGATGTCCCTGCAGCCC

1400

| | | | | | | | | | | |

AGAGGTCCCCAGGCAGGGACGTGCACCCCGGGCTGGGGCTGGGCGCCGGGATGGCCCTGCACCCCGGGCAGGGAGCGGGTGCCGTGCCCTGACCGCAGCCCGCCGGTGC**AG**CTCTTCCCC

R G P Q A G T C T P G W G W A P G W P C T P G R E R V P C P D R S P P V Q *L F P*

1500

| | | | | | | | | | | |

CTGAACCTGAAGATCAGCGGGCTGGAGTTCATCCCCGGCGAGCGGGCGCCCGAGGGCCTGGCCGCCATGGACCAGCGGCTGCAGGGCTTCCAGCGGGTGCTGGCCGGCCTGCCGGCGGGC

*L N L K I S G L E F I P G E R A P E G L A A M D Q R L Q G F Q R V L A G L P A G*

1600

| | | | | | | | | | | |

AGCGTGCCGCTGGCGCAGATCGCCAACGACATGGAGAACCTGCGCAGCCTCCTGGCCGCCCTGGCCGCCCACCTGGGCTGCACCCTGCCCCTCGCCGCCACCGCCGCCGCCGCCGGCCCG

*S V P L A Q I A N D M E N L R S L L A A L A A H L G C T L P L A A T A A A A G P*

1700 1800

| | | | | | | | | | | |

GCCAACCTCGCCGATCTGCTGGCCGTCTCGCCCTACAGCGCCGCCGGGCTGGCCCTGGCCCGTCTCCGCGCCTGCCTCGACGGCATCGCCCGCCGCCTCGACGGCCTCCAGAGCTGC**TAG**

*A N L A D L L A V S P Y S A A G L A L A R L R A C L D G I A R R L D G L Q S C* *

1900

| | | | | | | | | | | |

CGCCCCGGGAGCTGCTGCCGGGAGCCCCGGGTGCCCCCCGGACGCGGCACAGCCGCAGAAAACTCCAGTCTCTTTTTT**ATTATTATTATTAATTATTATTATTATTATTACTATTATTAT**

2000

| | | | | | | | | |

**TATTATTATT**CACCCTTCATATTTATTTCTCCCGCATTAATTTAA**AATAAA**GCGAGTTTCCCAGCAGCCCGTCTCGCGTCGCTGAGCATCCCCGGGGCGGGGGGG

Nucleic acid sequence model of the region containing the coding exons split by a 841 bp intron in the ostrich leptin gene. The start and stop codons, the dinucleotides at the RNA splicing site and the putative polyadenylation signal are shown in bold and indicated in blue, red and green respectively. The protein sequence of the ostrich leptin is shown in italics, below the nucleic acid sequence of the coding regions. Statistically significant blocks in direct repeats were identified with MEME at http://meme-suite.org/tools/meme and are highlighted in yellow, grey and green respectively. Motifs matching with the consensus of G-quadruplex structures (G_3_+_N1-12_G_3_+N_1-12_G_3_+N_1-12_G_3_) are underlined and involved guanine stretches are shown in red.

**j. Coding exons of the leptin gene in the muscovy duck genome (79.36% GC content)**

100

| | | | | | | | | | | |

CGTGTCCCACTTGGCTCCGTGCCCCCGGCCACGTCACCTGCGGGGCGCGAGGCCTGGGGACCCCCCGGTGACGCCGCCCCCCGTGTGCCCCGCGGCGCACTCGGGGTATTTCGGAGGGGT

200

| | | | | | | | | | | |

GGGGGCACCGGGTGCTGACCCCCCCGTGCCCCCCCCCCCAGCCCCTGGCAGC**ATG**CGGTGTCACGGCGCGTGGCTCTGGGGGCTCCTGTGGCTGTGGCTGCCCCCGGCCGGCGGCCGCCC

G A P G A D P P V P P P P A P G S *M R C H G A W L W G L L W L W L P P A G G R P*

300

| | | | | | | | | | | |

CGTGCGCCCCGAAAAGGTTTGGGCGGACACCCGCAGCCTCGCCCGCACCCTCAGCGCCCGCATCCAGCTCCTGCAG**GC**AAGGCAGCGGCGCGGGGGGGGGCCACGGGGGGGTGGGAGTGG

*V R P E K V W A D T R S L A R T L S A R I Q L L Q* R Q R R G G G P R G G G S G

400

| | | | | | | | | | | |

GGGGGGGGAAGGTGGGCTGCGAGGGTCCCCCGGGGGGGGGGGGTTCGTGGCAACGAGGGGTGCGATGGAGATAGGGGGGTGGGTGTGGGGCCCTGTGCCACAGCGGGGGGGTTTTTTTNN

500 600

| | | | | | | | | | | |

NNNNNNNNNNNNNNNNNNNNNNNNNNNNNNNNNNNNNNNNNNNNNNNNNNNNNNNNNNN//NNNNNNNNNNNNNNNNNNNNNNNNNNNNNNNNNNNNNNNNNNNNNNNNNNNNNNNNNNN

700

| | | | | | | | | | | |

NNNNNNNNNNNNNNNNNNNNNNNNNNNNNNNNNNAGGGGGTCGCCCGCGGCCGCACGGGACCCCAAAGGGGATCCACAGGGACGGATGCGGGGGTGCCCCCGCCGCCCCCCCCGGTGACT

800

| | | | | | | | | | | |

CCCCCCCCCCCCGTCTCCTCGC**AG**CCTCTTCCCGCTGGGCCCGCGGGTGCTGGGGCTGGAGGCGATGCCCGGGGCGCGCCCCCCCGAGGGGCTGGGGGCGATGGAGCAGCGGCTGCAGCT

*P P P P S P R S L F P L G P R V L G L E A M P G A R P P E G L G A M E Q R L Q L*

900

| | | | | | | | | | | |

CTTCCAGCGCCTCCTGGGCGCCCTCCCCGCCGCCGCCGCGCCCCCCCCTCAGGTGCTGAGCGACCTGGAGAACCTCCGCAGCCTCCTGGCCGCCCTGGCCGCCCCTTTGGGTTGCGGCCC

*F Q R L L G A L P A A A A P P P Q V L S D L E N L R S L L A A L A A P L G C G P*

1000

| | | | | | | | | | | |

CCCCCGGCAACCCGAGGCGCCCCCCCCGGGGCTGGCCGAGCTCCTGGCCCAGGCTCCGCACACGGCCGCCGGCTTGGCCCTGGCGCGGCTCCGCGCCTGCCTCGACGGCATCGCCGCGCG

*P R Q P E A P P P G L A E L L A Q A P H T A A G L A L A R L R A C L D G I A A R*

1100 1200

| | | | | | | | | | | |

CCTCGACGCGGCCCCCGCCTGC**TAA**AATTTGGGGGGTGGGGGGGGGGCGTCAGGGGCCGGGGGGGACGTTGGGGACATCGAGGGACATCGAGGGACAGCGGGGGACAGGCAGGGACAGCG

*L D A A P A C* * N L G G G G G A S G A G G D V G D I E G H R G T A G D R Q G Q R

| |

GGGGTGCCGCGGGGAGCGGCGGCACG

Nucleic acid sequence model of the region containing the coding exons split by a 841 bp intron in the leptin gene of the muscovy duck. The start and stop codons and the dinucleotides at the RNA splicing site are shown in bold and indicated in blue and red respectively. The protein sequence of the leptin is shown in italics, below the nucleic acid sequence of the coding regions. Motifs matching with the consensus of G quadruplex structures (G_3_+_N1-12_G_3_+N_1-12_G_3_+N_1-12_G_3_) are underlined and involved guanine stretches are shown in red.

**k. First exon of the leptin gene in the white-throated tinamou, *Tinamus guttatus* (72.73 % GC content)**

100

| | | | | | | | | | | |

TGCGAAAGGGCAGCTCCGGGAAGGGCCCGGCTTGTCCCGGAGCGCCGGGACCCCGTTGCGGGAAGGTGTCCCGCATGTCACCTGGCCGGGTACGGGGACA**ATG**ACGCTGTCCCCCGCAGG

M T L S P A G

200

| | | | | | | | | | | |

ATGCGGGGACCCGGCGTGTCGCTCTGCGGGCTGCTCTGGCTCTGGCTGCCGCTGGCCTGCGGCCGGCCCCGTGAAGCTGGAGAAGGTCAAGGCGGATACCCGGAACCTGGCGCGCACCCT

C G D P A C R S A G C S G S G C R W P A A G P V K L E K V K A D T R N L A R T L

300

| | | | | | | | | |

CGGCACCCGCATCCAGGAGCTGCAG**GC**AAGAGCCGCGGGGGTCACCCCTGTCCCCAGGTCCCCACCGTGCTGCCCTGGGTGGGGTTGGGGACACCGTGGGG

G T R I Q E L Q

**l. Partial second coding exon of the leptin gene of the leptin gene in the brown kiwi, *Apteryx australis mantelli* (71.75 % GC content)**

100

| | | | | | | | | | | |

CTGTCTCTTATACACATCTAGATGTGTATAAGAGACAGGGGAGCGGGTGCCGTGCCCTGACGGCTGCCCGCCGGCGC**AG**CTCTTCCCCCTGAACCTGAAGATCAGCGGGCTGGAGTTCAT

L F P L N L K I S G L E F I

200

| | | | | | | | | | | |

CCCCGGGGAGCGGGCGCCCGAGGGGTTGGCCGCCGTCGACGAGCGGCTGCAGGGCTTCCAGCGGGTGCTGGCCGGGCTGCCGGCGGGCAGCCTGCCGCTGGCGCAGATCGCCAACGACAT

P G E R A P E G L A A V D E R L Q G F Q R V L A G L P A G S L P L A Q I A N D M

300

| | | | | |

GGAGAACCTGCGCAGCCTCCTGGCCGCCCTGGGCGCCCACCTGGGCTGCGCCCTGCCCCGCGCCGCCG

E N L R S L L A A L G A H L G C A L P R A A X

Nucleic acid sequence model of the region containing the first coding exon in the brown kiwi leptin gene (**g**) and the region containing the second coding exon in the white-throated tinamou leptin gene (**h**). The start codon and the dinucleotides at the RNA splicing site are shown in bold and indicated in blue and red respectively. The protein sequence of the leptins is shown in italics, below the nucleic acid sequence of the coding regions. Motifs matching with the consensus of G quadruplex structures (G_3_+_N1-12_G_3_+N_1-12_G_3_+N_1-12_G_3_) are underlined and involved guanine stretches are shown in red.
