# Supplementary material for: Sequence properties of certain GC rich avian genes, their origins and absence from genome assemblies: case studies
Source: BMC Genomics. 2019 Oct 14;20:734. doi: 10.1186/s12864-019-6131-1 (PMC6792250; doi:10.1186/s12864-019-6131-1)
Supplement: Supplementary file 10 — Additional file 10. Location of G-quadruplex structures in the 14 cDNA models described in [19]. [file 12864_2019_6131_MOESM10_ESM.docx]

Nucleic acid sequence model of the 14 cDNA models described (Hron et al 2015). Regions containing motifs matching with the consensus of G-quadruplex structures (G_3_+_N1-12_G_3_+N_1-12_G_3_+N_1-12_G_3_) are underlined and involved guanine stretches are shown in red on the plus strand and in blue on the minus strand.

>LPPR2

ATGGCCGCCCCCCGGCGGGAGGTGAAGAGGAGCAGTGGGATCGTGCCGTGCTTCCTCTGCGTGGAGCTGGGTATTTTGGGGGGCACGGCGGCCCTCGCCTACCAGCTGGAGTTCACCGACGCCTTCCCTGTGCACGAGGGGGGGTTCTTCTGCCGGGACCCCCAATATGGGCGCCCCTATCCCGGCCCCCCCGCCAACAGCCGCGCCCCCCCCGCCCTCGTTTACTCTCTGGTCACCGCCGTGCCCACCCTGACCATCGTGGTGGGGGAGCTGGCGGGGCGGCTGGGGGGGGCGCGGGGGGGCCGTGACCCCACAATTCTGTGGGGGGAGTGCTGCTATTTGGGGGCCCCACTGCGGCGGCTGCTGCGCTTCCTGGGCGTCTTCTCCTTCGGCCTATTGGCCACGGCCATCTTCGCCAACGCGGGGCAGGTGGTGACGGGGACCCCCGCCCCCCATTTCTTGGCCGTGTGCCGCCCCAACTACACGGCGTTGGGCTGCGCCCCCCCCCGCCCGGCCGCCCCCCCCCACTTCGTCACAGCCGGGGGGGCCGCGTGCGCCGGTGACCCCCCCTTGGTGGCCGCTGCCAGGAGGGCTTTCCCGTGTAAAGAGGCGGCGTTGGGGGCCTATGGGGGGGCGTACGCTGCGCTGTACGTGACGTTGGCGTGGCGGGGGGGGGGCTCCCGGCTGGCCAAACCCGCGGCCGTGTTGGCGTTGTGCGCCCCCCCCTTTTTGGTGGGGGCCGTCCGGGTGGCGGAGCACCGCAACCATTGGGCCGATGTGTTGGCCGGCTTCGTCACCGGCGGAGCCATCGCTGCCTTCCTGGTGAGCTGCGTGGTGGGCAACTTCCAGTGCAAGGGGGGGCCGCTGGGGGGGGGCTCAGGGGGGGGGGCGCCCCMCRGAGCCCCCCCAGAAGTGCCCCGACCTCAACCCCCCCTGGAGGAGCTGAGCGTCACCCAGACGCGCCGTGCTGAGTTCCCGGCCGTCACC

>MMP14

ATGGCGCCCGCTCTGCTGCTGCTGCTCCTCTGCTGCGCCGCCCCCCCCCCCGCCGCCGCCTTCCGGCCCGAGGCGTGGCTGCAGCAGTACGGCTATCTGCCCCCCGGCGACCTCCGCGCCCACCCCCCCACTTCGGCCCATTCGGTATCGGCCGCGTTGGCCGCCATGCAGCGCTTCTACGGGCTGCGCGTCACCGGAAGTGTCGACCCGGAAACGCTGCGGGCCATGAAGCGCCCCCGCTGTGGGGTCCCGGACCGCTTTGGGGCGGAGGTGAAGGCCAACGTGAGGCGCCGCCGTTACGCCATCCAGGGCTCCAAGTGGGAGCAGCGCGACATCACCTTCTGCCTTCAGAACCACACCCCGAAGGTGGGGGAGGCGGCCACCCGCGCTGCCATCCTCCGCGCCTTCGGGGTGTGGGCGTCCGTCACCCCACTGCGCTTCCGGGAAGTGCCCCCCGGCGCCGCCCCCCCCGCCGACATCGTCCTCTTCTTCGCCGAGGGCTTCCACGGCGACAGCTCCCCCTTCGACGGCGAGGGGGGGTTCCTGGCCCACGCCTACTTCCCCGGGCCCCACATCGGGGGGGACACGCACTTCGACGGCGCCGAGCCCTGGACCACGCGCAACGACGACCTCAGCGGTCACGACGTGTTCCTGGTGGCGCTGCACGAGCTGGGCCACGCGCTGGGCCTGGAGCACTCCAGCGACCCCTCGGCCGTCATGGCGCCCTTCTACCAATGGATGGACACCGCCCCCTTCGTGCTGCCCGACGACGACCGCCGCGGCATCCAGCAGCTCTACGGGCCGGGTCCCAACATGCCCCCCCCGGACCCCCGCGGCACAGCGCTGCCCCACGACCCCGACCGGCCGCCCCACGGCCCCCCCTATGGGCCCCGCATCTGCGACGGCGGCTTCGATACCATCGCGGTGCTCAGGGGGGAGATGTTCGTGTTCAAGGAGCGGTGGCTGTGGCGGCTGCGGGAGCGCCGGGTGCTGCCCGGTTACCCCCTCCCTATGGGGCAGCTGTGGCCCGGACTGCCCCACAGCATCGACGCCGCCTATGAGAGGAAGGACGGCAAGTTCGTCTTCTTCAAAGGCGGGCGGCAGTGGGTGTTCTCGGAGGCGGCGCTGCAGCCGGGCTTCCCGCGCGCTCTGCCGGACGTGGGCCGGGGGCTGCCGGAGCGCATCGACGCCGCGCTGCTGTGGCTGCCCAGCGGGGCCACGTACCTCTTCCGGGGCGACAAGTACTACCGGTTCAATGAGGAGACGGAGTCGGTGGACCCCGATTACCCCAAAAGCATTTCCGTGTGGGGCGGCGTCCCCGAATCACCCCAAGGAGCATTTATGGGGTCGGATGACGCCTACACGTACTTCGTGAAGGGCTCCCGCTATTGGCAGTTCGACAACCGCCAGCTGCGCGTCACCCCGGGTTACCCCAAATCCCTGCTCCGCGATTGGCTGGGCTGCCCGGAGCCCCGCCCACCACCCCGCCCTGGCCCCGCCCCTTCCTCTTCCCCGCCGGAAACGGGCGCCGGGGGGGGGGGAGGGGAAACGGAAGTCATCGTCATCGAAGTGGGCGGGGAAGGGGCGGGGCCCGGAGCGGTGGCCACGCCCCTGGCGCTGCTGGGGGGGGCCGGGGGGCTGCTGGCGGCCGTGCTGTGGTTCCGCCGCCGGGGGGCGCCCAAGAAACTGCTGCGCTGTCAGCGCTCCCTCCTGCCCCGCGTTTAG

>MRPL52

ATGGCGGCGCGCAAAGCGCTGCGGATCGCGGAGCTCCGCTCCCTCTCTGCCCGCCCCATTCCTGCGGCCCCACAGCGCATCGGCCAATGGCGCGTCAGCAAAGGCTTGGCCCCCGGCAGTTCGGGCTATGGGCCCCTCCGTGACCTCCCTGATTGGTCCTTTGTGGATGGCCGCCCAGCTCCCCTGTGGGCGGGGCAGCTGCGCCGTCGCCATGACAACGAGGAAGTTGCCCGCCGCGCCGTCGCTCTCATCCAATCAATGGACGCCGCTCGGGAGAGGGGGCGGGGCTTATCCCTCAAGCCCCGCCCCTCGCTGCGCCCCAAAGGCTCCGCCCCCAAATCAATAAAAGACGAATGA

>EPOR

ATGGCGGCTCCGGGGGTGCTGCTGGCGCTGGGGGGGGTCCTGGCGGCCGCGGGGGGGGGCGCTGAGACCCCCATGGACTTCGAGGTGGAAGCGGCGGTTCTGCAGGCGGAGGAGGCGGCGGACCCGAAGTGCTTCTCGCGGCGGCTGCACGACCTGCTGTGCTTCTGGGACAGCGACGGCCCCCCCGACCCGCAGCTCTTCCAGATGCACTTCCGCCTCGATTCGGATCCGTGGCAGCGCTGCCCGCTGAGCGCGGCGCGGCGGTCGGCGCTCCGTTCGCGCTTTTGGTGCTCCGTCCCTCCGAGCGCCGCCGTCGCCTTCGTGCCGTTGGAGCTGCGCGTTGTGCGCGCGCACAGCGGGGCCGCCGTGCACCGCCGGACGGTGTTCGTGGAGCGCGTGGTGCTGCTGGCCGCCCCGCACAACGTATCGGCGCACGCGGGCGGCGCTCCGGGCGCGCTGTGCGTTCGTTGGCAGCCGCCGCCCAACCCCTACTTGGAGTCGAGCCTCACCTACGAGCTGCTGCTGCGCGCCCCCGGGACGGCGCCGCGCACGGTGGGGGTCCCGGTGGGCCGCCTGGAGCAGCGGGTGGGGGCTCTGAGGGGTCGCACCCCATACACCGTCCGGGTCCGCGTCCGCCCCGACGGGCTGAGCTACGGCGGCTATTGGAGCCCGTGGTCCGAACCCATCACTGCCGTCACCGCCCCCGATGTGGACCCGGTGACGGTGGGGCTGTCCTCTCTGCTGGCGCTGCTGCTGCTGGGGCTGGCAATGCTCGCGCTGCTCGGACAGCGGCGGAAGCTGCAGGAGAAGCTGTGGCCGCCCGTGCCCGGCCCTGAGAGGGAATTCGAGGGGCTCTTCAGCGCCTATGGGGGCAATTTTCAGCTCTGGCTGTACCAAGGGGTGGTGGAGCCCTGGAGCCCCCCCGGAGGCACCCCGGAAGCCGAGGAACAGCCCAGTGCCGTGGAAGAGGTGGGGCCCCCCCCGGGCAAAGAGCCCCCCCCGGGGACCCCCCCGTCTGCCCCCCCTTCGGCCCCCCCCAGCGGCCCCTCGCCCGCCTCCAGCTTTGAGTACACGCTGTTTGACCCCGGCTCGGCCCTGCTCTGCCCCAGGGGGCACCCCCAAATCGCCCCCCCCCACGATCCCCCCGGCGGCCCCTACGCCAACCTGGCCCCCCCCCACAAAGGGCCCCCCCCGCCCGAGGAGGGGACCCCCAAAGAAACCCCCCACGACAGGAGACCCCCACGGGAGCTTCCGTGCAATGGGAACCCCCCCGGGACCCTCCTGGCGTTGGGGCCCCCCCCAATGCCCCCCCCCTACGTGCTGTGCTCTTAA

>EPO

ATGGATGTCAATGGGGCCGGGCTGTGTGCGGTGCTGCTGCTGCTGCTGCTGCTGCGGGGGGGGGGGGGGGGGCGCCCCGACGGCCCCCCCTCACTGTGTGACCCCCGAGTGATGGAGAGGTTCATCCGGGAGGCGCGCGACGCTGAGAGGGGGATGGTCGGCTGTGGGCGGCGCTGTGATCTCCCCGAGGCGGTGGCCGTCCCGGACCCCGGCGTCAGCTTCAGCGAATGGCAGCGCATGGATGTGGGGGCTCGGGTTCGGGCGGTGCTGGGGGGCCACGCGGTGTTGGTGGCTGCGGTGCTGCGGGCGCGGGAGCTGCTGAGCGACCCCCAACTCCGACCCACACTGGATCTGATCTATGGGGCAGCACGGAGTCTGGCACACCTGCTGAGGGGGGTGGTCAGCCCGCCCACCCCCACCCCCACCCGCACCCCCCACTCTCCCACCCCCACCCCCTTTTCTCCCACCCCCTCTTCTCCCACCCCCTTTTCCCCCCCTTCCTCCCCCCACTCCGCCCCCCCCCCGCCCCCTCCCCCCCCCCAGGTGAGGACCCTCAGCCGCCTTTTGGGGGTCCACAGCGCTTTCCTCCGTGGCAAAGTCCGGCTGCTGCTCATTGATGTCTGCACCCCGGTGTCCCCCCCCCGGCACTGGCGGTGA

>SWSAP1

ATGGCGGCAGCGCTGGAACGGGCGCTGGGCCCGGCTGGGCCGGAAGCGGACGCGTCCGTGCCGGAGGCGCCGCTGTTGGTGCTCGGCCCGGCGGGCTCGGGCCGCACGGCGCTGCTGCTGCGGGCGGCGTTGGCGGGAGGAGGGGACGGGCCCCGCGCTCTCTTCTTAGCGCCCAGCGCTCCCTCCCGACTCCCAGACGGCGGCGGCGGTGACCCGAGGGCGCTGCAGCGCCTGGAGCTCCGCTACCCCCCCACCCTGGCAGCCCTGGCCCAGGAGTTGGGGGCAATGGCGGCCCGAGCCCGGCCCCCCGGCTTGCTGCTGCTGGACGGGCTGGAGCACTACATCCAGGGGGGGCCCAGCGCCCCCGCCCGCCTGGCCGCCCTGCTGCTGGAGGCCTCCCGCGCCCCCCGACCCCCGGCCCGGCTCCTGGCTGCCCTCCGTGTGCCCCCCCCCGGGCCCCGTGTGCTGCCCGTCCTGCGCCGTTACTTCCCGGCTGAGTGCCGTCTGAGACCCCTCCCCGGGGTCCCACTGCAGGTGAACGTCCGCCTCGTCCTGCCCGGATCGGTGCCGCGGGGATGGAGGCTGCGCTTCGAGCCCCACGGGGGGCTGAACGTCACCCCCGGGCCTGGGGACGGCGATGGGGACGAGGATGAGGACGGAGATGGCAGTGGGGATGAAGGCTGA

>POP7

GGGTCCCGAAATCCGCCTCCACGGGTTGGGTTTGGCCGTCACCCGCGCCATCAACCTGGCCCTACAACTGCAGGCGGCCGCTCCCGGCGCCCTACAACTCCATGCCAGCACCTCCTCCGTCACCCTGCCCGGACGGGGGGGCAACGGGGAACCCCCCCCCCCCCACCCCCACCACCCCCACGACGATGAGGACCCCGATGCCCCCCCCCGGGACGACTCCCCCGACGCCGCTGCCCTTCGCCCCCGGCACAACTCGGCCATCCACATCCGTGTGTGCCGCGAGGCGCCGTGCGTCTGA

>ALKBH7

TGGGGCCGGGGGGGCGCTCTGGGGTCAGAGGTCATCGAGTCAGAGGTCACCCCCGGGATGGGGCCATTTCCGGGTTCCGGGAGGCGGAGCGCAGCCGTTGGGGGGCGCTGTCGGGGGCGGTGCTGCAGCGGATCTCCTCCGCGTTTCCCCCCGCGCGGCCCCCACTGCCCCACAGCCACATCCTGGACCTCGCGCCCCACGGCTGCGTGCGGCCCCACATCGACAGCACCAAGTTCTGTGGCTGCACCATCGCGGGGCTCTCTCTGCTGTCGGCGGCGGTGATGCGGCTGCGCAGCGTTGGGGACCCCCAGGAGTGGGCAGAGCTGCTGCTGGAGCCGCTCTCCCTTTACGTCCTGCGGGGTGAGGCGCGCTATGGGTTCACCCACGAGGTTTTGGGGGGGGAGGAGTCCTTTTTCGGGGCCCTCCGCGTCCCCCGGCAGCGCCGATTGGCCGTCATCCGCCG

>BLVRB

ATGGATCGCGATCGGATCGTGGCGCTGTTCGGGGCCACCGGGAGGAGCGGCCGGGAGGCGCTGCGGAGGGCGCTGCGGGAGGGCTACGCGGTATCGGCTCTGGTTCGGAACCCGGCGCTGCTGCCGCCCGACGCCGCGCCGTGCCGGGTGGTCCGCGGGGACGCGCTGCGCGCCGCCGACGTCAGCGCCACCGTGCGGGGGCAGCGCGCCGTCATCGTCACGTTGGGAACGCGCGGAGACATCGGTCCCACCACCGTCCTATCAGACAGCACCCGCAACATCGTGGCCGCCATGAAGGAGCACGGCGTGCGCAAAGTGGTGGCGTGTCTGTCCGCCTTCCTCTTATGGGATCCTGAGAAGGTCCCCACGCGGCTGCGGGCGCTGACGGAGGACCACGCGCGGATGCACGCCGTGCTGAGCGGGGCCGGGCTGGATTACGTGGCCGTCATGCCGCCCCACATCGCCGACGACAAGCCGCTGACGGAGGCATACGAGGTCACGGTCGGTGGCACCGGCGGTGGCTCGCGGGTCATCTCCACGCCGGACCTGGCCCATTTCCTCGTGCGCTGCCTCAGCACCACCGCGTTCGACGGGAAGAGCGTCTACGTCTGCGGGCACTACGGCTGA

>INO80E

ATGAACGGAGCGGCGGATCCCGATGGCGGCGGAGGGAGCGGTTGCCGGCGGCGATACCGCGCCTTGAAGCGGCGGCTGCGGCTGCTGCTCTACGAGCAGGAGTGCTTCCAGGAGGAGCTGCGCCGCGCTCAGCGCCGATTGCTGCGCGTCTCCCGGGATAAGAGCTTCCTGCTGGACCGCCTGCTGCAGTACGAACACGTGGACGACGACTCCTCAGATTCCGACGCCACCGCCTCCTCCGACAGCGATGGGGAAACGCCCAAAGGGGCGGAGCCGCCTCCCCTTAAAAGGAAGCGCAGCCCCACGGGGGGCGGAGCCTCCCCACCGCCCGCCCCCGGCCTGGCCCCGCCCACTTCCTACTTGAGCACGCTGGCCTCCCCCCCATACaGCCCcTTCCCGGCTGATTACCTGGCCCCCCCCGAGCGGCCCCGCGGCCCCACACGGCGCAATAAGGGATCCCGGCGCCTGCAGCTCCCCGCCGCCCCCCCCCCGGCGCTGCCTTTCCCCCCTCCCCGCGTTTTGGGGGGGGGTTCCGTGGCCGCCCCCCCGCCCCCCCCCAAAGCCCCCGGCCCGGTGCCCCACACCGTCCCACGGCGTCTGCTCAGCGATGGGGGGGACGGCAGCGGAGACGATGGCATGGATGGGGACGACGAGCTCGTCATCGACATCCCCGAGTGA

>NDUFB7

ATGGGAGCTCACCTGGCGCGGCGCTATGCCGGGGGGGCGGACACGGAGCCGGACCCGTTGGCGATGCCCACATTCCCCGCCGATCTGGGGCTGCCCGGCAGGGAGCCGCGCACCATGGTGGCGACGGCGCAGCAGCTGTCGGAGGGCCGCGTCCCGTTGGCTCAGCGCGACTTCTGCGCCCACCACCTCCTGCGCCTCATGCGCTGCCGCCGCGACGCCTTCCCCAGCCTATGGCACTGCCACCACCTGCGGCACCGCTGGGACCGCTGCCAGCACGACGATTATGTGATGCGCATGAAGGAGTTTGAGCGCGAGCGGCGGCTGCTGCAGCGTCAGAGGCGATCCGGGAGCGGGAGGCGGCCGTGGCTGCAGAGTGA

>OPLAH

ATGGGTCCCAGCAGTCCCTATGGGGCCaATGCGTCCCCTATGGGGCTGAGGTCGGTGTGTGCCCCACAGAGCGGGGTGGCGGTTCCGGAGCGGGGCCCGCTGGACTCCCGACACGTGCAGTGGAtTCCGGATGGGGCCGAGTGcTCCtCtAtggGgcCCaGCAGtcCCtatGGGgcCGagtGctcCTcTATGGGTCcCAGCASCGRCTTCCGGGACCTTCTGCACATCGGCACCCAGGCCAGGCCCGACATCTTTGACCTGACGGTGTCGGTGCCCCCCCCGCTGTACGAGGCGGTCATAGAGGTGGACGAGCGGCTCATCCCGGCGCAGCCGCACTGCCGTCTGCCGGGCGCTCAGCGCGGGGAGCGCCGCACCGGTCGCAGCGGGGACGAAGTGTTGGTGCTGCGGCGCCCGGACGTGGAGGCGCTGCGGGCGGAGCTGCAGAAGGTGTGGGAGCGCGGAGTGCGCAGCGCCGCCGTCCTTCTGCTGCACTCCTACACCTGCCCGGATCACGAGGCTGAGGTGGGCTCCCTGCTGAGCTCCATGGGTTTCCGCCACGTGTCGCTGTCGTCGGCGCTGTCGGCCATGGCGCGCGCGGTGCCGCGGGGGATGACGGCATGCGCCGATGCGTACCTCACCCCCTGCCTGCAGCGCTACCTGCGCGGCTTCTGCCACGGCTTCAGCGACGGCCTCCAGGGGGTCCCGGTGCTGTTCATGCGCTCGGATGGGGGGCTGACCCCCATGGCGCAGTTCAGCGGTGCCCGCGCGGTGCTTTCCGGGCCGGCGGGGGGCGTTGTGGGGTACAGCCGTACCGCGGGGGGGCTCCGGGAACAGCGGCCCGTCATTGGCTTCGATATGGGAGGGACGTCGACGGACGTGAGCCGCTTTGCGGGGCGCTTGGAGCACATCTACGACGGCGTCACGGCGGGGGTCTGCATCCAGAGCCCACAGCTCGACCTGCACACCGTGGCGGCCGGGGGGGGGTCCCGTCTCTTCTACCGTTCCGGTCTCTTTGTGGTCGGCCCCGAATCCGCGGGCGCAAATCCCGGCCCCGCGTGTTACCGAAAAGGCGGCCCGGCCACGGTGACGGACGCCAACCTGGTGCTGGGCCGCCTCCTGCCCGCCTTCTTCCCGCACATCTTCGGGCCGTCGGAGGACCAACCGCTGAGCCTGGAGGCCGCCCGCAGCGCCCTGCGGGACCTGGCGGACGCCGTGGCAGCCGACGGCCACGAGGGGGCGCCGCTGAGCCTGGAGGAGGTCGCCATGGGCTTCGTCCGCGTGGCCAACGAGGCCATGAGCCGCCCCATAAGGGCGCTCACCGAGGCTCGGGGTCACCCCGTGGGGCAGCACATCCTGGCGTGCTTTGGGGGCGCGGGAGGGCAGCACGCGTGTGCCATCGCACGGGCCCTGGGCATGGACAGCGTCTTCATTCATAAACACAGCGGGGTGCTGTCGGCCTTCGGGCTGGCGCTGGCCGATGTGGTGCACGAGGCTCAGGAGCCGTCGGCGCTGCGCTACGAGGCGGCCGCGTTCGCCGCGTTGGATGAGCGCGTGGAGGCGCTGCGGGAGCGCTGCTGCGCCGCGCTGCGGGAGCAGGGATTCAACAGCTCTCAGATCCAGACGGAGCCGTTCCTCCACCTGCGCTACGCGGGGACGGACTGCGCCCTGATGTGCTCCGCTGTGGGGTACCCCCCGACCCCAAATTCCTGCCGCGCCGGGGACTTCGGGGCCGCCTTCGCTGAGCAATACCGCACGGAGTTTGGGTTCACCATCCCGGACCGGGCGGTTCTGGTGGACGACATTCGGGTTCGGGGGGTCGGCAGCAGTGGGGTCACCGAGGAGACCCCAAACCCCAGAAGGGGGGAACCCTCCGGGCCGGAGACGGTGACGCGGTGCTACTTTGAGGGGGGCTTTCTGGACACCCCGGTATTTCTGATGGAGGGGCTGAGCTGTGATCACCCCCTTCCCGGCCCCGCCATCATCATCGACCGCCACAGCACCATCGTGGTGGAGCCGGGCTGCGTGGCGGAGCTGACGCCTATGGGGGACATCCGCATCGCCGTGGGGCGCCCGACCCCACTGGTTGTGGGGCCGCAGCTCGACCCCGTGCTGCTCTCCCTCTTCTCACACCGCTTCATGAGCATCGCAGAGCAGATGGGGCGCGTCCTGCAGCGCAGCGCCATCTCCACCAACGTGAAGGAGCGCCTCGACTTCTCCTGCGCCGTCTTCGGGGCCGGCGGCGAATTGGTGTCCAACGCGCCCCACATCCCCGTGCACCTGGGGGCCATGCAGGACGCCGTGCAGTTCCAGATCCGCAGTGTTGGTGCTGATCTGCAGCCTGGGGACGTCCTGCTGAGCAACCACCCCATAGCAGGGGGCAGCCACCTCCCCGACCTCACCGTCATCACCCCCGTGTTCTGGCCGGAGCTGTCGGCGCCGGTGTTCTGGGTTGCCAGCCGGGGGCACCATGCGGATATTGGGGGGCTGACGCCGGGTTCGATGCCCCCCCATTCGAAGACCCTGAGTGAGGAGGGGGCCGTCTTCATCTCCTTCCATCTCGTGAGGGCCGGAGTGTTCCAGGAGGAGGCGGTGTCGGCGGTGCTGCAGGAGAGCGGTACGCGGGCGCTGCGGGACAACGTGGCGGACCTCCGAGCTCAGGTGGCCGCCAACCACAAGGGGGCGACACTGCTGCGGGAGTTGGTGGCCGCCTATGGGCTGAGCGGCGTCACCGCCTACATGGAGCACATCCGGGCGAACGCGGAGCGGTCGGTGCGGGAGATGCTGCGGGGGGCGGCGCGGCGCTGGGGGGCGGTGATGGAGGCGGAGGATCGAATGGACGACGGGACCCCCATCTGCCTGCGGGTCACTGTGGACCCCACTGAGGGCAGCGCAGTGTTTGATTTCTCGGGTTCGGGCCCGGAGGTTTATGGGAACTGCAACGCCCCGCGGGCCATCACACTGTCAGCCCTCATCTACTGCCTGCGCTGCATGGTGGGCCACGACATCCCCCTCAACCAGGGCTGCCTGGCCCCGGTGCGGGTGCTCCTTCCGGAAGGTTCCATCCTCAGCCCGTCCCCGCAGGCGGCGGTGGTTGGGGGCAACGTGCTGACATCGCAGCGCATCGTCGATGTGGTGCTCAGAGCCTTCGGGGCCTGCGCGGCGTCGCAGGGCTGTATGAACAACGTGACGTTCGGCGACGCGTCCATTGGTTACTACGAGACGGTGGCGGGCGGGGCCGGCGCGGGGCCGCATTGGGCCGGGCGCAGCGGCGTGCACAGTCACATGACCAACACGCGCATCACCGACCCCGAGATCCTCGAGCTGCGTTACCCGGTGGTGGTGCGGCGCTTTGAGCTGCGCCGCGGTTCGGGCGGTTCGGGGCGATTCCGGGGCGGTGACGGAGTGAGGAGAGAGCTGCAGTTCAGAGCCCCCCTCGTGCTGTCCGTGCTCAGCGAGCGCCGCGTCACGCAGCCCTACGGCATGCAGGGCGGAGCTGCGGGCGCCCGCGGGGTGAATCTGCTGCAGCGCTGCGACGGCCGCGTGCTCAGCCTGGGGCCCAAAGCCTGCGTCAGCGTGGGGCCGGGGGACGTATTCATCCTTCTGACCCCCGGCGGTGGCGGCTTTGGGACCCCCGAGGAGGATGGGGGTGAAGGGGGGGCGCACAGCCCCAAACCCACCGGGGCCCGCGAGTACTGGGAGGGCACTGAGGCACACTGA

>PCP2

ATGGGTGGGGGTCTCCCTGAGCGCCGTGTTTGCCGCGGGGCGGAGCCCCGGAGCGGGGGGGGTCTCCGGACGGGGGGTGGGTCCCCGGAGGGCCAGGAGGGCTTCTTTACCCTCCTGAGCTCCGTGCAGGGGGCCCGCATGGATGAGCAGCGCTGCAGCCTGGGGGGGGGCGGGCCCCCCCCCGAGCTGGCCACCCTGCTGGATTTGGTCGCCCACTCTCAGGGCCGCCGATTGGACGAGCAGCGCCTGGGGGTGCAGCGGCTGCCGGGTTTTGGGGGGCCCCCCCCGGATGGAAGCACTGCGAGCGGGGATGGGAA

>PET100

ATGGGGGTGAAGTTGGAGGTGTTCCGGATGCTGCTGTACCTCTCGTTCCCCGTCGGTGTCTTCTGGGTGTCCAATCAGGCTCAGTACTTCCAGCAGTTCGTCGTGCAGCGCAGGAGAGAGATCTTCCCTCCGGACAACCCCGAGCGGCGCCGTGAGGTGGCGGCGCTGAAGCAGCGGGTGCTGCGGATCCAGGAGGAGCGCGCGCTGCGGGACACGCGTGGGTAG
